# Halogenated volatiles from the fungus *Geniculosporium* and the actinomycete *Streptomyces chartreusis*

**DOI:** 10.3762/bjoc.9.311

**Published:** 2013-12-03

**Authors:** Tao Wang, Patrick Rabe, Christian A Citron, Jeroen S Dickschat

**Affiliations:** 1 Institut für Organische Chemie, TU Braunschweig, Hagenring 30, 38106 Braunschweig, Germany

**Keywords:** constitutional isomerism, GC-MS, natural products, organohalogen compounds, volatiles

## Abstract

Two unidentified chlorinated volatiles **X** and **Y** were detected in headspace extracts of the fungus *Geniculosporium*. Their mass spectra pointed to the structures of a chlorodimethoxybenzene for **X** and a dichlorodimethoxybenzene for **Y**. The mass spectra of some constitutional isomers for **X** and **Y** were included in our databases and proved to be very similar, thus preventing a full structural assignment. For unambiguous structure elucidation all possible constitutional isomers for **X** and **Y** were obtained by synthesis or from commercial suppliers. Comparison of mass spectra and GC retention times rigorously established the structures of the two chlorinated volatiles. Chlorinated volatiles are not very widespread, but brominated or even iodinated volatiles are even more rare. Surprisingly, headspace extracts from *Streptomyces chartreusis* contained methyl 2-iodobenzoate, a new natural product that adds to the small family of iodinated natural products.

## Introduction

Today several thousands of halogenated natural products are known, most of them being produced by marine organisms [1]. The majority of halogenated natural products are chlorinated or brominated, while iodinated and particularly fluorinated compounds are significantly less abundant (Table 1) [2]. Reasons for this distribution are (I) the different concentrations of the halogenides in sea water and (II) the differences in their redox potentials [3]. In terrestrial habitats local halogenide concentrations can strongly differ from those found in marine ecosystems, but the overall trend is similar, and therefore chlorine and bromine are also the most widespread halogens in natural products from terrestrial organisms.

**Table 1:** Approximate number of known halogenated natural products and redox potential and natural abundance of the halogenides in sea water.

Halogenide	Natural products^a^	Redox potential [V]^b^	Sea water [mg L^−1^]^b^

F^−^	30	+2.87	1.4
Cl^−^	2200	+1.36	18100
Br^−^	1900	+1.07	68
I^−^	100	+0.54	0.06

^a^Estimated number of known natural products in 2002 according to reference [2]. ^b^Redox potentials and concentrations in sea water as given in reference [3].

Many biologically active halogenated secondary metabolites are known from actinomycetes including vancomycin [4], chloramphenicol [5], rebeccamycin [6], marinopyrrols [7–8], armeniaspirols [9], and salinosporamide [10]. Also a few volatile halogenated compounds are known, e.g., from algae as summarised in a recent review [11]. Particularly interesting is the production of chloroform by termites that was estimated to account for 15% of the global chloroform emissions [12].

The introduction of halogens into natural products is catalysed by various known types of enzymes [13–14], including the FADH_2_-dependent halogenases [15], α-ketoglutarate/Fe^2+^-dependent halogenases [16], SAM-dependent halogenases [17–18], and vanadium-dependent haloperoxidases [19].

Here we report on the identification of two chlorinated volatile compounds from the fungus *Geniculosporium* and a iodinated volatile from *Streptomyces chartreusis*.

## Results and Discussion

### Chlorinated volatiles from *Geniculosporium*

**Figure 1:**
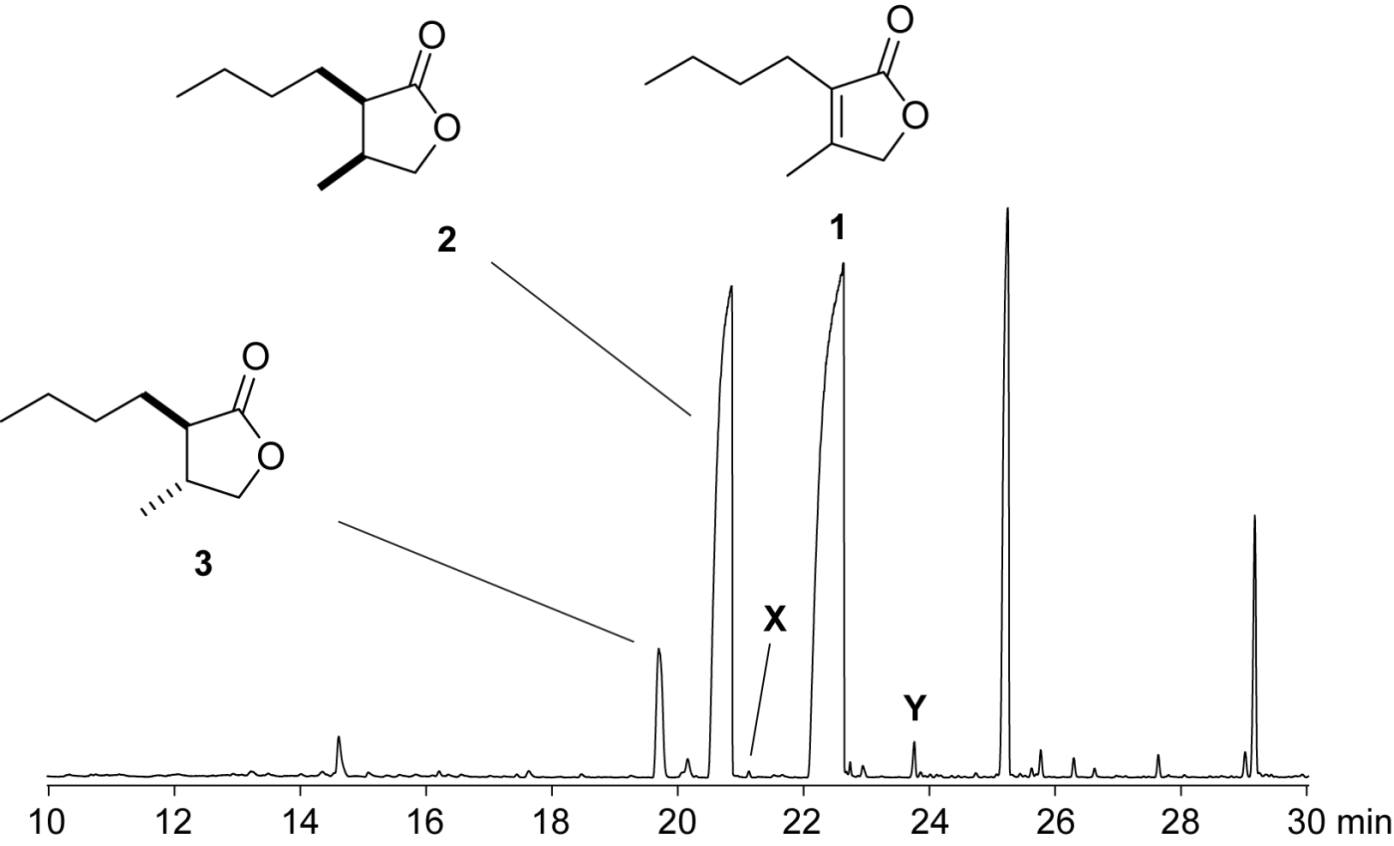
Total ion chromatogram of a CLSA headspace extract from *Geniculosporium*.

**Figure 2:**
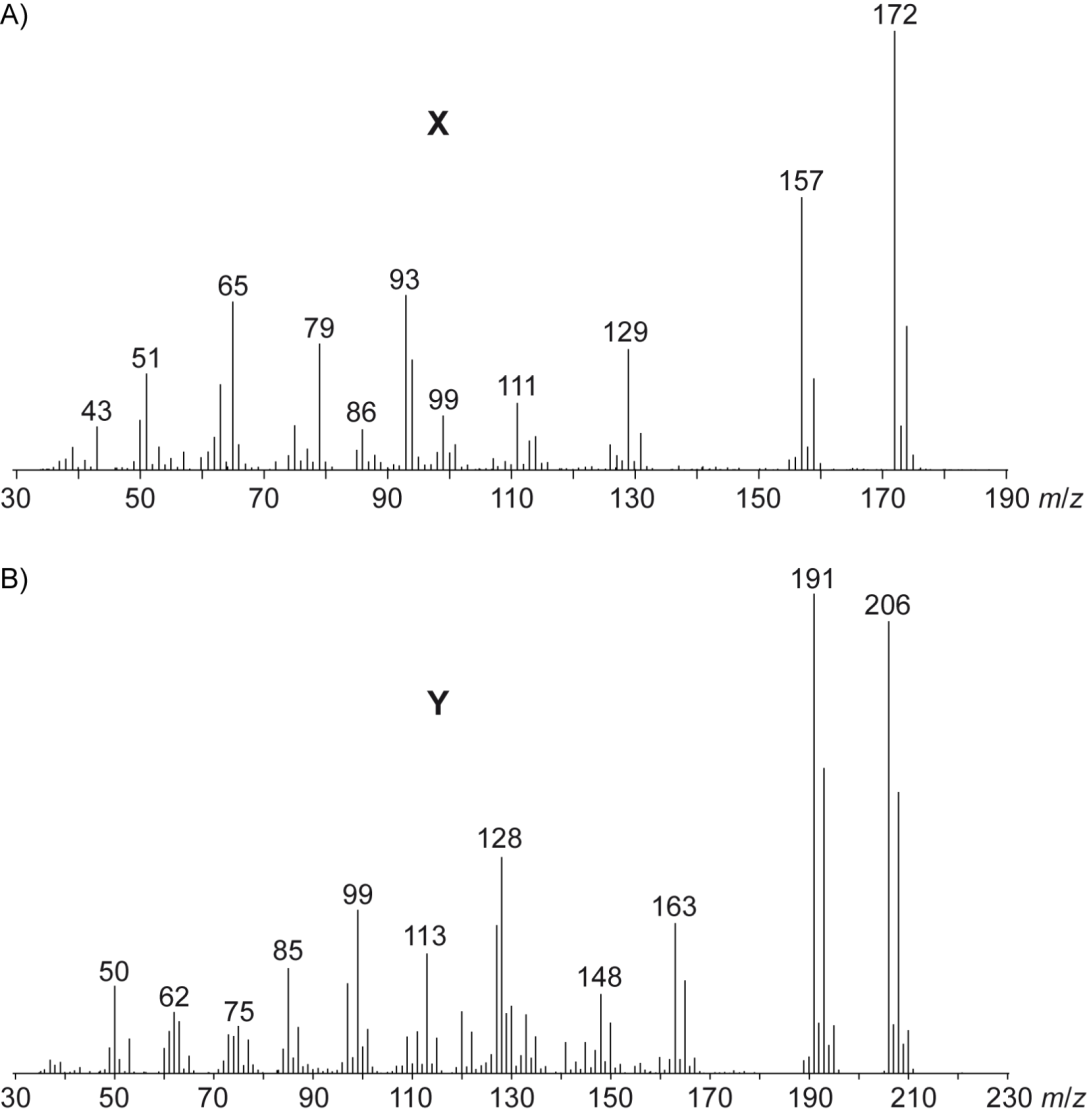
Mass spectra of A) the chlorinated volatile **X** and B) the chlorinated volatile **Y**.

The volatiles emitted by agar plate cultures of the fungus *Geniculosporium* sp. 9910 were trapped on charcoal filters by using a closed-loop stripping apparatus (CLSA) [20]. After one day of collection a solvent extract of the trapped material was analysed by GC–MS. The analytical process was performed three times to demonstrate reproducibility of the results, and a representative chromatogram is shown in Figure 1. Besides the main compound **1** and some structurally related volatiles **2** and **3** that were identified by delinating a structural proposal from the mass spectra followed by synthesis of reference compounds [21], two additional volatiles **X** and **Y** were observed in all investigated samples. Their mass spectra are shown in Figure 2.

Both volatiles were suggested to be chlorinated compounds due to their characteristic isotope patterns of the molecular ions with compound **X** containing one chlorine and volatile **Y** containing two chlorine atoms. Furthermore, a comparison of the mass spectrum of **X** to data base spectra revealed strong similarities to mass spectra of chlorodimethoxybenzenes, but the data base mass spectra of various constitutional isomers showed only marginal differences. Similarly, the mass spectrum of **Y** was very similar to data base spectra of dichlorodimethoxybenzenes, but also in this case the mass spectra of several constitutional isomers were highly similar. Therefore, the comparison of mass spectra was not a sufficient criterion for unambiguous compound identification, and thus the additional comparison of GC retention indices of both natural products to authentic standards of all possible structural isomers was pursued.

**Figure 3:**
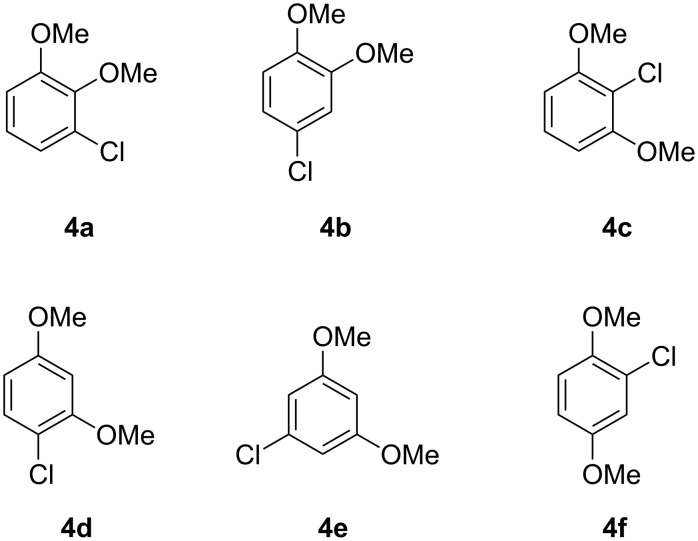
Constitutional isomers of chlorodimethoxybenzene as candidate structures for **X**.

**Scheme 1:**
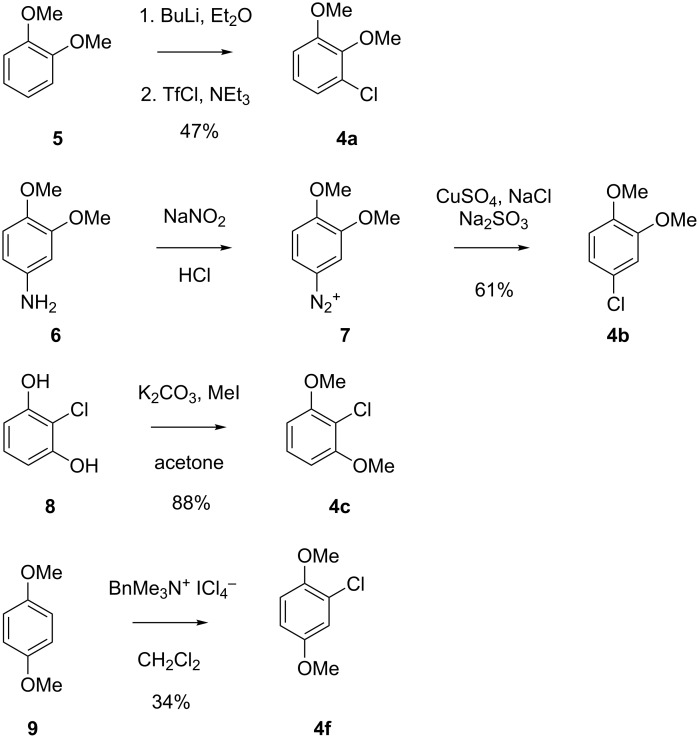
Synthesis of chlorodimethoxybenzenes as reference compounds for **X**.

The six constitutional isomers of a chlorodimethoxybenzene **4a**–**4f** as candidate structures for **X** are summarised in Figure 3. Compounds **4d** and **4e** were commercially available, while the remaining isomers were obtained by synthesis according to Scheme 1. Surprisingly, no satisfying synthetic procedure for compound **4a** was available from literature, but *ortho*-lithiation of veratrole (**5**) followed by treatment with trifluorosulfonyl chloride and triethylamine gave acceptable yields (47%). Conversion of 4-aminoveratrole (**6**) into the corresponding diazonium salt followed by a Sandmeyer reaction resulted in **4b** (61% over two steps). The isomer **4c** was efficiently prepared by double methylation of 2-chlororesorcinol (**8**) with potassium carbonate and methyl iodide in acetone (88%), while **4f** was obtained by chlorination of dimethoxybenzene **9** with benzyltrimethylammonium tetrachloroiodate in moderate yield (34%) [22]. All six compounds were subjected to GC–MS analysis using the same type of GC column as for the headspace extract from *Geniculosporium* (HP5-MS) and their retention indices together with the NMR spectroscopic data are summarised in Table 2. Mass spectra, ^1^H and ^13^C NMR spectra of all six compounds are given in Supporting Information File 1. The structure of **4b** was unambiguously assigned to the natural product **X** by comparison of mass spectra and retention indices (Table 2).

**Table 2:** NMR spectroscopic data and GC retention indices of all constitutional isomers of chlorodimethoxybenzene in comparison to natural **X**.

Compound	^1^H NMR	^13^C NMR	*I*

**4a**	6.96 (m, 2H, 2 × CH) 6.81 (m, 1H, CH) 3.87 (s, 3H, OCH_3_) 3.86 (s, 3H, OCH_3_)	154.0 (C_q_) 145.5 (C_q_) 128.4 (C_q_) 124.3 (CH) 122.0 (CH) 110.9 (CH) 60.6 (OCH_3_) 56.1 (OCH_3_)	1278
**4f**	6.95 (d, 1H, ^4^*J*_H,H_ = 3.0 Hz, CH) 6.86 (d, 1H, ^4^*J*_H,H_ = 3.0 Hz, ^3^*J*_H,H_ = 9.0 Hz) 6.76 (dd, 1H, ^3^*J*_H,H_ = 3.0 Hz, ^4^*J*_H,H_ = 9.0 Hz, CH) 3.84 (s, 3H, OCH_3_) 3.75 (s, 3H, OCH_3_)	153.8 (C_q_) 149.4 (C_q_) 123.0 (C_q_) 116.1 (CH) 113.2 (CH) 112.8 (CH) 56.7 (OCH_3_) 55.8 (OCH_3_)	1292
**4b**	6.87 (dd, 1H, ^3^*J*_H,H_ = 8.5 Hz, ^4^*J*_H,H_ = 2.4 Hz, CH) 6.84 (d, 1H, ^4^*J*_H,H_ = 2.4 Hz, CH) 6.76 (d, 1H, ^3^*J*_H,H_ = 8.5 Hz, CH) 3.85 (s, 3H, OCH_3_) 3.84 (s, 3H, OCH_3_)	149.5 (C_q_) 147.8 (C_q_) 125.6 (C_q_) 120.2 (CH) 112.0 (CH) 111.9 (CH) 56.02 (OCH_3_) 55.98 (OCH_3_)	1322
**4e**	6.51 (d, 2H, ^4^*J*_H,H_ = 2.2 Hz, 2 × CH) 6.34 (t, 1H, ^4^*J*_H,H_ = 2.2 Hz, CH) 3.77 (s, 6H, 2 × OCH_3_)	161.1 (2 × C_q_) 135.2 (C_q_) 106.9 (2 × CH) 99.2 (CH) 55.5 (2 × OCH_3_)	1340
**4d**	7.24 (d, 1H, ^3^*J*_H,H_ = 8.7 Hz, CH) 6.50 (d, 1H, ^4^*J*_H,H_ = 2.7 Hz, CH) 6.42 (dd, 1H, ^3^*J*_H,H_ = 8.7 Hz, ^4^*J*_H,H_ = 2.7 Hz, CH) 3.87 (s, 3H, OCH_3_) 3.79 (s, 3H, OCH_3_)	159.5 (C_q_) 155.6 (C_q_) 130.1 (CH) 114.1 (C_q_) 105.2 (CH) 100.0 (CH) 56.0 (OCH_3_) 55.5 (OCH_3_)	1374
**4c**	7.17 (t, 1H, ^3^*J*_H,H_ = 8.4 Hz, CH) 6.60 (d, 2H, ^3^*J*_H,H_ = 8.4 Hz, 2 × CH) 3.90 (s, 6H, 2 × OCH_3_)	156.2 (2 × C_q_) 127.1 (CH) 110.6 (C_q_) 104.7 (2 × CH) 56.3 (2 × OCH_3_)	1404
**X**			1322

**Figure 4:**
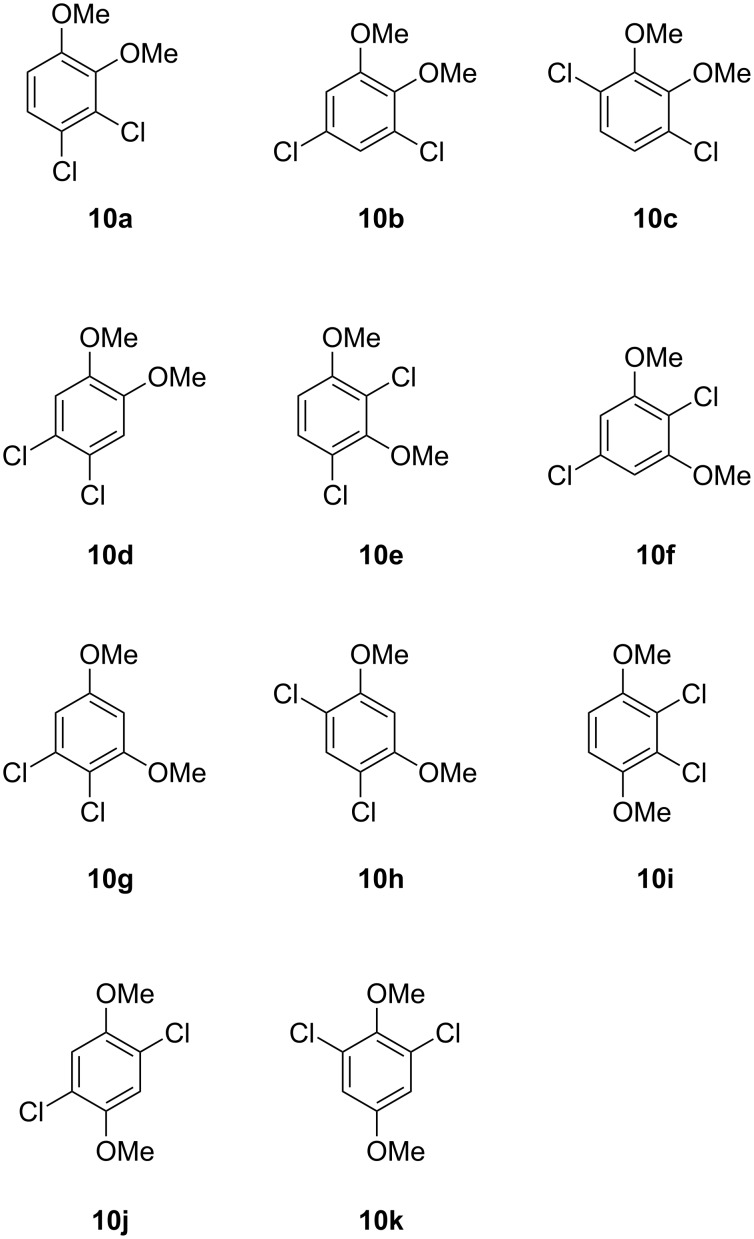
Constitutional isomers of dichlorodimethoxybenzene as candidate structures for **Y**.

**Scheme 2:**
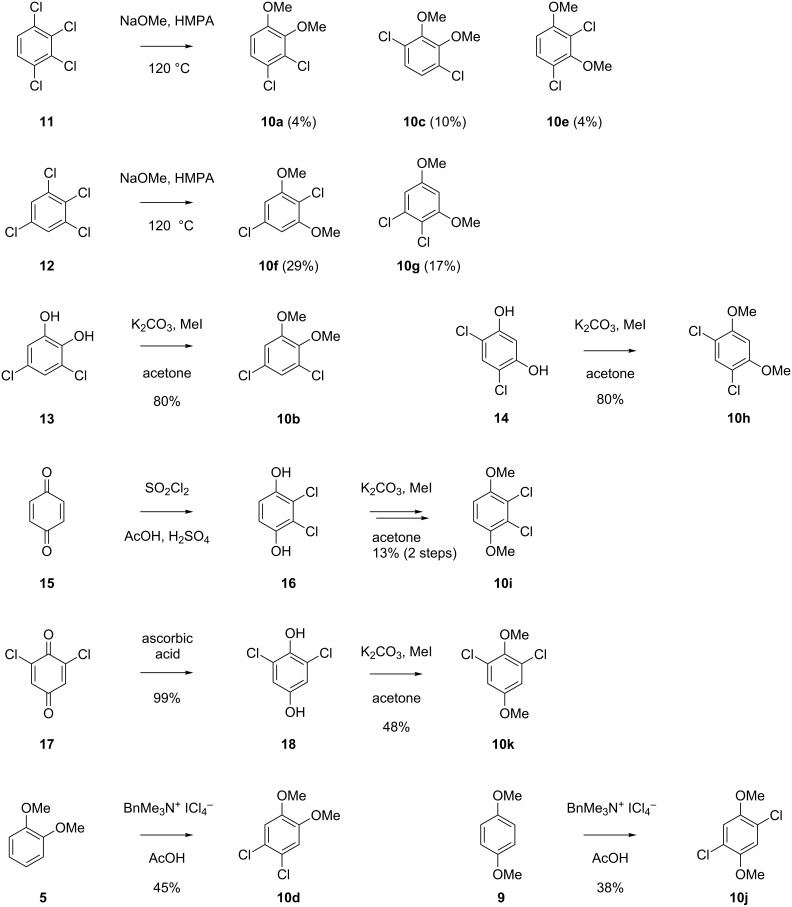
Synthesis of chlorodimethoxybenzenes as reference compounds for **Y**.

For compound **Y** a series of eleven constitutional isomers as shown in Figure 4 were possible. All eleven compounds were obtained by synthesis as summarised in Scheme 2. The compounds **10a**, **10c**, and **10e** were obtained by treatment of 1,2,3,4-tetrachlorobenzene (**11**) with sodium methoxide in hot HMPA. This reaction is reported to yield mainly 1,2,3-trichloro-4-methoxybenzene and 1,2,4-trichloro-3-methoxybenzene by monosubstitution [23], but small amounts of disubstitution products were also formed that were rigorously purified from the mixture by repeated column chromatography. Starting from 1,2,3,5-tetrachlorobenzene (**12**) the same reaction conditions also provided a mixture of mono- and disubstitution products, but the target compounds **10f** and **10g** were obtained in better yields (29% and 17%). Derivative **10b** was prepared from 3,5-dichlorocatechol (**13**) by methylation with potassium carbonate and methyl iodide in high yield (80%). Accordingly, methylation of 4,6-dichlororesorcinol (**14**) provided **10h** in 80% yield, and methylation of 2,3-dichlorohydroquinone (**16**), prepared from benzoquinone (**15**) by treatment with SO_2_Cl_2_ under acidic conditions, generated **10i** (13% via two steps). Reduction of 2,6-dichlorobenzoquinone with ascorbic acid to the corresponding hydroquinone **18** followed by methylation yielded 48% of **10k**. Chlorination of veratrole (**5**) with two equivalents of benzyltrimethylammonium tetrachloroiodate resulted in **10d** (45%), while the same procedure starting from 1,4-dimethoxybenzene gave access to **10j** (38%). The NMR spectroscopic data and results of GC–MS analyses for all eleven dichlorodimethoxybenzenes in comparison to the volatile **Y** are summarised in Table 3. All mass spectra, ^1^H and ^13^C NMR spectra are shown in Supporting Information File 1. Comparison of mass spectra and retention indices unequivocally established the identity of **Y** and **10b** (Table 3).

**Table 3:** NMR spectroscopic data and GC retention indices of all constitutional isomers of dichlorodimethoxybenzene in comparison to natural **Y**.

Compound	^1^H NMR	^13^C NMR	*I* (HP5-MS)

**10c**	7.07 (s, 2H, 2 × CH) 3.92 (s, 6H, 2 × OCH_3_)	150.7 (2 × C_q_) 127.1 (2 × C_q_) 125.1 (2 × CH) 61.1 (2 × OCH_3_)	1339
**10b**	6.98 (d, 1H, ^4^*J*_H,H_ = 2.5 Hz, CH) 6.80 (d, 1H, ^4^*J*_H,H_ = 2.5 Hz, CH) 3.86 (s, 3H, OCH_3_) 3.84 (s, 3H, OCH_3_)	154.1 (C_q_) 144.4 (C_q_) 129.1 (C_q_) 128.9 (C_q_) 121.6 (CH) 111.7 (CH) 60.8 (OCH_3_) 56.3 (OCH_3_)	1426
**10k**	6.84 (s, 2H, 2 × CH) 3.84 (s, 3H, OCH_3_) 3.76 (s, 3H, OCH_3_)	155.7 (C_q_) 146.2 (C_q_) 129.5 (2 × C_q_) 114.5 (2 × CH) 60.8 (OCH_3_) 55.9 (OCH_3_)	1443
**10j**	6.97 (s, 2H, 2 × CH) 3.85 (s, 6H, 2 × OCH_3_)	149.2 (2 × C_q_) 120.9 (2 × C_q_) 114.5 (2 × CH) 56.8 (2 × OCH_3_)	1448
**10a**	7.15 (d, 1H, ^3^*J*_H,H_ = 9.0 Hz, CH) 6.76 (d, 1H, ^3^*J*_H,H_ = 9.0 Hz, CH) 3.86 (s, 3H, OCH_3_) 3.85 (s, 3H, OCH_3_)	152.4 (Cq) 146.7 (Cq) 127.5 (Cq) 124.7 (CH) 124.5 (Cq) 111.0 (CH) 60.6 (OCH_3_) 56.2 (OCH_3_)	1466
**10e**	7.23 (d, 1H, ^3^*J*_H,H_ = 9.0 Hz, CH) 6.66 (d, 1H, ^3^*J*_H,H_ = 9.0 Hz, CH) 3.90 (s, 3H, OCH_3_) 3.89 (s, 3H, OCH_3_)	155.1 (C_q_) 153.2 (C_q_) 127.7 (CH) 120.7 (C_q_) 118.2 (C_q_) 107.9 (CH) 60.6 (OCH_3_) 56.5 (OCH_3_)	1487
**10d**	6.90 (s, 2H, 2 × CH) 3.86 (s, 6H, 2 × OCH_3_)	148.3 (2 × C_q_) 123.4 (2 × C_q_) 112.9 (2 × CH) 56.2 (2 × OCH_3_)	1505
**10h**	7.34 (s, 1H, CH) 6.53 (s, 1H, CH) 3.90 (s, 6H, 2 × CH_3_)	154.5 (2 × C_q_) 130.5 (CH) 114.0 (2 × C_q_) 97.8 (CH) 56.5 (2 × OCH_3_)	1545
**10f**	6.60 (s, 2H, 2 × CH) 3.89 (s, 6H, 2 × CH_3_)	156.4 (2 × C_q_) 133.0 (C_q_) 109.3 (C_q_) 105.5 (2 × CH) 56.5 (2 × OCH_3_)	1556
**10g**	6.62 (d, 1H, ^4^*J*_H,H_ = 2.7 Hz, CH) 6.42 (d, 1H, ^4^*J*_H,H_ = 2.7 Hz, CH) 3.87 (s, 3H, OCH_3_) 3.79 (s, 3H, OCH_3_)	158.7 (C_q_) 156.7 (C_q_) 133.8 (C_q_) 113.5 (C_q_) 106.4 (CH) 98.6 (CH) 56.4 (OCH_3_) 55.7 (OCH_3_)	1565
**10i**	6.81 (s, 2H, 2 × CH) 3.86 (s, 6H, 2 × OCH_3_)	150.3 (2 × C_q_) 123.1 (2 × C_q_) 110.0 (2 × CH) 56.8 (2 × OCH_3_)	1574
**Y**			1426

**Figure 5:**
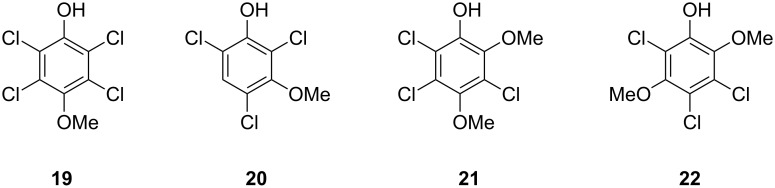
Known natural products that are structurally related to **4b** and **10b** from *Geniculosporium*.

Both compounds **4b** and **10b** that were identified in this study as volatiles emitted by *Geniculosporium* have not been reported as natural products before. However, the structurally related compounds **4f** and **10k** are known from the fungus *Bjerkandera adusta* [24]. It is interesting to note that in both cases the dichlorodimethoxybenzene (**10b** or **10k**) can arise by a second chlorination from the monochlorodimethoxybenzene (**4b** or **4f**, respectively), suggesting that in both organisms the sets of chlorinated compounds arise via one and the same biosynthetic pathway. Known structurally related compounds from fungi are drosophilin A (**19**) [25] or the trichlorinated phenols **20**–**22** (Figure 5) [26]. The biological function of **4b** and **10b** in *Geniculosporium* is unknown, but the antibiotic activity of the related compound drosophilin A towards bacteria is well known [27].

### A iodinated volatile from *Streptomyces chartreusis*

**Figure 6:**
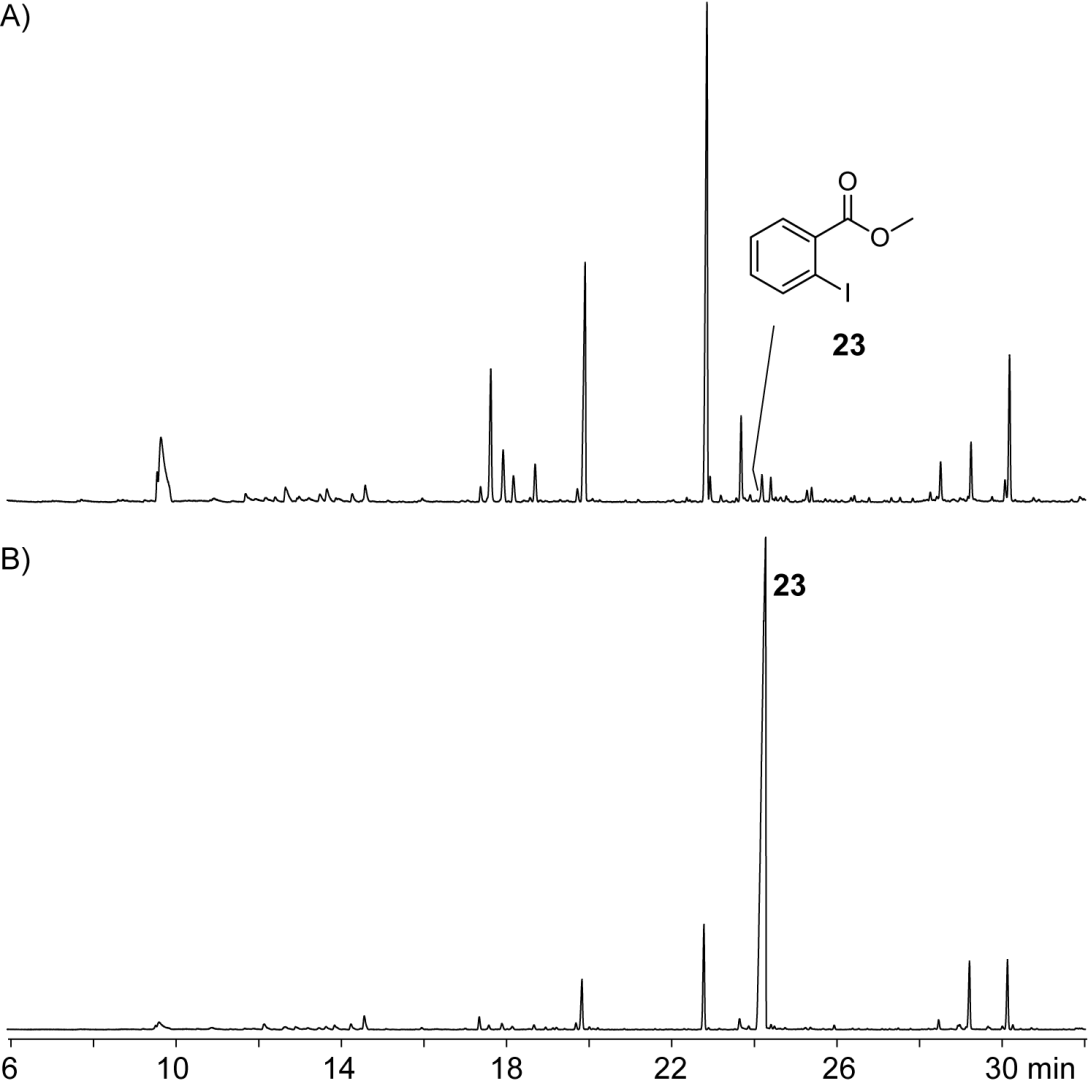
Total ion chromatograms of headspace extracts from *S. chartreusis*. A) Growth on 84 GYM showing production of **23** as a trace compound. B) Growth on 84 GYM amended with 2-iodobenzoic acid (1 mM) results in the emission of **23** as the principal volatile compound.

During the course of our work on volatiles from various bacteria and fungi we rarely encountered chlorinated volatiles. Brominated and iodinated volatiles are even more rare. Investigations on the volatiles released by the actinomycete *Streptomyces chartreusis* during growth on 84 GYM medium, also collected by use of a CLSA followed by GC–MS analysis of the obtained headspace extracts, led to a surprising result. The extracts contained a trace compound that was readily identified from its mass spectrum and comparison to an authentic standard as methyl 2-iodobenzoate (**23**, Figure 6A). This compound was observed as the principal compound in headspace extracts during growth of *S. chartreusis* on 84 GYM supplemented with 2-iodobenzoic acid suggesting that **23** arises by efficient methylation of the carboxylic acid (Figure 6B). 2-Iodobenzoic acid is frequently used in many chemical laboratories including ours, e.g., as a starting material for the preparation of the oxidising agents IBX and DMP. A contamination of a bacterial agar plate with traces of 2-iodobenzoic acid was assumed as a possible, albeit unlikely explanation for the formation of **23** in the bacterial cultures. In our laboratories all analytical work is strictly spatially separated from the other chemical (synthetic) work. Furthermore, a contamination with 2-iodobenzoic acid as the reason for the emission of **23** by *S. chartreusis* was excluded by the sixfold repetition of the headspace analyses during a time period of almost two years (January 2012–August 2013), using new batches of medium in all cases. Compound **23** was repeatedly detected in all six analyses which clearly supports the natural origin of this volatile.

**Figure 7:**
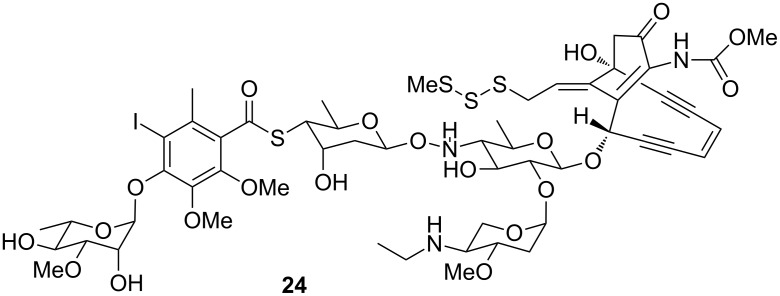
Calicheamicin, a known iodinated compound from the actinomycete *Micromonspora echinospora*.

The iodinated compound **23** has never been reported as a natural product. One of the few reported iodinated compounds is the structurally highly complex calicheamicin **24** (Figure 7) from *Micromonospora echinospora* [28] that also contains an iodinated aromatic subunit.

## Conclusion

In summary, we have presented the identification of 4-chloro-1,2-dimethoxybenzene (**4b**) and 1,5-dichloro-2,3-dimethoxybenzene (**10b**) as volatile natural products released by the fungus *Geniculosporium*. Furthermore, *Streptomyces chartreusis* was shown to emit methyl 2-iodobenzoate (**23**). All three compounds are new natural products. Future work on the biological function of these metabolites and the biosynthetic pathways is now possible.

## Experimental

**Strains and culture conditions:** *Geniculosporium* sp. 9910 was obtained from Barbara Schulz (Braunschweig) and grown on potato–carrot medium [29] on petri dishes and incubated for 21 d at 28 °C prior to analysis. *Streptomyces chartreusis* NRRL 3882 (= DSM 41447) was obtained from DSMZ (Braunschweig, Germany) and grown on 84 GYM (20.0 g rolled oats, boiled in 1 L of distilled water for 20 min, then oats were filtered off, trace element solution (1 mL) containing 1 g L^−1^ FeSO_4_·7H_2_O, 1 g L^−1^ MnCl_4_·4H_2_O, and 1 g L^−1^ ZnSO_4_·7H_2_O was added, followed by sterilisation at 121 °C for 20 min).

**Headspace analyses:** The volatiles released by *Geniculosporium* or *Streptomyces chartreusis* were trapped by use of the CLSA (closed-loop stripping analysis) technique as described previously [30]. GC–MS analyses of the obtained headspace extracts were carried out on an Agilent 7890A connected with an Agilent 5975C inert mass detector fitted with a HP5-MS fused silica capillary column (30 m, 0.25 mm i. d., 0.25 μm film, Agilent). GC conditions were as follows: inlet pressure 77.1 kPa, He 23.3 mL min^−1^, injection volume 1.5 μL, transfer line 300 °C, electron energy 70 eV. The operation mode was splitless (60 s valve time) and the carrier gas was He at 1.2 mL min^−1^. The GC was programmed as follows: 5 min at 50 °C increasing with 5 °C min^−1^ to 320 °C. Retention indices (*I*) were determined from a homologous series of *n*-alkanes (C_8_–C_38_). The mass spectra of the natural compounds **X** and **Y** are shown in Figure 2. For comparison the mass spectra of all commercially available and synthetic chlorodimethoxybenzenes **4a**–**4f** and dichlorodimethoxybenzenes **10a**–**10k** are shown in Figures S1 and S2 of Supporting Information File 1. Retention indices of natural **X** and **Y** and of all chlorodimethoxybenzenes **4a**–**4f** and dichlorodimethoxybenzenes **10a**–**10k** are listed in Table 1 and Table 2.

## Supporting Information

File 1 Synthetic procedures, characterization data, mass spectra of all isomers of chlorodimethoxybenzene and dichlorodimethoxybenzene and ^1^H, ^13^C, and DEPT spectra of all synthetic compounds.
