# Supplementary material for: Halogenated volatiles from the fungus Geniculosporium and the actinomycete Streptomyces chartreusis
Source: Beilstein J Org Chem. 2013 Dec 3;9:2767–77. doi: 10.3762/bjoc.9.311 (PMC3869313; doi:10.3762/bjoc.9.311)
Supplement: File 1 — Synthetic procedures, characterization data, mass spectra of all isomers of chlorodimethoxybenzene and dichlorodimethoxybenzene and 1H, 13C, and DEPT spectra of all synthetic compounds. [file Beilstein_J_Org_Chem-09-2767-s001.pdf]

Supporting Information

for

Halogenated volatiles from the fungus *Geniculosporium* and the actinomycete *Streptomyces chartreusis*

Tao Wang[1], Patrick Rabe[1], Christian A. Citron[1] and Jeroen S. Dickschat*[1]

Address: [1]Institut für Organische Chemie, TU Braunschweig, Hagenring 30, 38106 Braunschweig, Germany

Email: Jeroen S. Dickschat - j.dickschat@tu-bs.de

* Corresponding author

Synthetic procedures, characterization data, mass spectra of all isomers of chlorodimethoxybenzene and dichlorodimethoxybenzene and ^{1}H, ^{13}C, and DEPT spectra of all synthetic compounds

Synthetic procedures

General methods: Chemicals were purchased from Acros Organics (Geel, Belgium) or Sigma Aldrich Chemie GmbH (Steinheim, Germany). All non-aqueous reactions were performed under an inert atmosphere (N_2) in flame-dried flasks. Solvents were purified by distillation and dried according to standard methods. For general procedures, relative quantities of reagents are given in equivalents (equiv), and the amounts of solvents are indicated by the final concentrations of the starting material (set to 1.0 equiv). Thin-layer chromatography was performed with 0.2 mm precoated plastic sheets Polygram® Sil G/UV254 (Machery-Nagel). Column chromatography was carried out using Merck silica gel 60 (70–200 mesh). ^{1}H NMR and ^{13}C NMR spectra were recorded on Bruker DRX-400 (400 MHz) and AV III-400 (400 MHz) spectrometers, and were referenced against TMS (δ = 0.00 ppm) for ^{1}H NMR and $CDCl_3$ (δ = 77.01 ppm) for ^{13}C NMR. NMR data of all commercially available and synthetic chlorodimethoxybenzenes **4a**–**4f** and dichlorodimethoxybenzenes **10a**–**10k** are listed in Tables 2 and 3 (main text). ^{1}H NMR, ^{13}C NMR, and DEPT spectra of synthetic compounds are shown in Figures S3–S53. UV spectra were obtained using a Varian Cary 100 Bio, and IR spectra were recorded with a Bruker Tensor 27 ATR.

General procedure for the methylation of phenols: Similar to the procedure by An et al. [1], to a solution of the catechol (**13**), resorcinol (**8** or **14**), or hydroquinone (crude **16**, cf. below, or **18**) (1.0 equiv) in acetone (0.1 M), potassium carbonate (5.0 equiv) was added and the mixture was stirred for 10 min. Methyl iodide (2.6 equiv) was added and the reaction mixture was stirred under reflux for 16 h. After cooling to room temperature water was added and the mixture was extracted three times with diethyl ether. The combined organic extracts were dried over $MgSO_4$ and concentrated in vacuo. The residue was purified by column chromatography on silica gel to afford the pure compounds **4c** (from **8**), **10b** (from **13**), **10h** (from **14**), **10i** (from crude **16**), and **10k** (from **18**).

2-Chloro-1,3-dimethoxybenzene (**4c**): Pale yellow solid (76 mg, 0.44 mmol, 88%). TLC (hexane/ethyl acetate 10:1) R_f 0.38; IR (ATR) $\tilde{\nu}$ 3011 (w), 2966 (w), 2947 (w), 2840 (w), 1594 (m), 1472 (m), 1435 (m), 1299 (m), 1253 (m), 1191 (w), 1174 (w), 1099 (m), 1053 (m), 1025 (m), 849 (w), 764 (m), 709 (m), 654 (m), 597 (m); UV–vis λ_{max} (log ε) 280 (3.08), 274 (3.10), 230 (3.79) nm.

1,5-Dichloro-2,3-dimethoxybenzene (**10b**): Colourless solid (50 mg, 0.24 mmol, 80%). TLC (hexane/ethyl acetate 10:1) R_f 0.55; IR (ATR) $\tilde{\nu}$ 3089 cm^{-1} (w), 3005 (w), 2967 (w), 2939 (w), 2831 (w), 1572 (m), 1480 (m), 1425 (m), 1399 (m), 1292 (m), 1263 (m), 1229 (m), 1171 (m), 1102 (m), 1043 (m), 999 (m), 895 (m), 830 (m), 760 (m), 718 (m), 589 (m); UV–vis (CH_2Cl_2) λ_{max} (log ε) 286 (3.22), 280 (3.20), 231 (3.83) nm.

1,5-Dichloro-2,4-dimethoxybenzene (**10h**): Pale yellow solid (49 mg, 0.24 mmol, 80%). TLC (hexane/ethyl acetate 10:1) R_f 0.37; IR (ATR) $\tilde{\nu}$ 2976 (w), 2946 (w), 2879 (w), 2847 (w), 1575 (m), 1494 (m), 1470 (m), 1455 (m), 1428 (m), 1373 (m), 1294

(m), 1231 (m), 1207 (m), 1172 (m), 1087 (m), 1055 (m), 1020 (m), 860 (m), 803 (m), 741 (m), 579 (m) cm^{-1}; UV–vis (CH_2Cl_2) λ_{max} (log ε) 292 (3.59), 233 (3.89) nm.

2,3-Dichloro-1,4-dimethoxybenzene (**10i**): Pale yellow solid (1.03 g, 5.0 mmol, 13% over two steps from **15**). TLC (hexane/ethyl acetate 10:1) R_f 0.42; IR (ATR) $\tilde{\nu}$ 3094 (w), 2967 (w), 2946 (w), 2914 (w), 2873 (w), 2840 (w), 1591 (w), 1570 (w), 1479 (m), 1457 (m), 1406 (w), 1303 (w),1262 (m), 1192 (w), 1116 (w), 1038 (s), 900 (w), 790 (s), 711 (w), 608 (m) cm^{-1}; UV–vis (CH_2Cl_2) λ_{max} (log ε) 296 (3.59), 229 (3.76) nm.

1,3-Dichloro-2,5-dimethoxybenzene (**10k**): Colourless solid (49 mg, 0.24 mmol, 48%). TLC (hexane/ethyl acetate 10:1) R_f 0.56; IR (ATR) $\tilde{\nu}$ 3087 (w), 2992 (w), 2949 (w), 2903 (w), 2835 (w), 1704 (w), 1663(w), 1610 (m), 1594 (m), 1559 (m), 1480 (s), 1420 (m), 1403 (m), 1306 (m), 1257 (m), 1222 (s), 1175 (m), 1074 (m), 1042 (s), 984 (s), 909 (w), 909 (m), 850 (m), 831 (m), 804 (s), 761 (s), 717 (m), 606 (m) cm^{-1}; UV–vis (CH_2Cl_2) λ_{max} (log ε) 292 (3.56), 287 (3.45), 230 (3.78) nm.

1-Chloro-2,3-dimethoxybenzene (4a): To a solution of veratrole (**5**) (1.38 g, 1.00 mmol, 1.0 equiv) in dry ether (2.00 mL), 1.6 M *n*-butyllithium in hexane (1.25 mL) was added slowly and the mixture was stirred at room temperature for 48 h [2]. The resulting solution of (2,3-dimethoxyphenyl)lithium was diluted with dry diethyl ether (12 mL), triethylamine (101 mg, 1.00 mmol, 1.0 equiv) was added, and the mixture was further stirred for 10 min. Trifluoromethanesulfonyl chloride (169 mg, 1.00 mmol, 1.0 equiv) was added dropwise to the mixture [3]. After stirring at room temperature for 1 h, the reaction was quenched by the addition of water. The aqueous phase was extracted three times with diethyl ether. The combined extracts were dried over $MgSO_4$, concentrated in vacuo and purified by column chromatography on silica gel to afford 1-chloro-2,3-dimethoxybenzene (**4a**, 80 mg, 0.47 mmol, 47%) as a pale yellow oil. TLC (hexane/ethyl acetate 10:1) R_f 0.40; IR (ATR) $\tilde{\nu}$ 3003 (w), 2936 (w), 2835 (w), 1582 (w), 1481 (m), 1460 (m), 1427 (m), 1295 (w), 1263 (m), 1237 (m), 1173 (w), 2253 (w), 1079 (w), 1041 (s), 1001 (s), 855 (m), 799 (w), 771 (m), 736 (m), 654 (w), 556 (w) cm^{-1}; UV–vis (CH_2Cl_2) λ_{max} (log ε) 280 (3.15), 273 (3.17), 231 (3.55) nm.

4-Chloro-1,2-dimethoxybenzene (4b): To a cooled solution (0 °C) of 3,4-dimethoxyaniline (**6**) (3.10 g, 20.0 mmol, 1.0 equiv) in 6 M HCl (30 mL) was added a 2.5 M solution of $NaNO_2$ (1.40 g, 20.0 mmol, 1.0 equiv) in H_2O (8 mL), resulting in solution A. In a separate flask $CuSO_4$ (5.80 g, 26.7 mmol, 1.3 equiv) was dissolved in H_2O (25 mL) and NaCl (2.30 g, 40.0 mmol, 2.0 equiv) was added. To this solution was added Na_2SO_3 (1.70 g, 13.4 mmol, 0.7 equiv) in H_2O (6 mL). The precipitate was filtered off, washed with H_2O, dissolved in concentrated HCl (11 mL) and cooled to 0 °C. Solution A was added dropwise and the mixture was stirred for 1 h at room temperature, followed by 30 min at 100 °C. After cooling to room temperature the mixture was extracted three times with EtOAc. The combined extracts were dried over $MgSO_4$ and concentrated under reduced pressure. The residue was purified by flash column chromatography on silica gel yielding **4b** (2.10 g, 12.20 mmol, 61%) as colourless oil. TLC (hexane/ethyl acetate 10:1) R_f 0.22; IR (ATR) $\tilde{\nu}$ 3003 (w), 2956

(w), 2908 (w), 1591 (m), 1500 (s), 1441 (m), 1402 (w), 1252 (s), 1226 (s), 1177 (m), 1131 (m), 1022 (s), 873 (m), 838 (m), 797 (m), 763 (m), 643 (m) cm^{-1}; UV–vis (CH_2Cl_2) λ_{max} (log ε) 283 (3.47), 235 (3.91) nm.

General procedure for *ipso*-substitutions of chlorobenzenes to methoxybenzenes: According to the method of Testaferri et al. [4], sodium methoxide (4.0 equiv) was added to a stirred solution of the tetrachlorobenzene **11** or **12** (1.0 equiv) in hexamethylphosphoramide (0.3 M). The reaction mixture was stirred at 120 °C for 3 h and then cooled to room temperature. At this stage the mixture contains some minor amounts of phenols that are subsequently methylated by the addition of methyl iodide (1.5 equiv). The mixture was stirred for 1 h at room temperature and then poured into 2 M HCl. The aqueous phase was extracted three times with diethyl ether. The combined organic layers were dried over $MgSO_4$ and concentrated in vacuo. Column chromatography on silica gel yielded the target compounds **10a**, **10c**, and **10e** (from **11**), and **10f** and **10g** (from **12**).

1,2-Dichloro-3,4-dimethoxybenzene (**10a**), **1,4-dichloro-2,3-dimethoxybenzene** (**10c**), and **1,3-dichloro-2,4-dimethoxybenzene** (**10e**): The crude product contained mainly monosubstitution products, but small amounts of disubstitution products could be isolated by rigorous purification via column chromatography. The yields were: **10a** (40 mg, 0.19 mmol, 4%, pale yellow oil), **10c** (100 mg, 0.48 mmol, 10%, colourless solid), and **10e** (40 mg, 0.19 mmol, 4%, colourless oil).

10a: TLC (hexane/ethyl acetate 5:1) R_f 0.25; IR (ATR) $\tilde{\nu}$ 3005 (w), 2939 (w), 2840 (w), 1579 (w), 1474 (m), 1431 (m), 1400 (m), 1291 (m), 1262 (m), 1169 (w), 1140 (w), 1043 (m), 1007 (m), 889 (w), 834 (m), 796 (m), 752 (w), 672 (m), 644 (w), 598 (w) cm^{-1}; UV–vis (CH_2Cl_2) λ_{max} (log ε) 288 (3.23), 283 (3.22), 230 (3.84).

10c: TLC (hexane/ethyl acetate 5:1) R_f 0.47; IR (ATR) $\tilde{\nu}$ 3003 (w), 2973 (w), 2941 (w), 2876 (w), 1579 (w), 1459 (m), 1430 (m), 1403 (m), 1239 (m), 1152 (w), 1128 (m), 1004 (s), 865 (m), 797 (m), 645 (m), 627 (m) cm^{-1}; UV–vis (CH_2Cl_2) λ_{max} (log ε) 274 (2.60), 231 (3.87) nm.

10e: TLC (hexane/ethyl acetate 5:1) R_f 0.56; IR (ATR) $\tilde{\nu}$ 3007 (w), 2968 (w), 2941 (w), 2873 (w), 2840 (w), 1579 (w), 1466 (m), 1433 (m), 1402 (m), 1294 (m), 1270 (w), 1227 (m), 1151 (w), 1081 (s), 1008 (m), 914 (m), 794 (s), 750 (m), 686 (m), 644 (w), 576 (w) cm^{-1}; UV–vis (CH_2Cl_2) λ_{max} (log ε) 288 (3.22), 283 (3.21), 230 (3.84).

2,5-Dichloro-1,3-dimethoxybenzene (**10f**) and **1,2-dichloro-3,5-dimethoxybenzene** (**10g**): The yields were: **10f** (120 mg, 0.58 mmol, 29%, colourless solid) and **10g** (72 mg, 0.35 mmol, 17%, colourless solid).

10f: TLC (hexane/ethyl acetate 10:1) R_f 0.27; IR (ATR) $\tilde{\nu}$ 3092 (w), 3032 (w), 2976 (w), 2942 (w), 2909 (w), 2839 (w), 1590 (m), 1567 (m), 1459 (m), 1439 (m), 1404 (m), 1315 (m), 1296 (m), 1230 (m), 1119 (m), 1061 (m), 914 (m), 869 (m), 820 (m), 669 (s), 633 (m), 582 (m) cm^{-1}; UV–vis (CH_2Cl_2) λ_{max} (log ε) 276 (3.01), 231 (3.90) nm.

10g: TLC (hexane/ethyl acetate 10:1) R_f 0.45; IR (ATR) $\tilde{\nu}$ 3092 (w), 2983 (w), 2954 (w), 2939 (w), 2837 (w), 1594 (m), 1569 (m), 1463 (m), 1429 (m), 1410 (m), 1323 (m), 1274 (m), 1214 (m), 1185 (m), 1159 (m), 1095 (m), 1030 (m), 933 (m), 857 (m), 825 (m), 782 (m), 697 (s), 658 (m), 639 (m), 621 (m); UV–vis (CH_2Cl_2) λ_{max} (log ε) 289 (3.42), 229 (3.91) nm.

2-Chloro-1,4-dimethoxybenzene (4f): Following the procedure of Kajigaeshi et al. [5], a solution of 1,4-dimethoxybenzene (**9**, 414 mg, 3.00 mmol, 1.0 equiv) in CH_2Cl_2 was treated with $Bn(Me)_3N^+ICl_4^-$ (1.30 g, 3.00 mmol, 1.0 equiv) and stirred overnight at room temperature. The reaction mixture was diluted with H_2O and extracted three times with EtOAc. The combined organic layers were dried over $MgSO_4$ and concentrated under reduced pressure. The residue was purified by flash column chromatography on silica gel yielding **4f** (174 mg, 1.00 mmol, 34%) as yellow oil. TLC (hexane/ethyl acetate 20:1) R_f 0.31; IR (ATR) $\tilde{\nu}$ 3003 (w), 2948 (w), 2836 (w), 1581 (w), 1496 (s), 1461 (m), 1438 (m), 1271 (m), 1213 (s), 1180 (m), 1039 (s), 882 (m), 797 (m), 735 (s) cm^{-1}; UV–vis (CH_2Cl_2) λ_{max} (log ε) 294 (3.56), 230 (3.78) nm.

General procedure for the bis-chlorination of dimethoxybenzenes: A solution of the dimethoxybenzene **5** or **9** (1.0 equiv) in acetic acid (0.3 M) was treated with $Bn(Me)_3N^+ICl_4^-$ (2.0 equiv) [5]. The mixture was stirred for 1 h at room temperature, diluted with H_2O, and extracted three times with EtOAc. The combined extracts were dried over $MgSO_4$ and concentrated under reduced pressure. The residue was purified by flash column chromatography on silica gel to yield **10d** (from **5**) and **10j** (from **9**).

1,2-Dichloro-4,5-dimethoxybenzene (**10d**): Pale yellow oil (466 mg, 2.25 mmol, 45%). TLC (hexane/ethyl acetate 10:1) R_f 0.39; IR (ATR) $\tilde{\nu}$ 3004 (w), 2966 (w), 2906 (w), 2839 (w), 1594 (m), 1503 (s), 1432 (s), 1362 (m), 1337 (m), 1253 (s), 1210 (s), 1178 (s), 1131 (s), 1025 (s), 919 (s), 838 (s), 793 (s), 676 (s) cm^{-1}; UV–vis (CH_2Cl_2) λ_{max} (log ε) 290 (3.51), 235 (3.91) nm.

1,4-Dichloro-2,5-dimethoxybenzene (**10j**): Colourless oil (118 mg, 0.57 mmol, 38%). TLC (hexane/ethyl acetate 20:1) R_f 0.30; IR (ATR) $\tilde{\nu}$ 3029 (w), 2909 (w), 1501 (s), 1481 (m), 1439 (s), 1367 (m), 1279 (m), 1213 (s), 1187 (m), 1079 (s), 1025 (s), 858 (s), 775 (s) cm^{-1}; UV–vis (CH_2Cl_2) λ_{max} (log ε) 299 (3.69), 230 (3.90) nm.

2,3-Dichlorohydroquinone (16): To a solution of 1,4-benzoquinone (**15**, 4.32 g, 40.0 mmol, 1.0 equiv) in dry ether (35 mL), sulfuryl chloride (6.50 mL, 80.0 mmol, 2.0 equiv) was added and stirred for 16 h at room temperature. The mixture was cooled with ice and the dark brown precipitate was filtered off, washed with cold diethyl ether (10 mL), and dried in vacuo. A suspension of the obtained solid was treated with glacial AcOH (20 mL) and concentrated H_2SO_4 (2 mL). The mixture was stirred for 24 h at 60 °C and then poured on ice, followed by extraction (3×) with diethyl ether. The collected organic layers were washed with brine, dried over $MgSO_4$ and concentrated to yield a brown solid (3.10 g) containing **16** (GC–MS) that was used in the methylation step (cf. above) without further purification.

2,6-Dichlorobenzene-1,4-diol (18): To a solution of 2,6-dichloro-*p*-benzoquinone (**17**, 1.00 g, 5.70 mmol) in ethyl acetate (20 mL), phosphate buffer (10 mL, 10 mmol Na_2HPO_4, 10 mmol KH_2PO_4, pH 7.0) and L-ascorbic acid (4.0 g) were added. The mixture was shaken in a separatory funnel for 5 min. The mixture was extracted three times with ethyl acetate, the combined extracts were dried over $MgSO_4$ and concentrated in vacuo to yield pure 2,6-dichlorobenzene-1,4-diol (**18**, 1.00 g, 5.60 mmol, 99%) as a colourless solid [6]. TLC (hexane/ethyl acetate 10:1) R_f 0.56. ^{1}H NMR (400 MHz, $CDCl_3$) δ 6.72 (s, 2H, 2 × CH), 4.86 (s, 2H, 2 × OH) ppm. ^{13}C NMR (100 MHz, $CDCl_3$) δ 151.9 (C_q), 143.5 (C_q), 123.9 (2 × C_q), 116.4 (2 × CH) ppm; IR (ATR) $\tilde{\nu}$ 3384 cm^{-1} (br), 3075 (w), 2517 (m), 2415 (m), 2077 (w), 1791 (m), 1746 (w), 1687 (w), 1614 (w), 1577 (m), 1477 (s), 1430 (m), 1344 (m), 1275 (w), 1213 (m), 1112 (m), 1095 (m), 971 (s), 947 (s), 844 (m), 803 (s), 700 (w), 599 (w) cm^{-1}; UV–vis (CH_2Cl_2) λ_{max} (log ε) 296 (3.58), 229 (3.49) nm. MS (EI, 70 eV) *m*/*z* (%) 178 (100) $[M]^+$, 142 (12), 114 (59), 86 (41), 79 (44), 61 (16), 53 (48).

References

1. An, P.; Shi, Z.-F.; Dou, W.; Cao, X.-P.; Zhang, H.-L. *Org. Lett.*, **2010,** *12,* 4364–4367.
2. Bunce, N. J.; Stephenson, K. L. *Can. J. Chem.*, **1989,** *67,* 220–226.
3. Hakimelahi, G. H.; Just, G. *Tetrahedron Lett.*, **1979,** *20,* 3643–3644.
4. Testaferri, L.; Tiecco, M.; Tingoli, M.; Chianelli, D.; Montanucci, M. *Tetrahedron,* **1983,** *39,* 193–197.
5. Kajigaeshi, S.; Shinmasu, Y.; Fujisaki, S.; Kakinami, T. *Chem. Lett.,* **1989,** 415–418.
6. Kiyohara, H.; Hatta, T.; Ogawa, Y.; Kakuda, T.; Yokohama, H.; Takizawa, N. *Appl. Environ. Microbiol.*, **1992,** *58,* 1276–1283.

Mass spectra of chlorodimethoxybenzenes and dichloro-dimethoxybenzenes

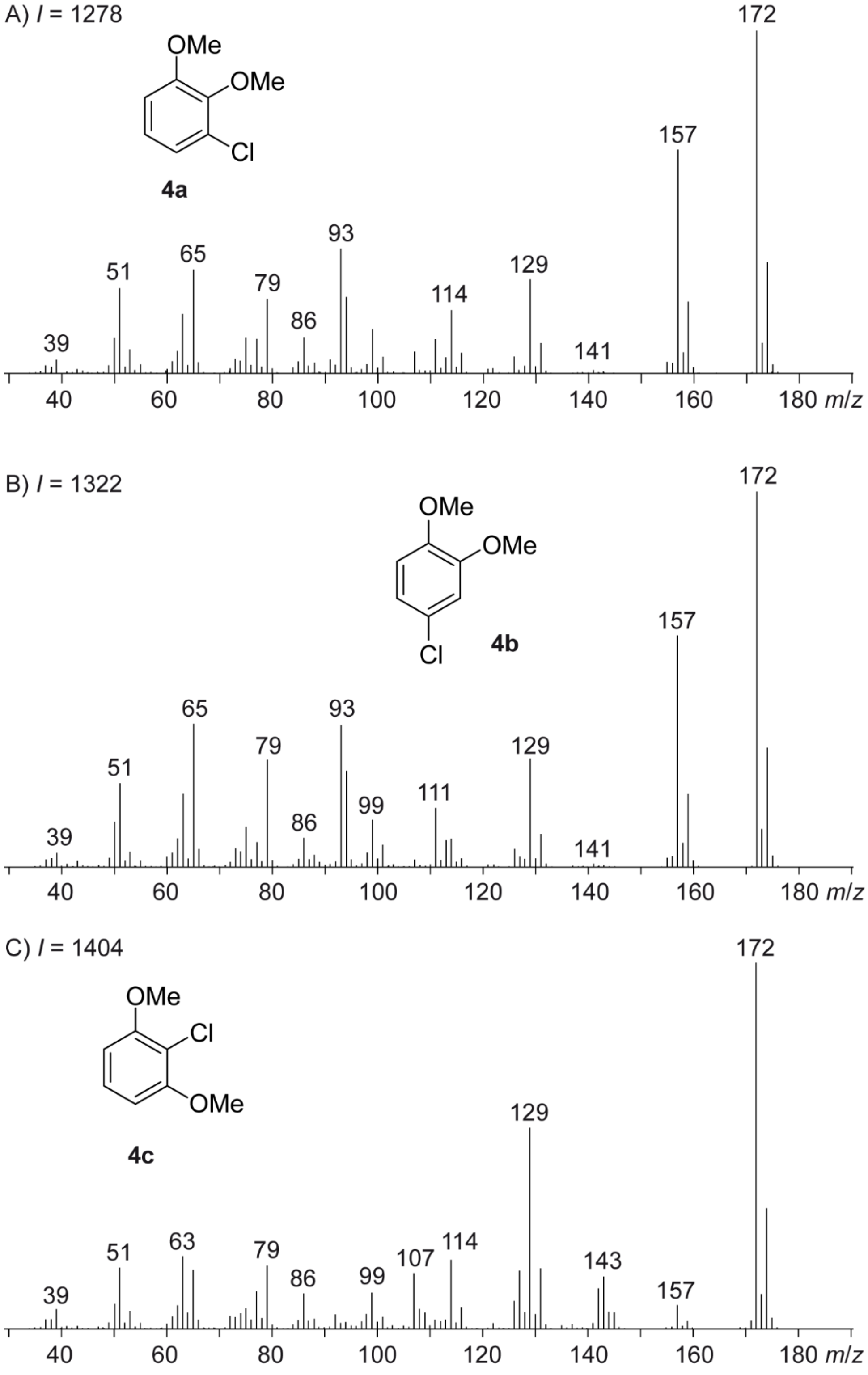

Figure 1: Mass spectra of chlorodimethoxybenzenes **4a–4c**.

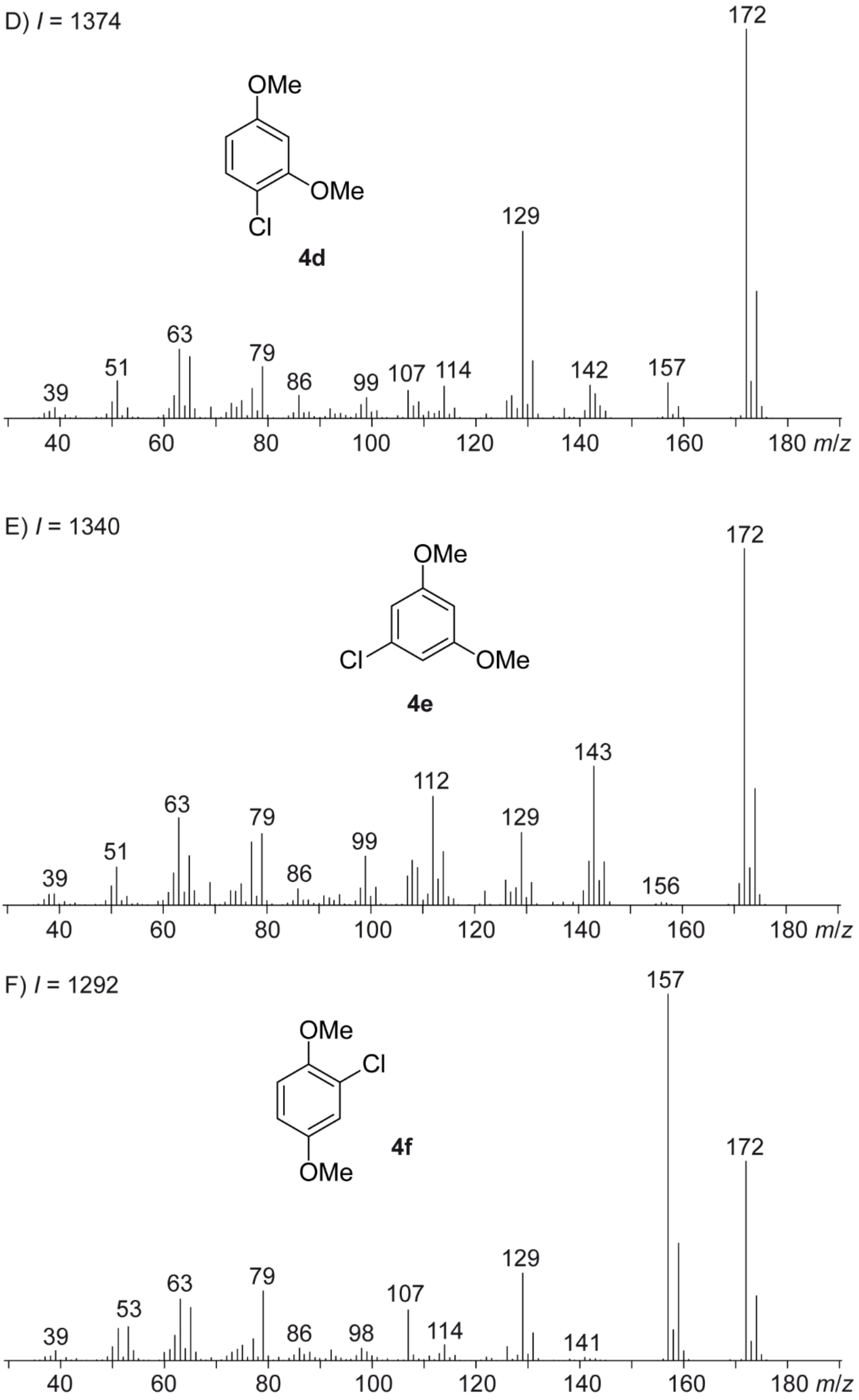

Figure 1 (continued): Mass spectra of chlorodimethoxybenzenes **4d**–**4f**.

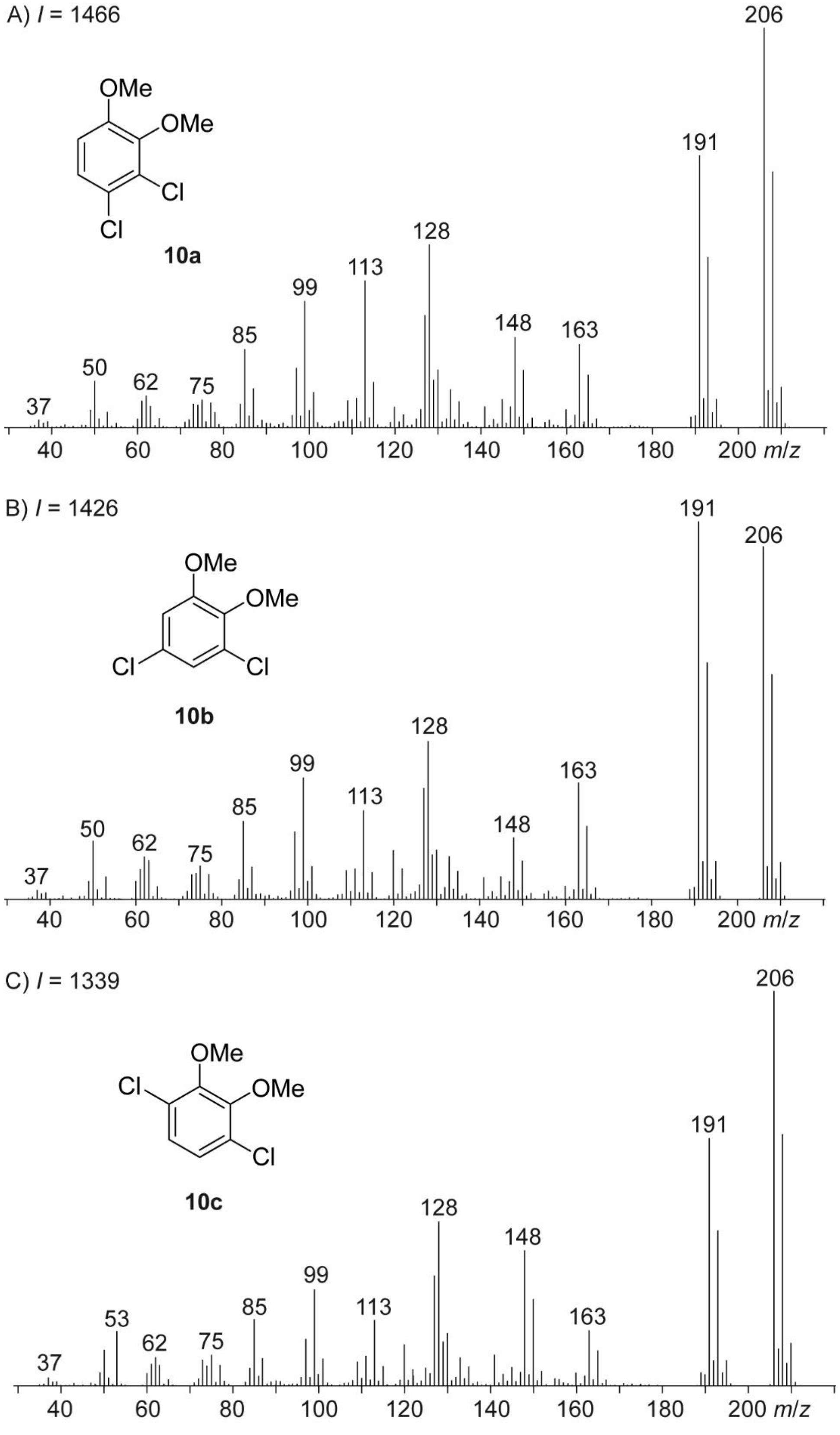

Figure 2: Mass spectra of dichlorodimethoxybenzenes **10a**–**10c**.

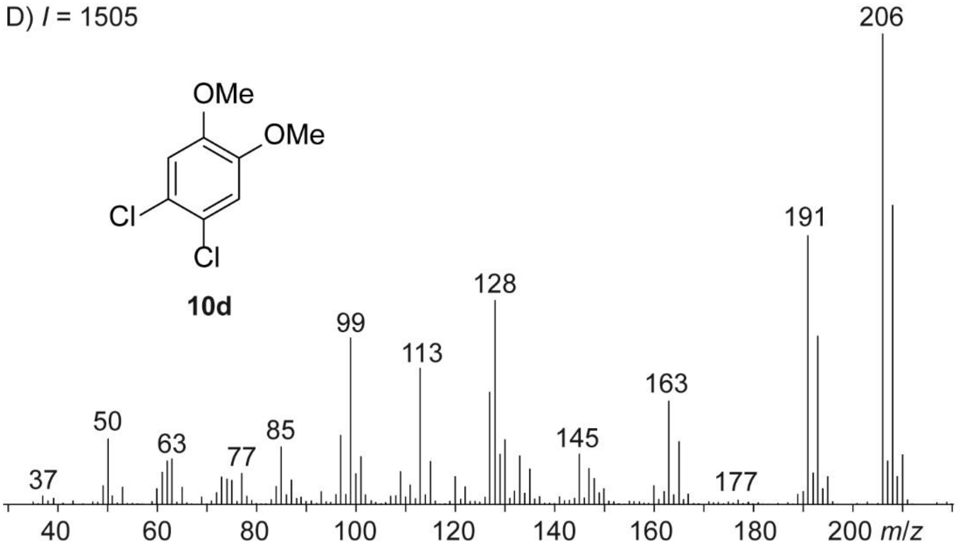

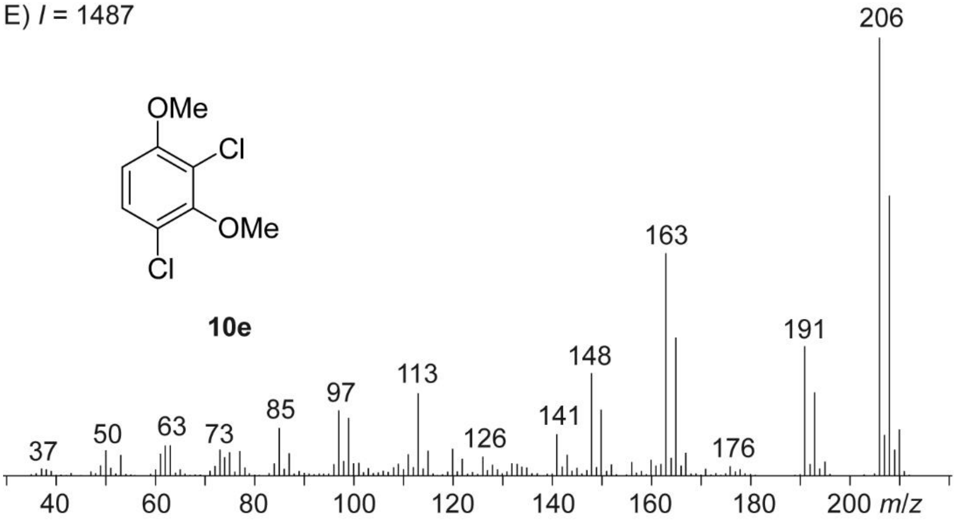

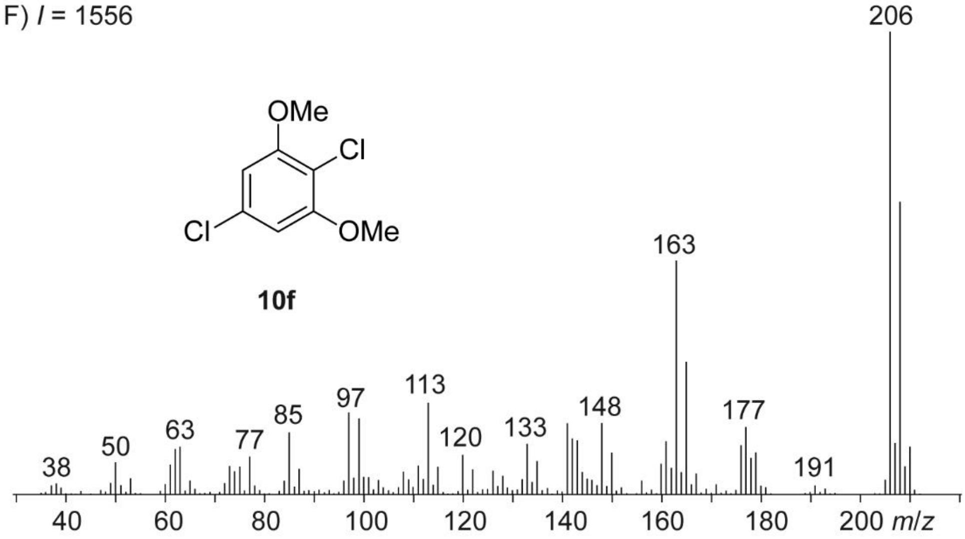

Figure 2 (continued): Mass spectra of dichlorodimethoxybenzenes **10d–10f**.

G) *I* = 1565

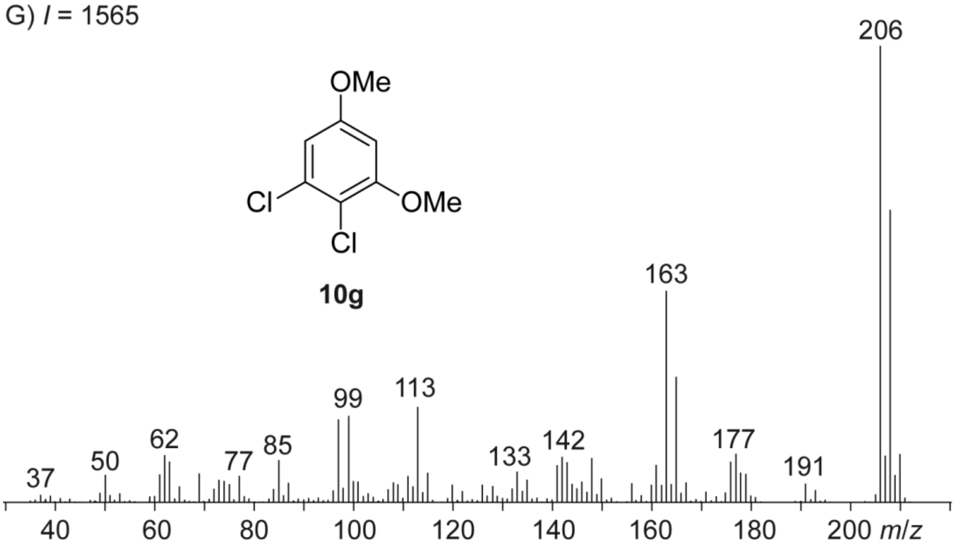

H) *I* = 1545

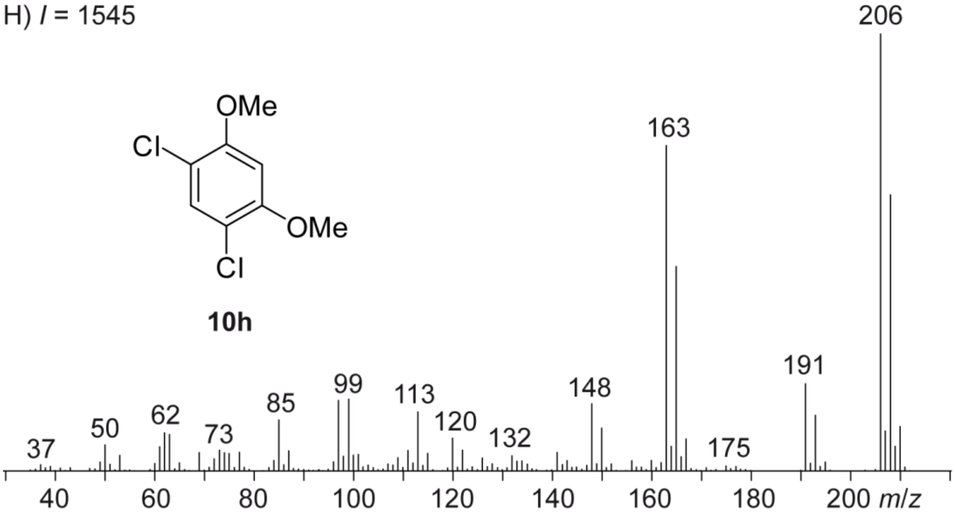

I) *I* = 1574

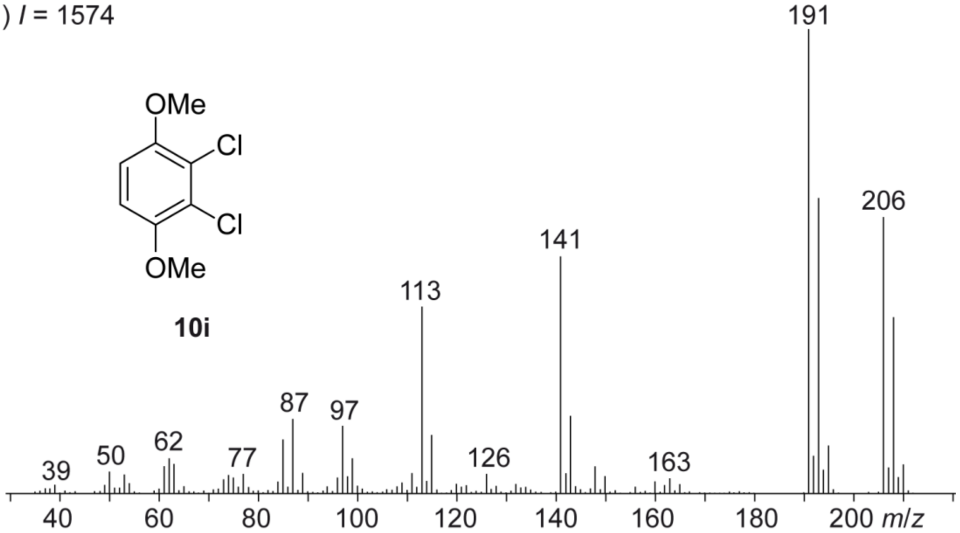

Figure 2 (continued): Mass spectra of dichlorodimethoxybenzenes **10g–10i**.

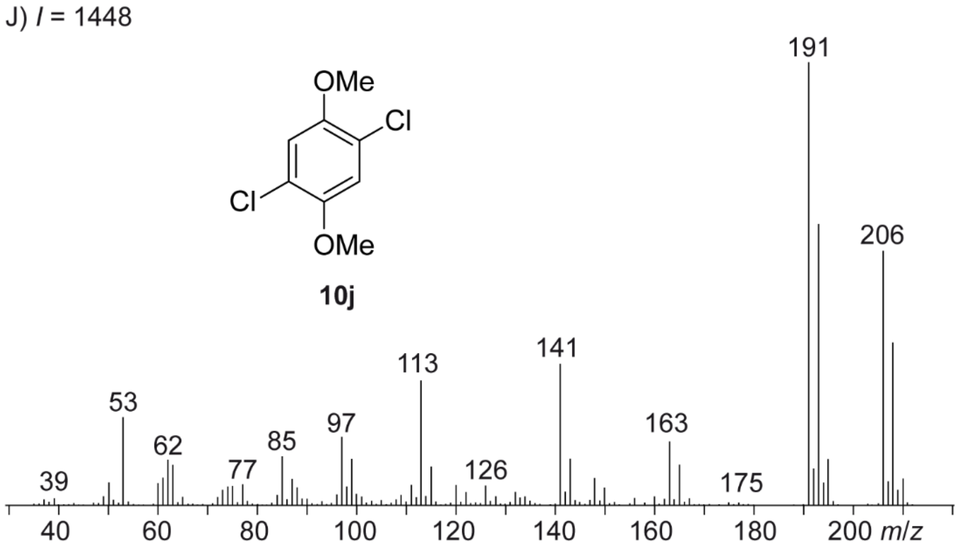

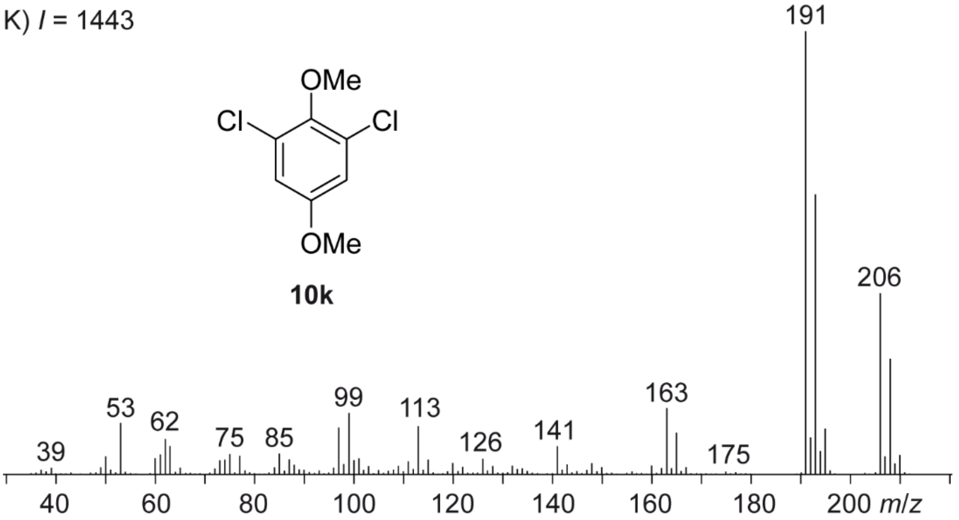

Figure 2 (continued): Mass spectra of dichlorodimethoxybenzenes **10j**–**10k**.

NMR spectra of synthetic compounds

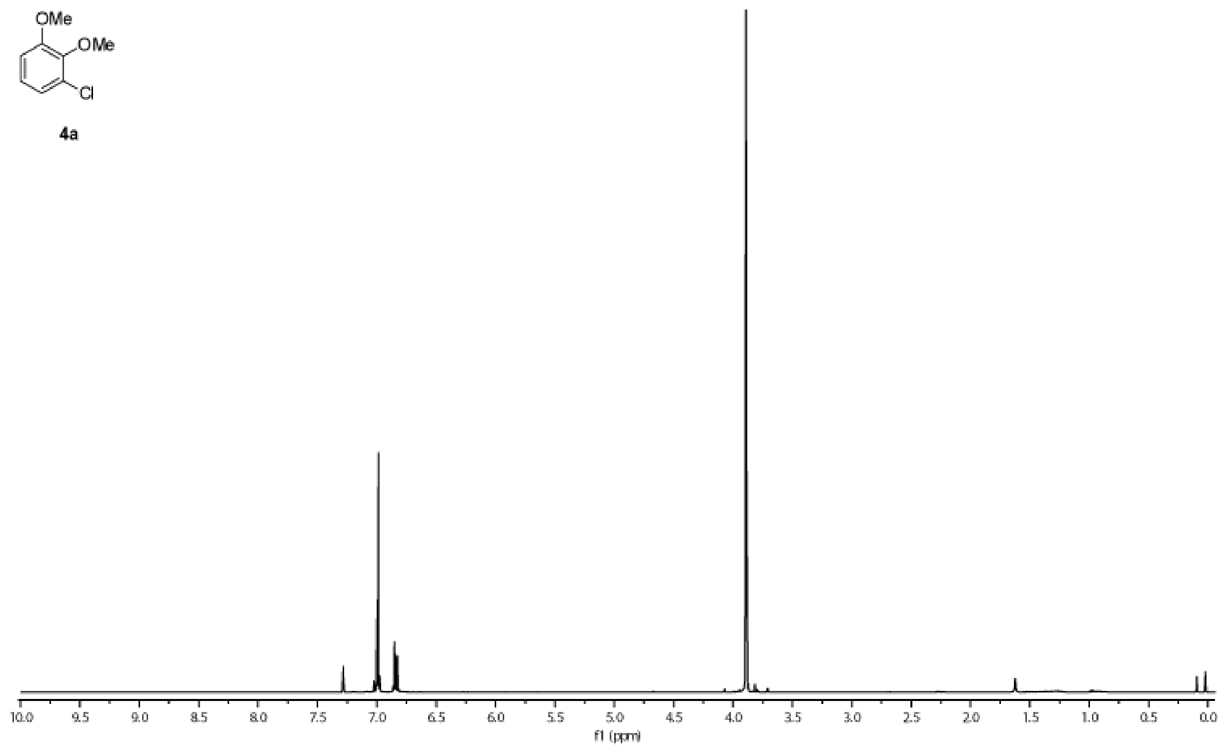

Figure 3: ^{1}H NMR spectrum of compound **4a**.

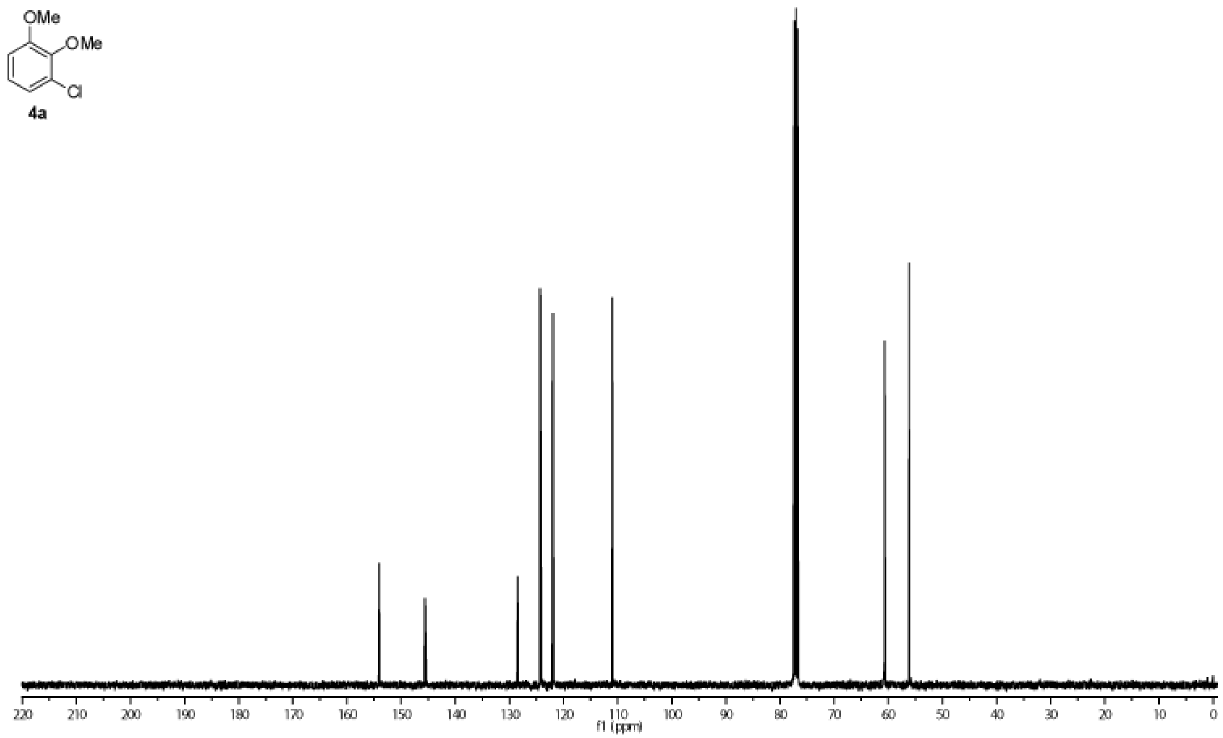

Figure 4: ^{13}C NMR spectrum of compound **4a**.

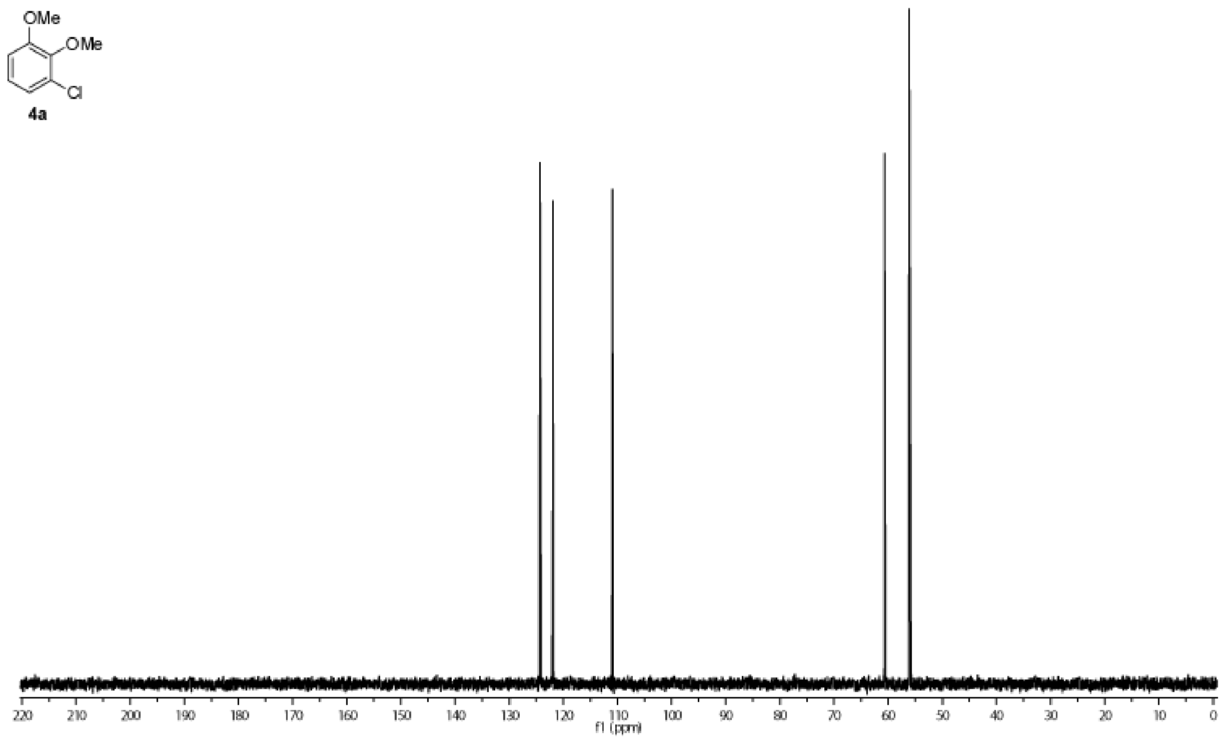

Figure 5: ^{13}C DEPT spectrum of compound **4a**.

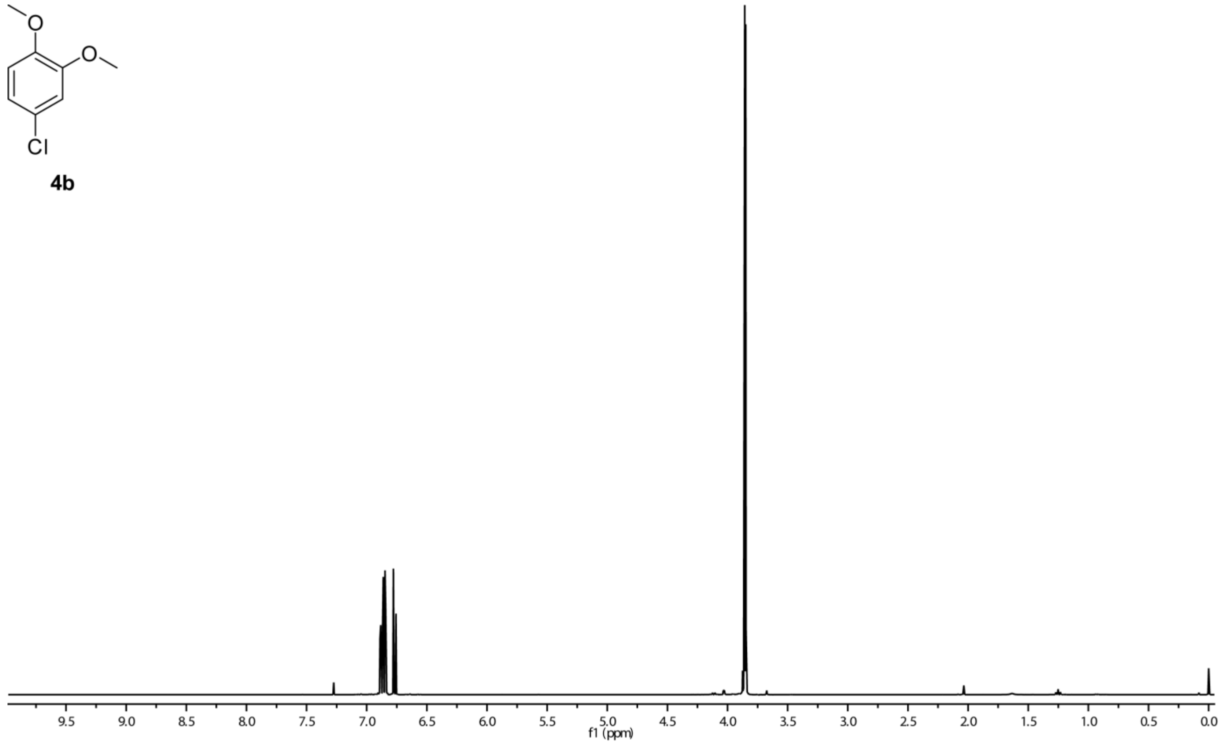

Figure 6: ^{1}H NMR spectrum of compound **4b**.

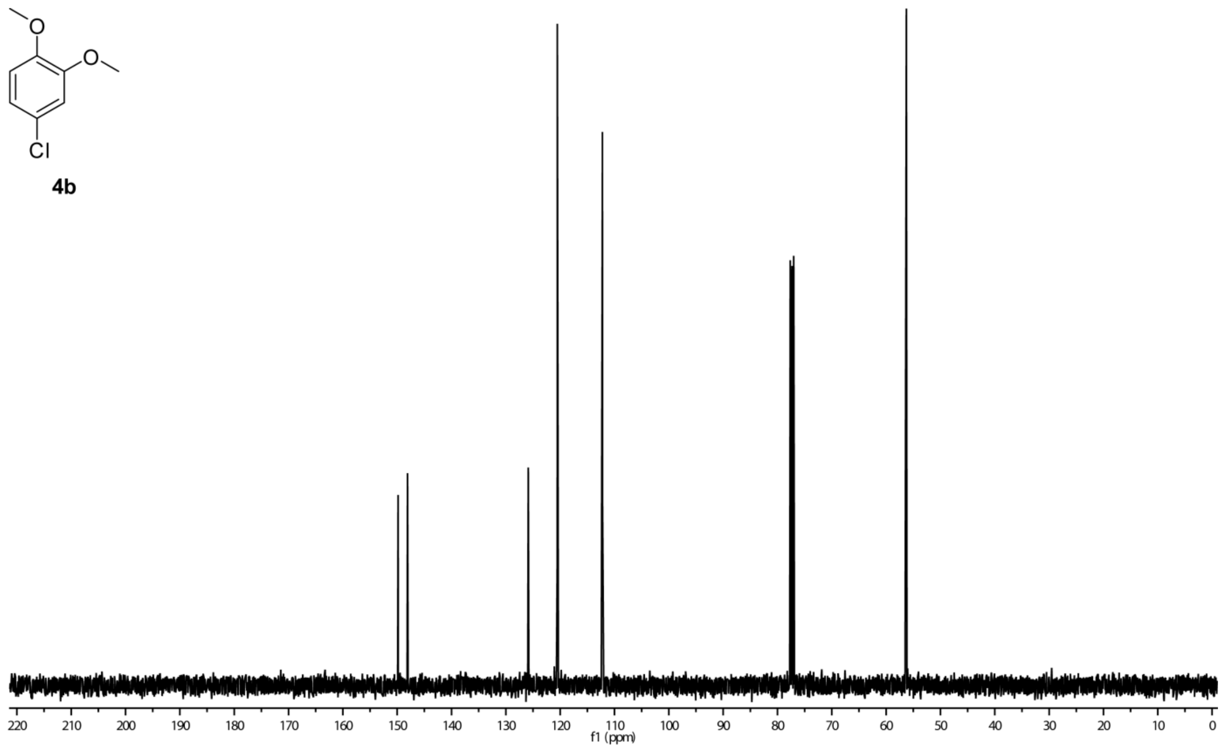

Figure 7: ^{13}C NMR spectrum of compound **4b**.

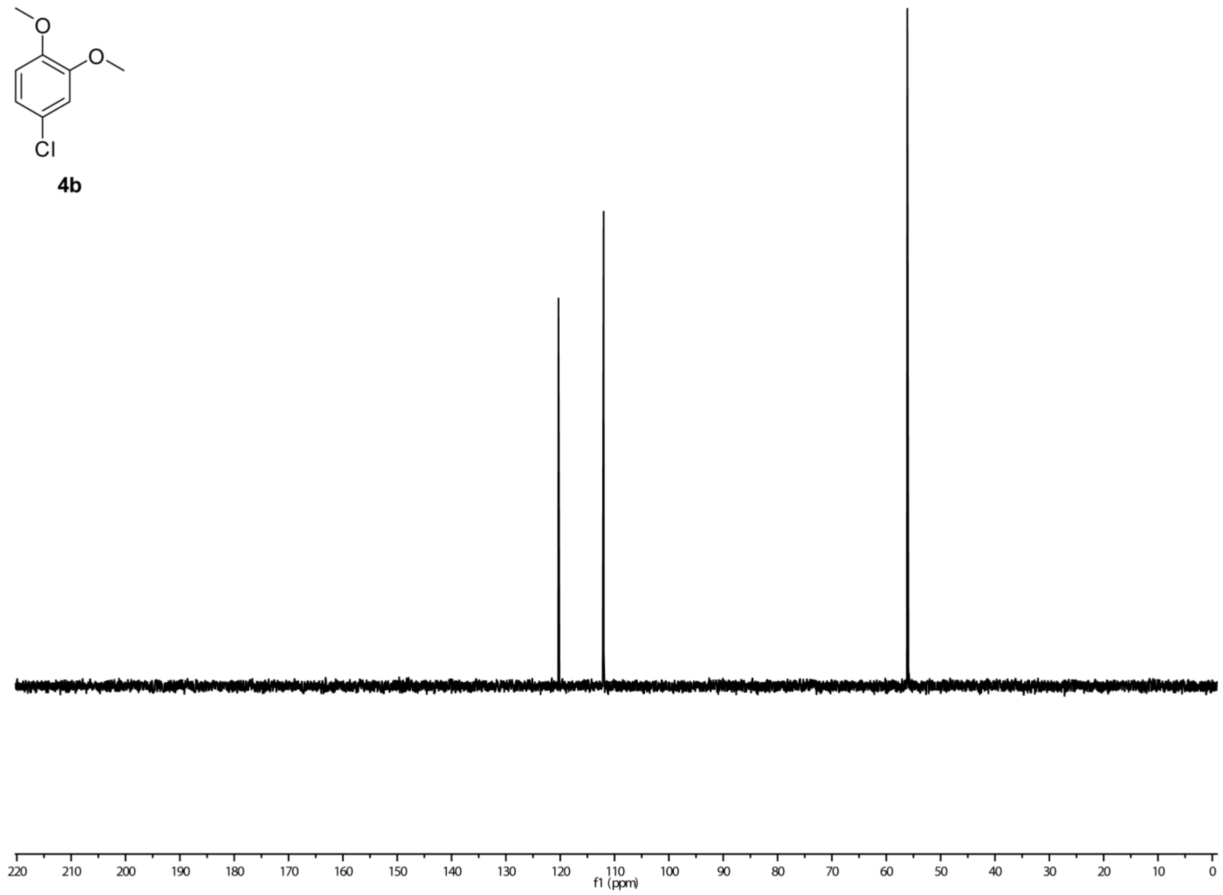

Figure 8: ^{13}C DEPT spectrum of compound **4b**.

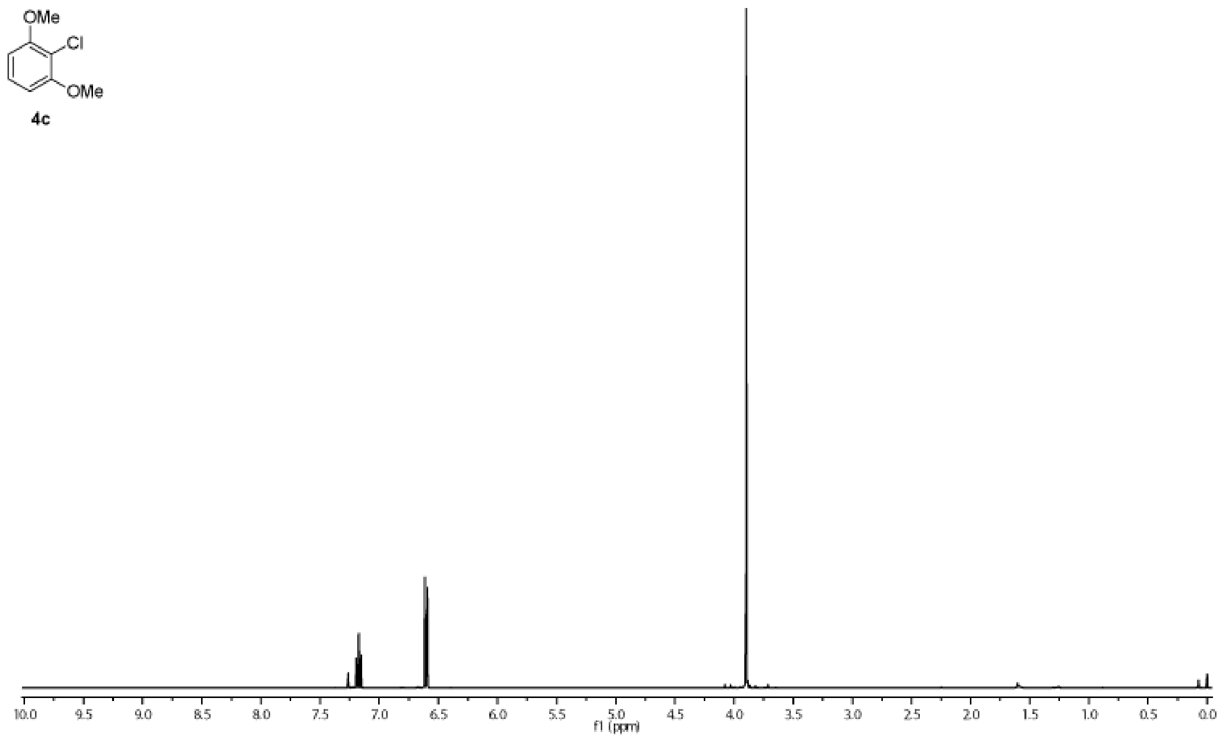

Figure 9: ^{1}H NMR spectrum of compound **4c**.

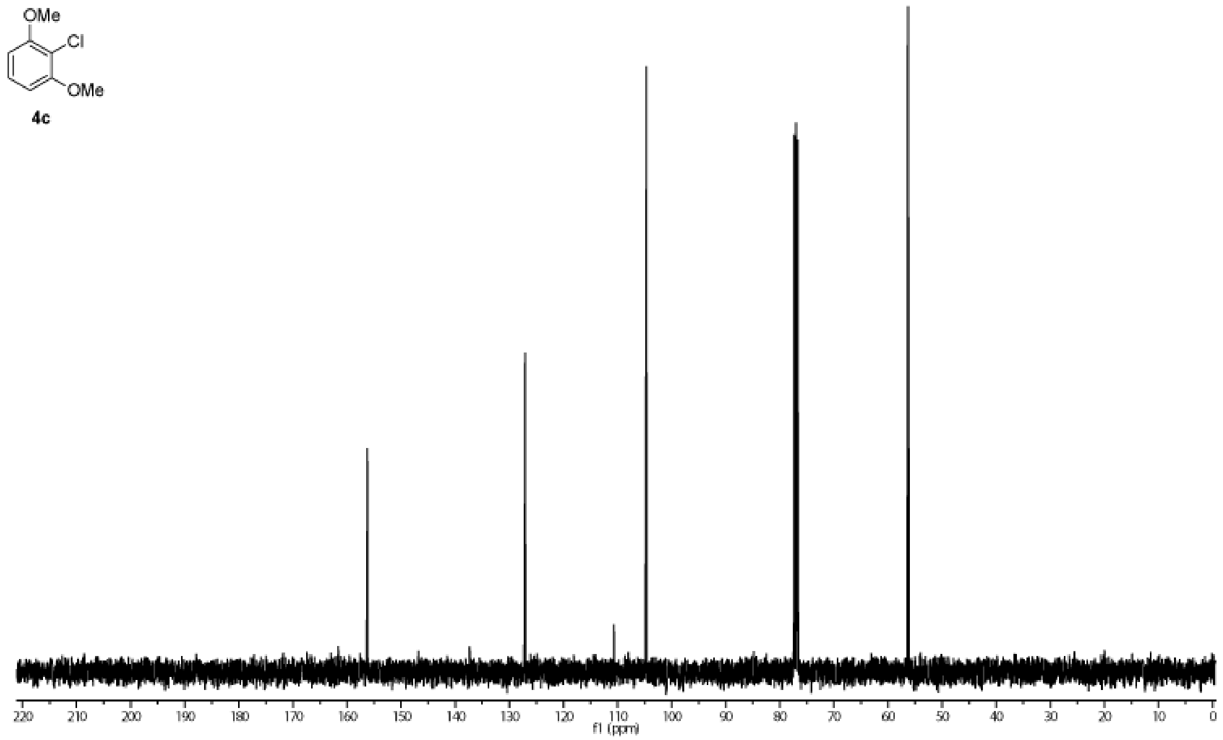

Figure 10: ^{13}C NMR spectrum of compound **4c**.

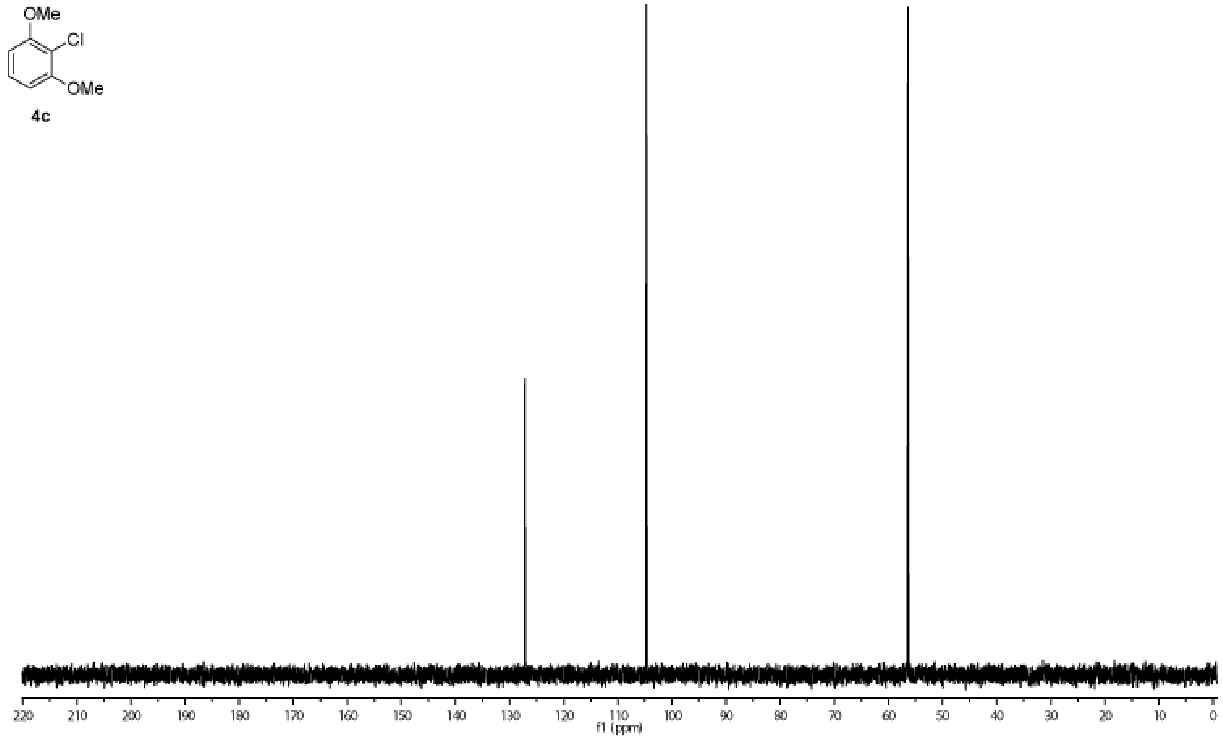

Figure 11: ^{13}C DEPT spectrum of compound **4c**.

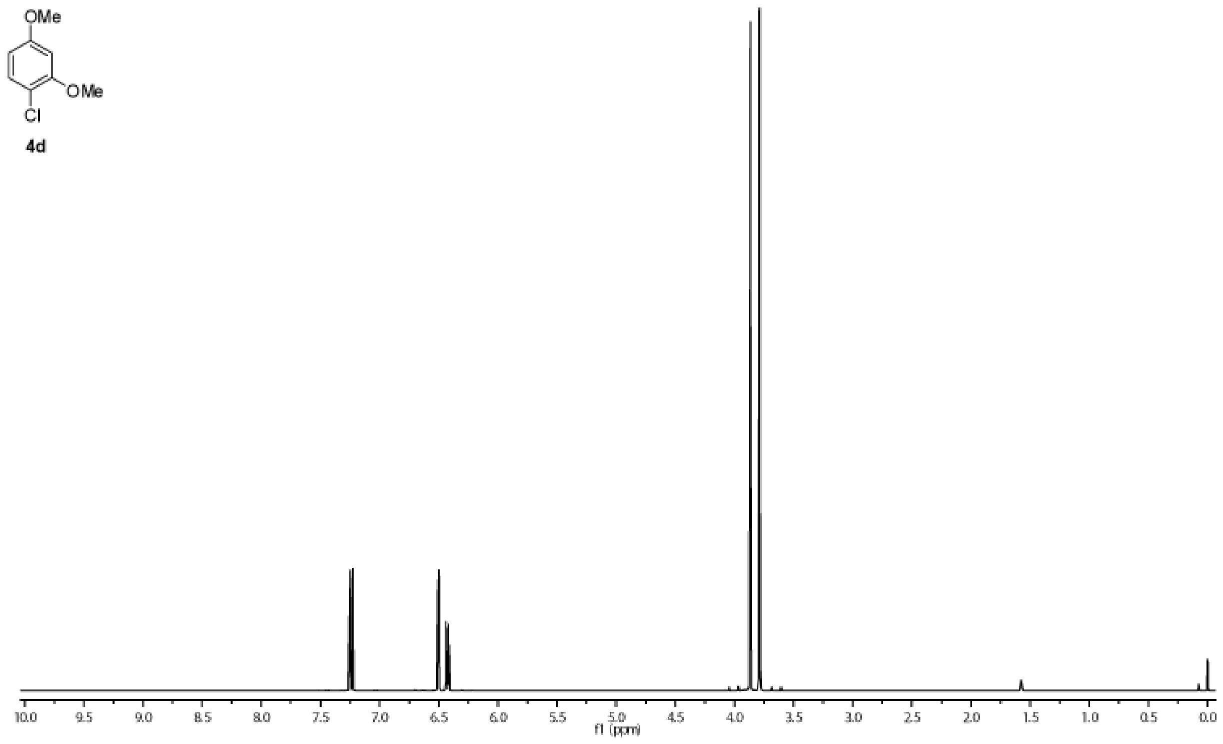

Figure 12: ^{1}H NMR spectrum of compound **4d**.

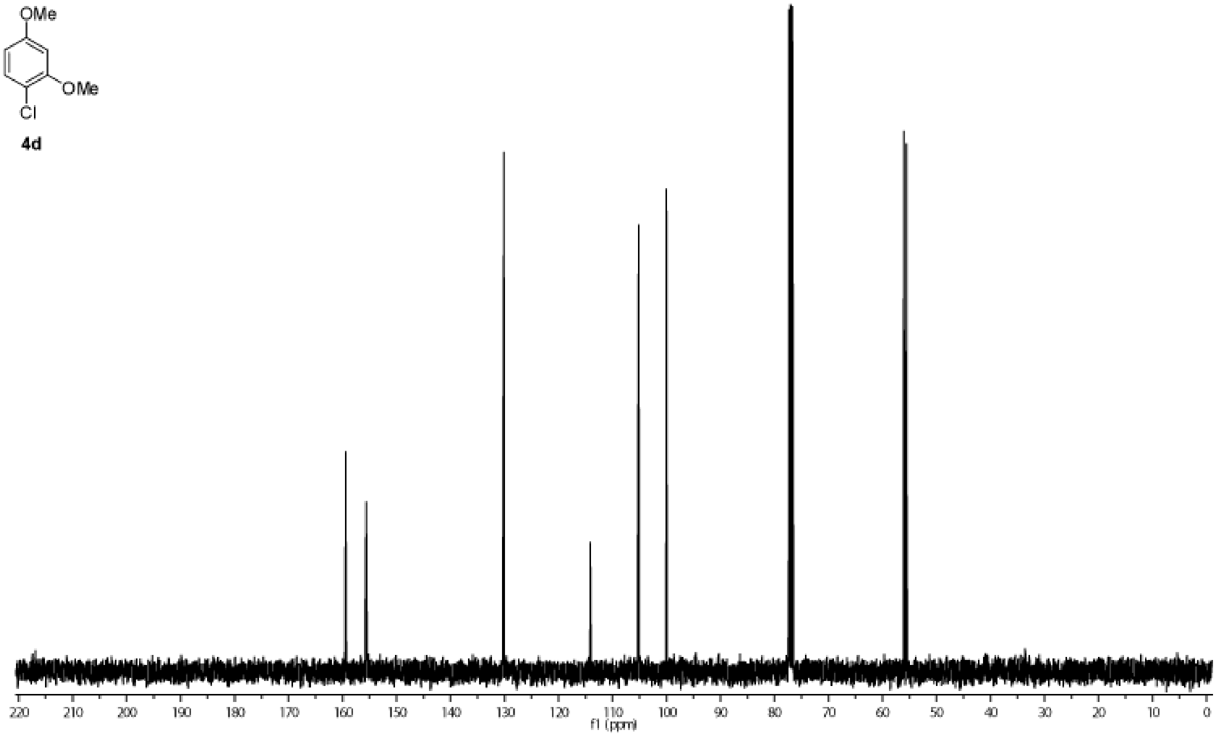

Figure 13: ^{13}C NMR spectrum of compound **4d**.

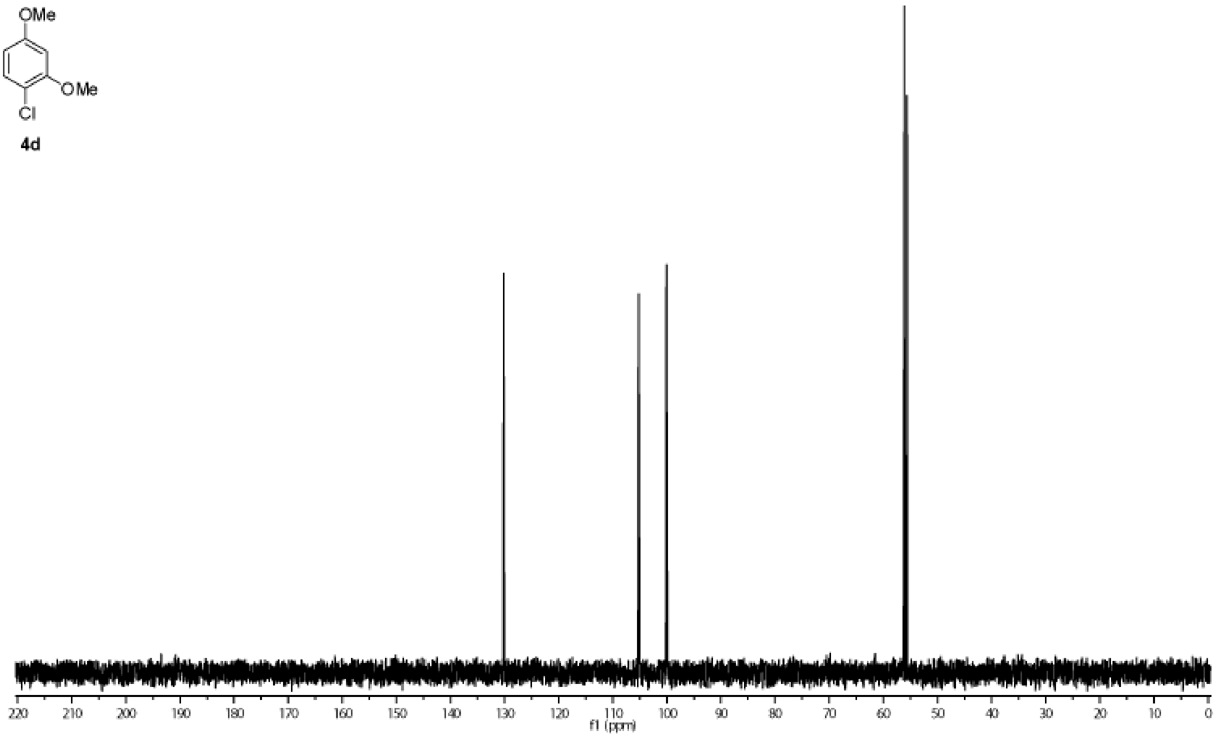

Figure 14: ^{13}C DEPT spectrum of compound **4d**.

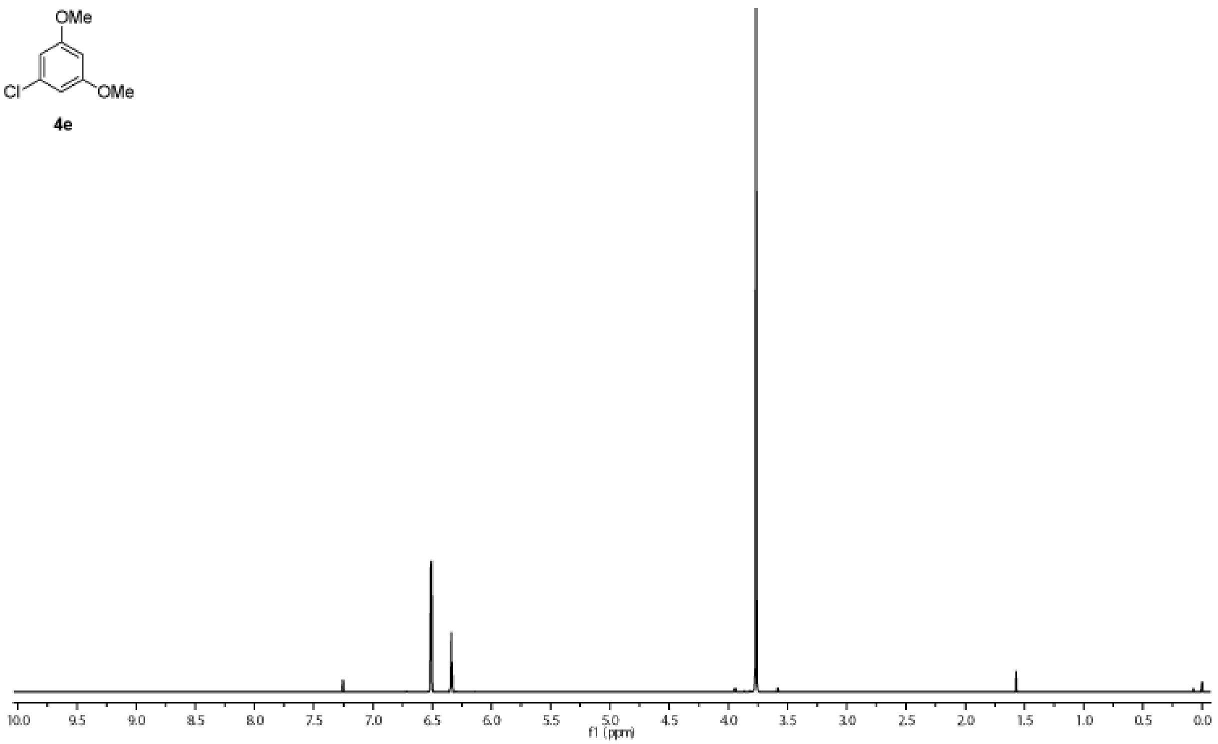

Figure 15: ^{1}H NMR spectrum of compound **4e**.

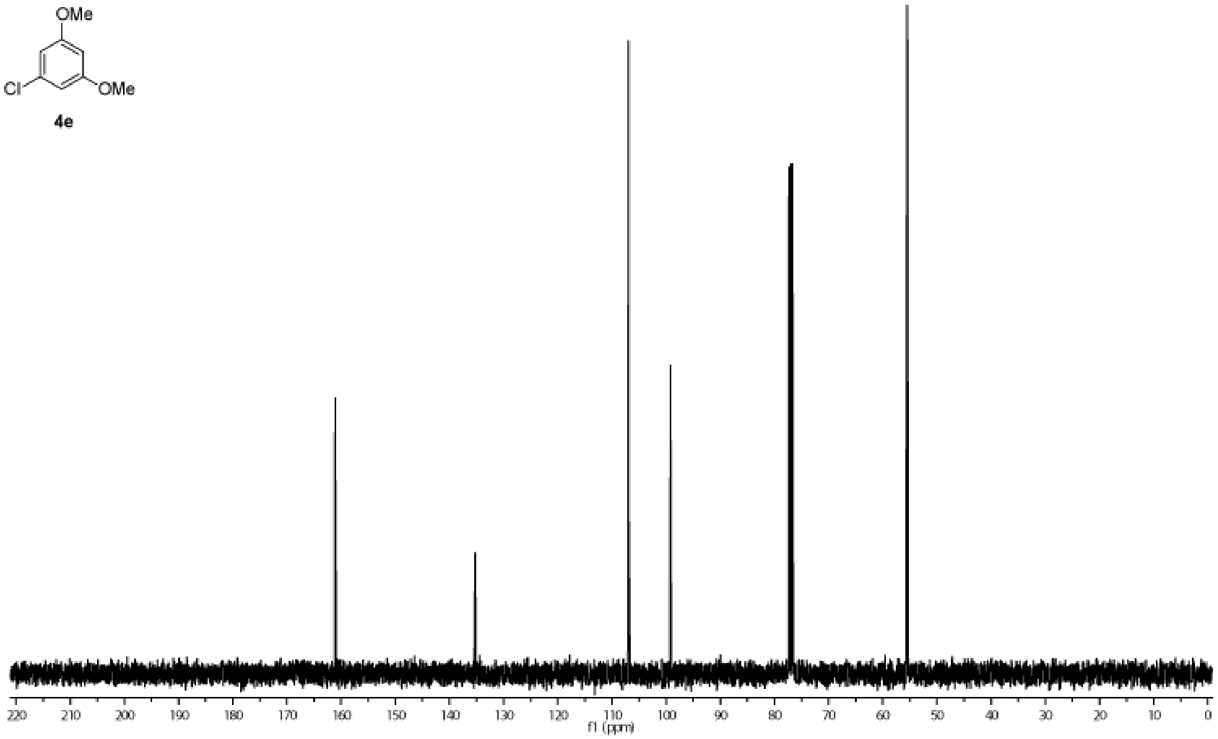

Figure 16: ^{13}C NMR spectrum of compound **4e**.

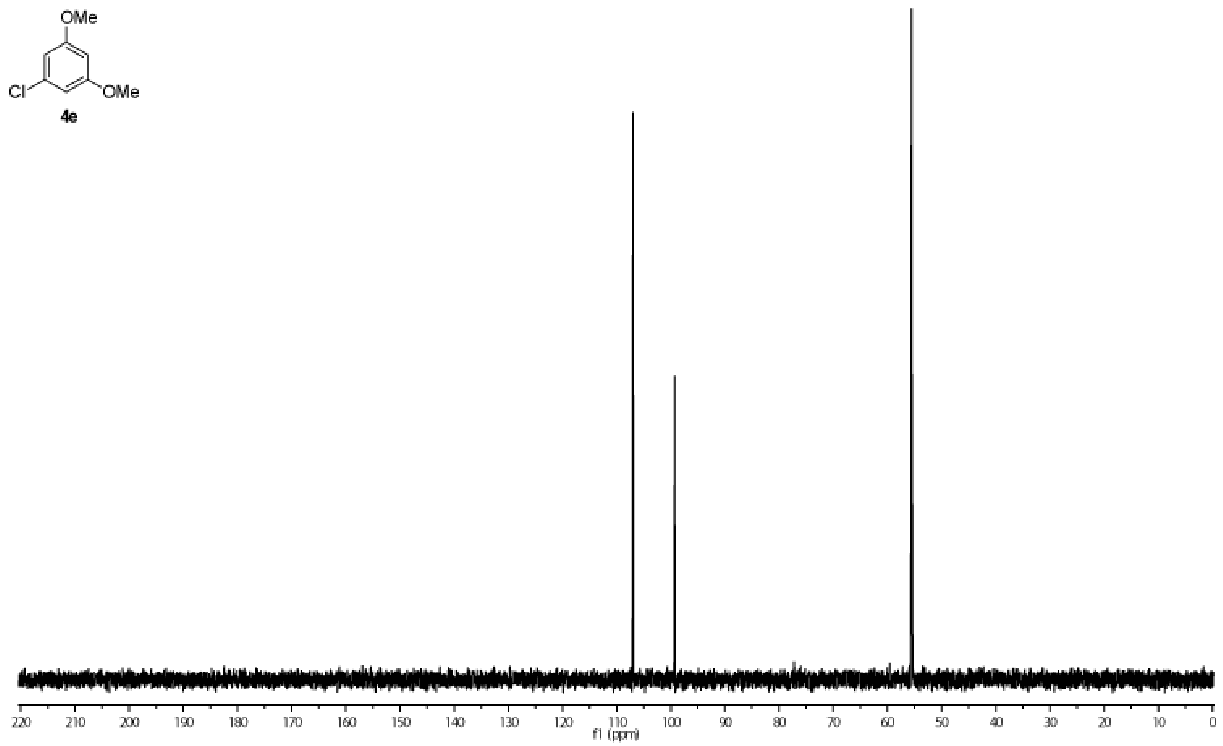

Figure 17: ^{13}C DEPT spectrum of compound **4e**.

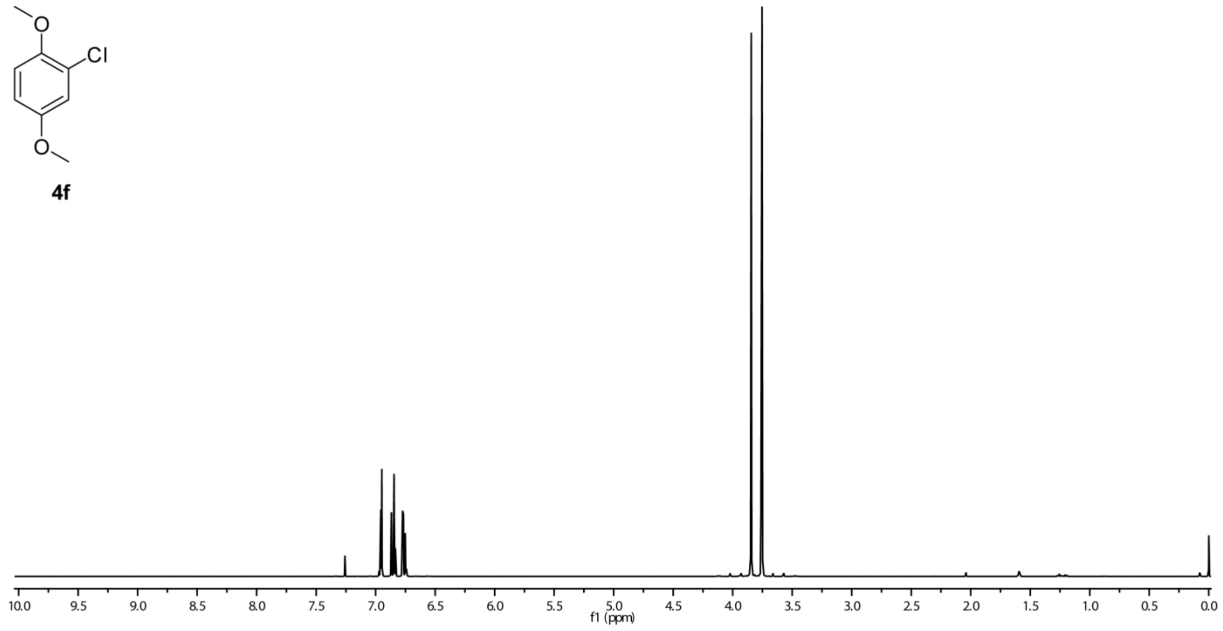

Figure 18: ^{1}H NMR spectrum of compound **4f**.

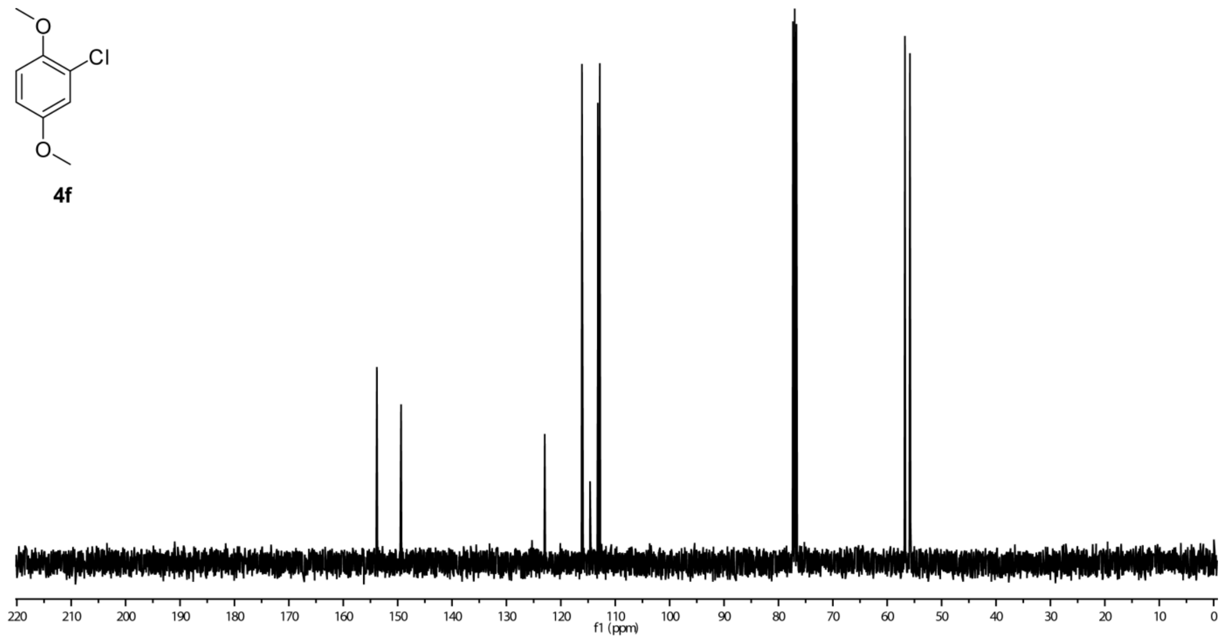

Figure 19: ^{13}C NMR spectrum of compound **4f**.

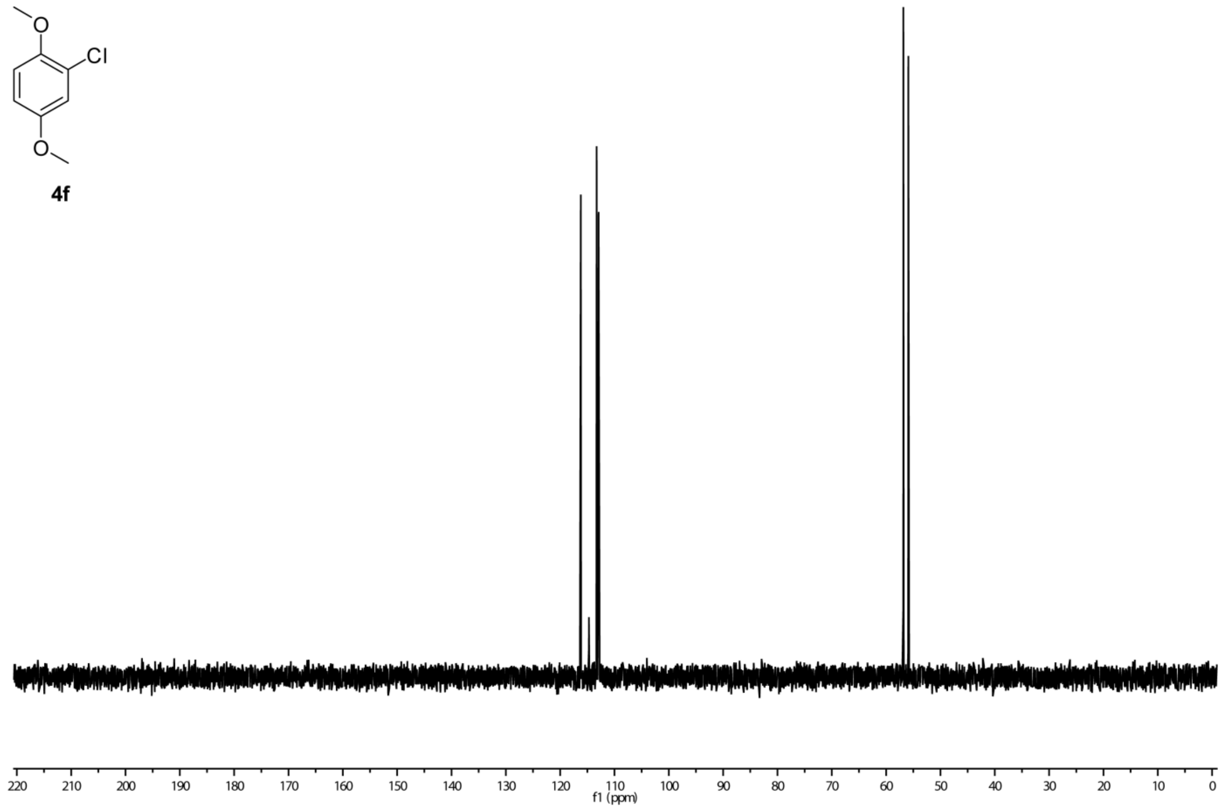

Figure 20: ^{13}C DEPT spectrum of compound **4f**.

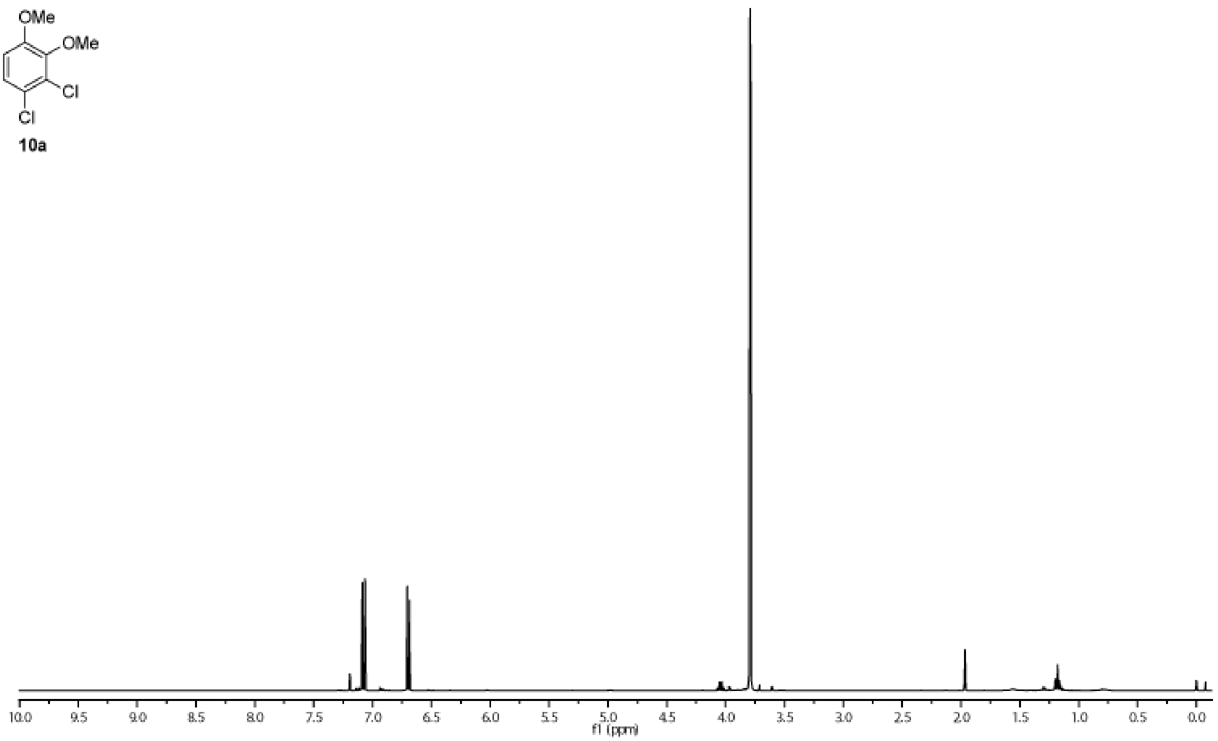

Figure 21: ^{1}H NMR spectrum of compound **10a**.

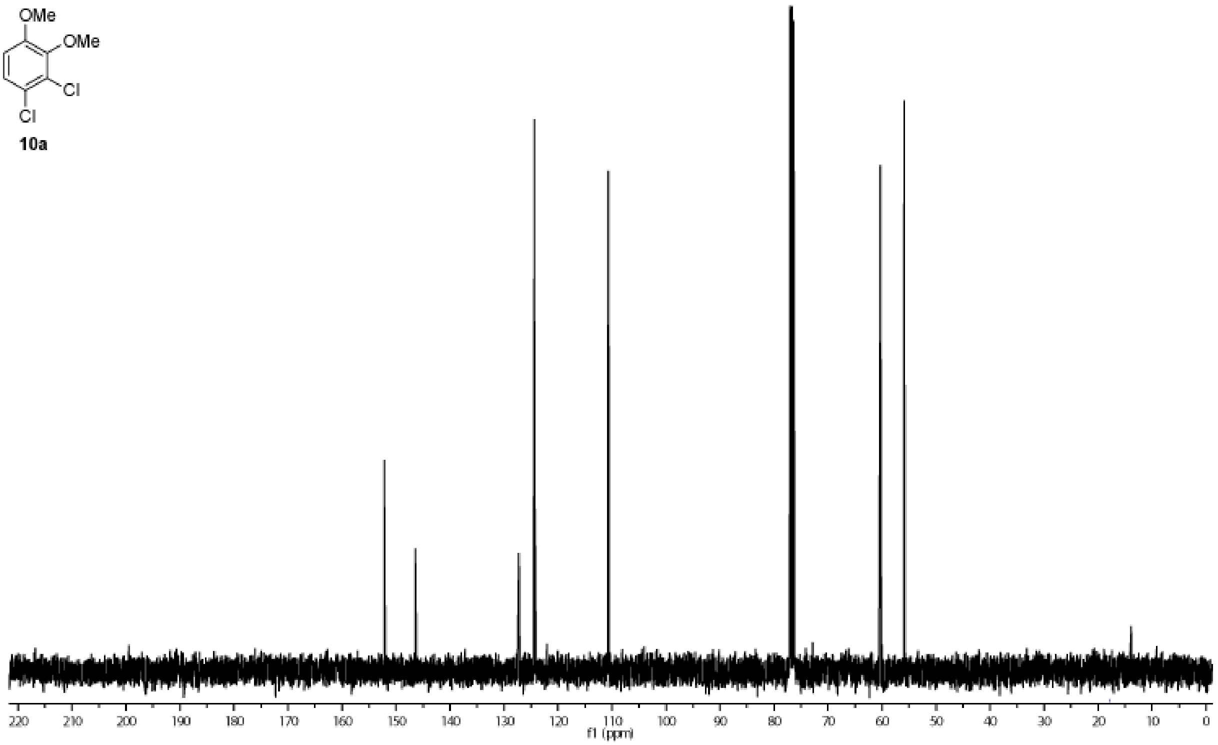

Figure 22: ^{13}C NMR spectrum of compound **10a**.

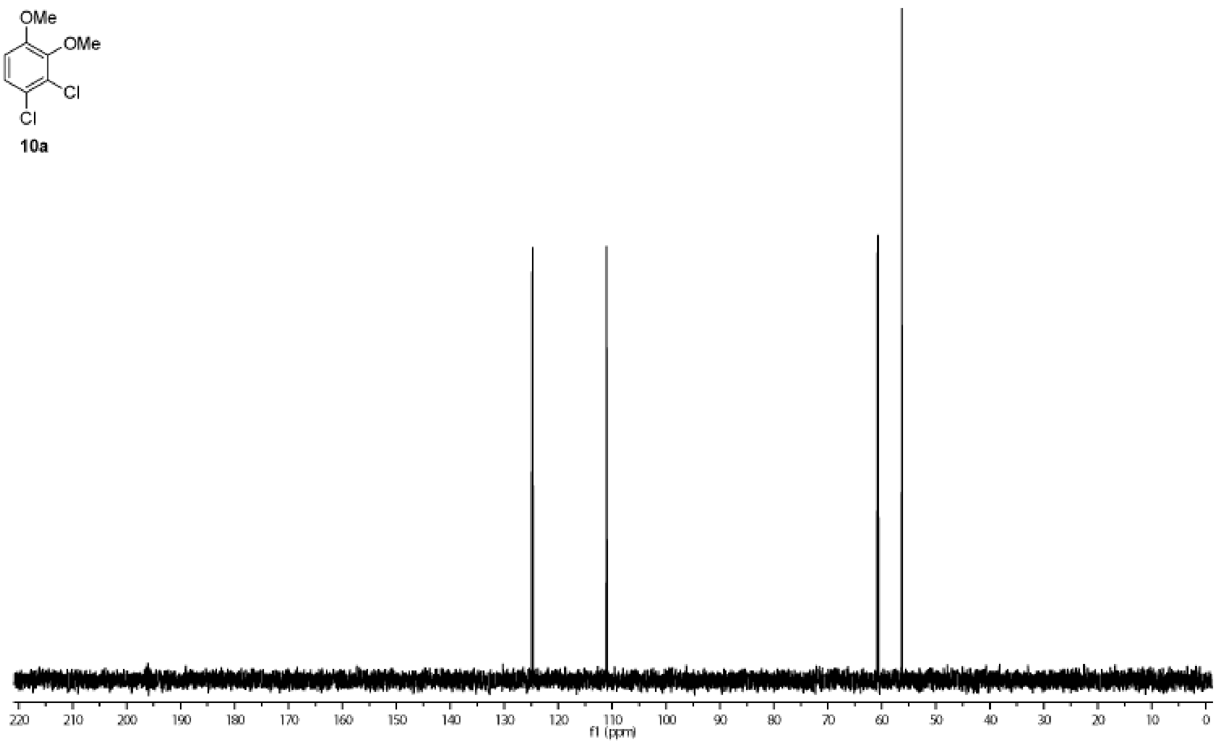

Figure 23: ^{13}C DEPT spectrum of compound **10a**.

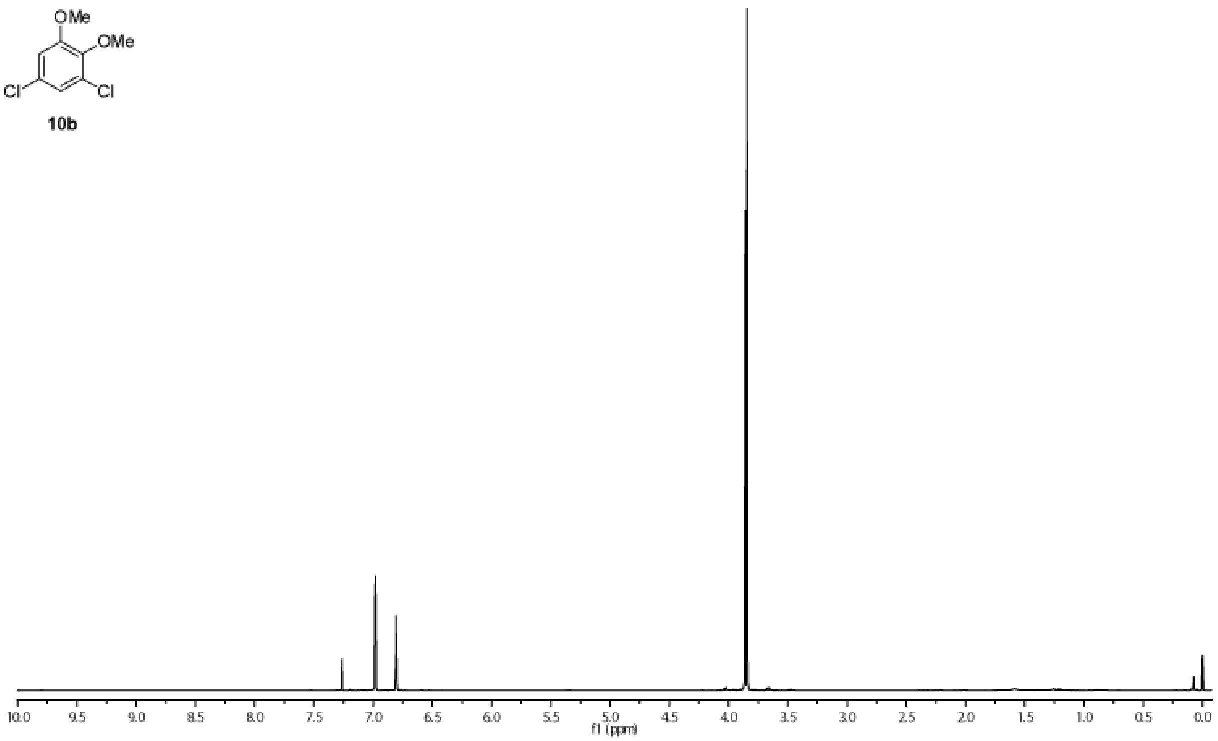

Figure 24: ^{1}H NMR spectrum of compound **10b**.

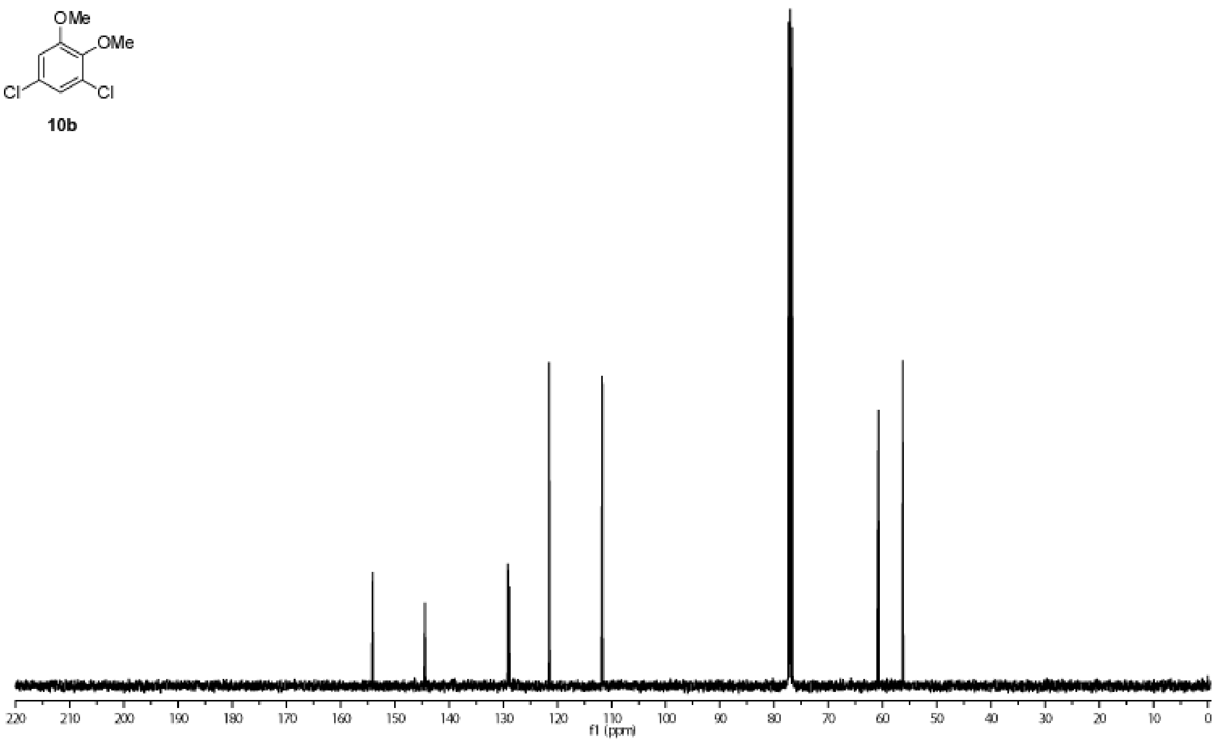

Figure 25: ^{13}C NMR spectrum of compound **10b**.

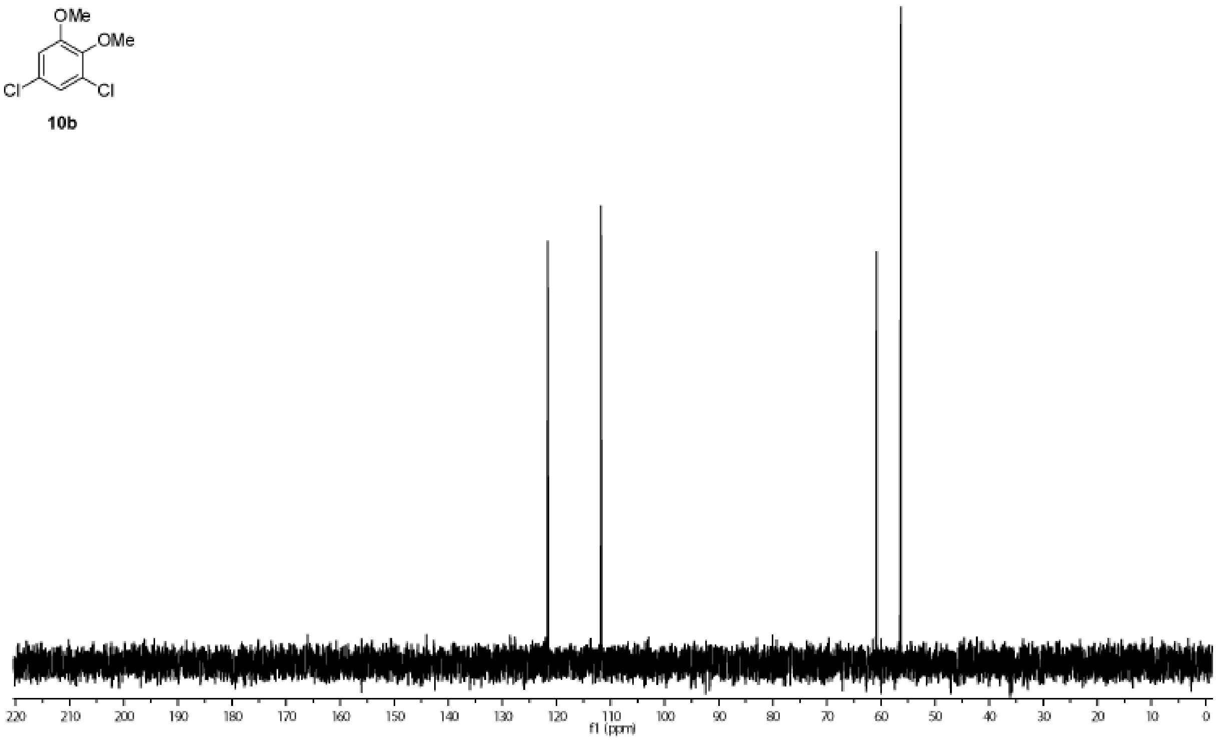

Figure 26: ^{13}C DEPTspectrum of compound **10b**.

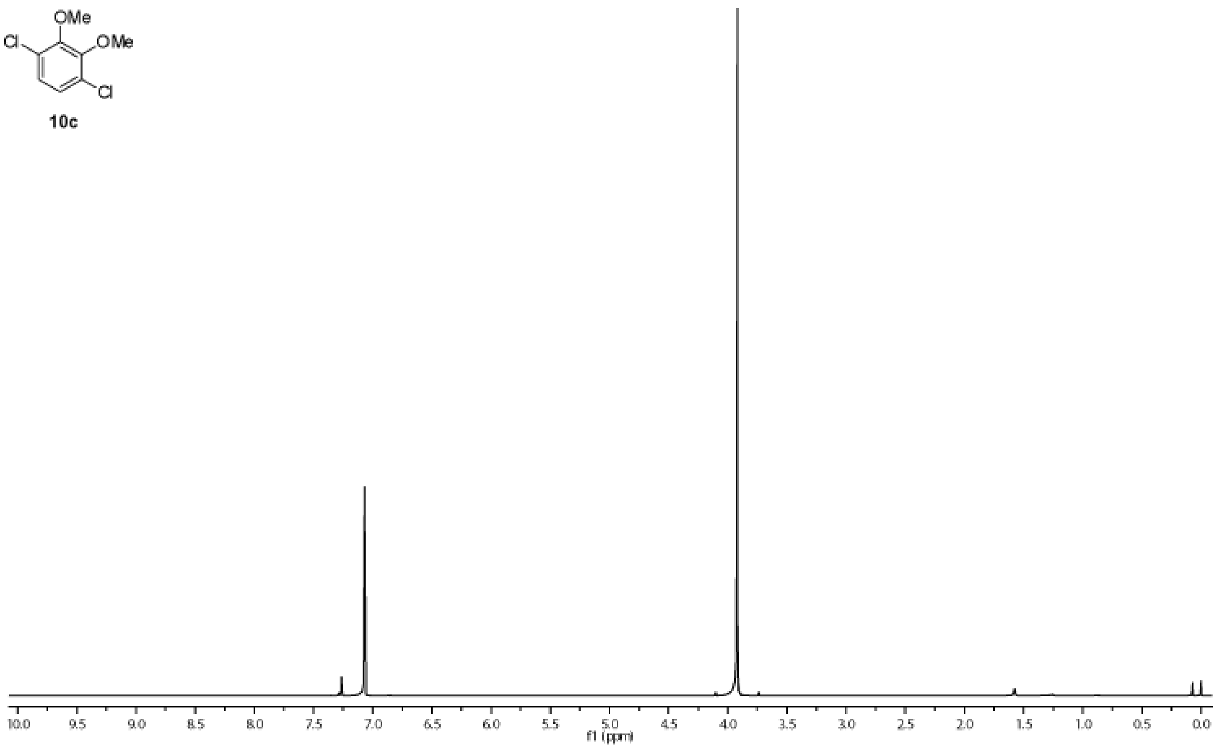

Figure 27: ^{1}H NMR spectrum of compound **10c**.

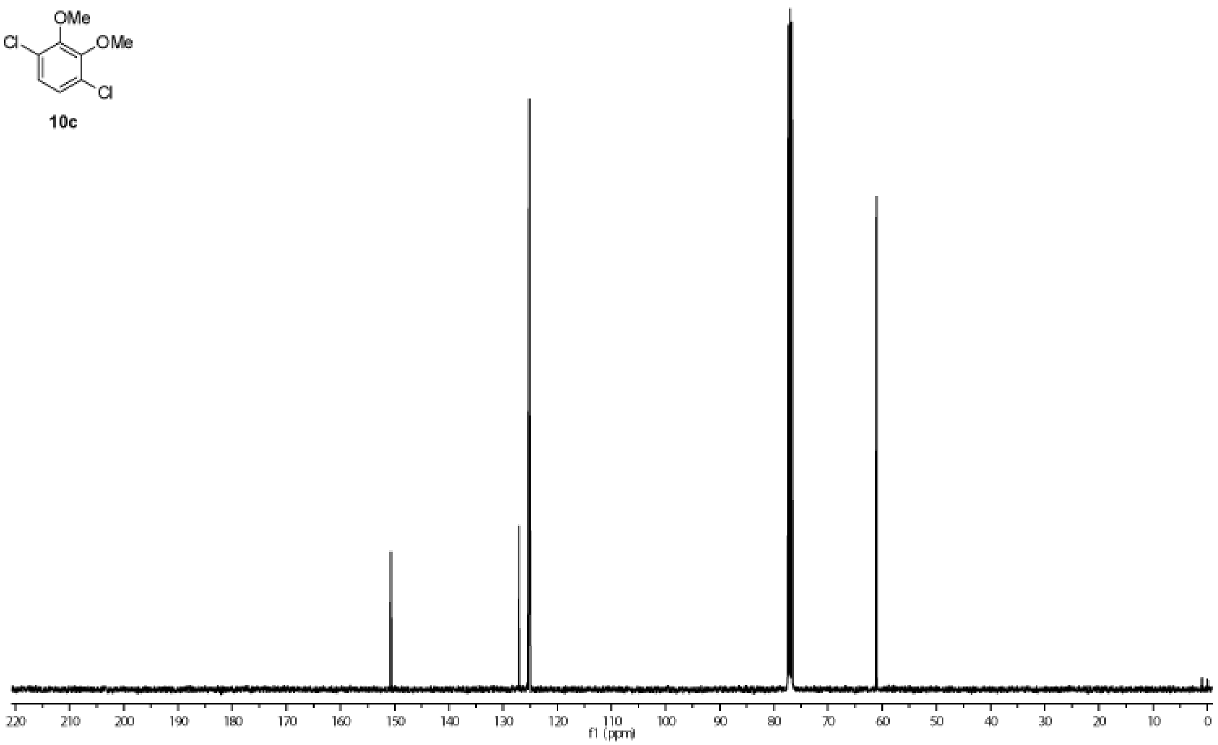

Figure 28: ^{13}C NMR spectrum of compound **10c**.

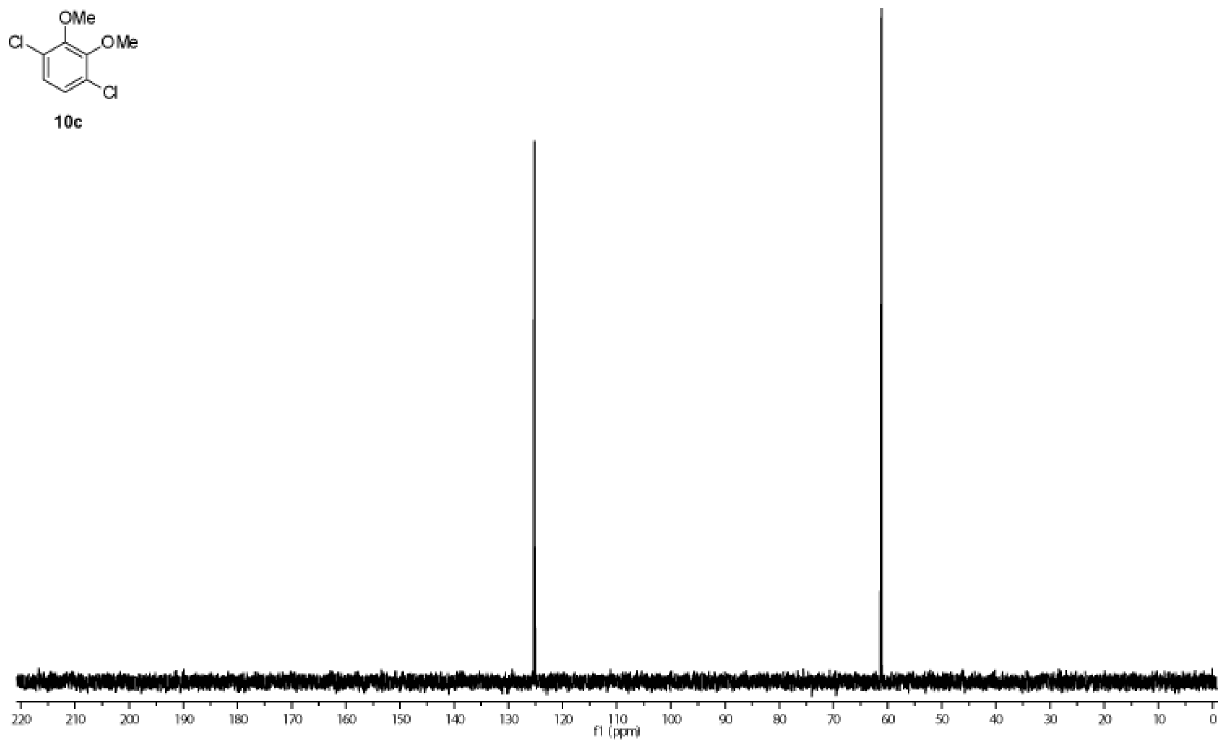

Figure 29: ^{13}C DEPT spectrum of compound **10c**.

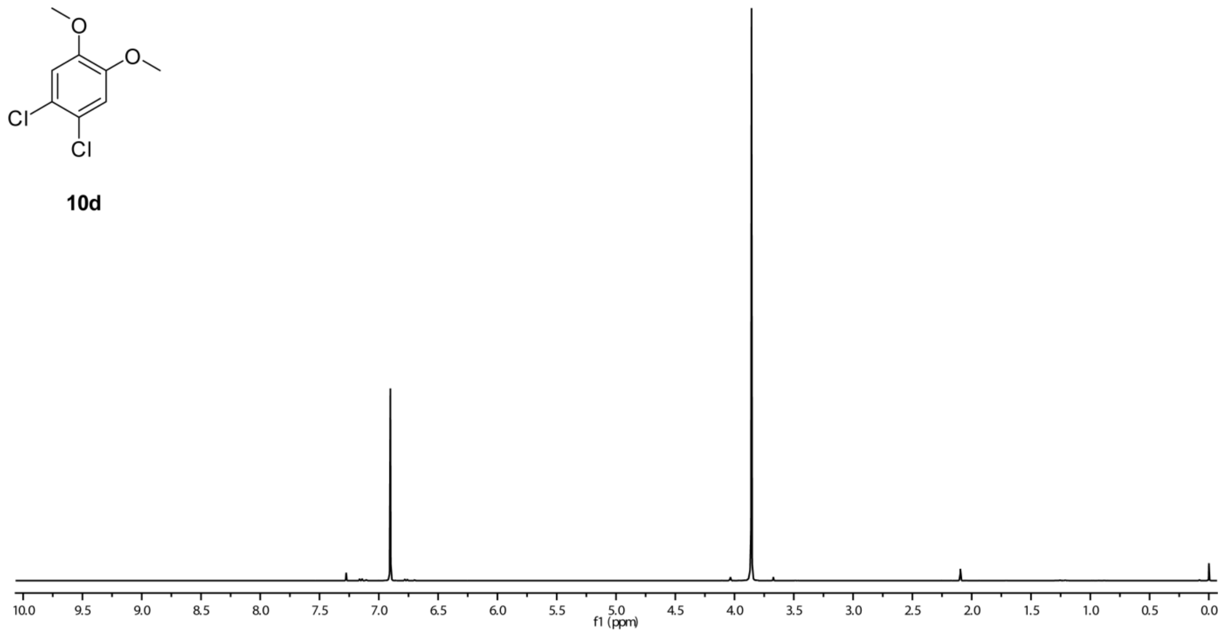

Figure 30: ^{1}H NMR spectrum of compound **10d**.

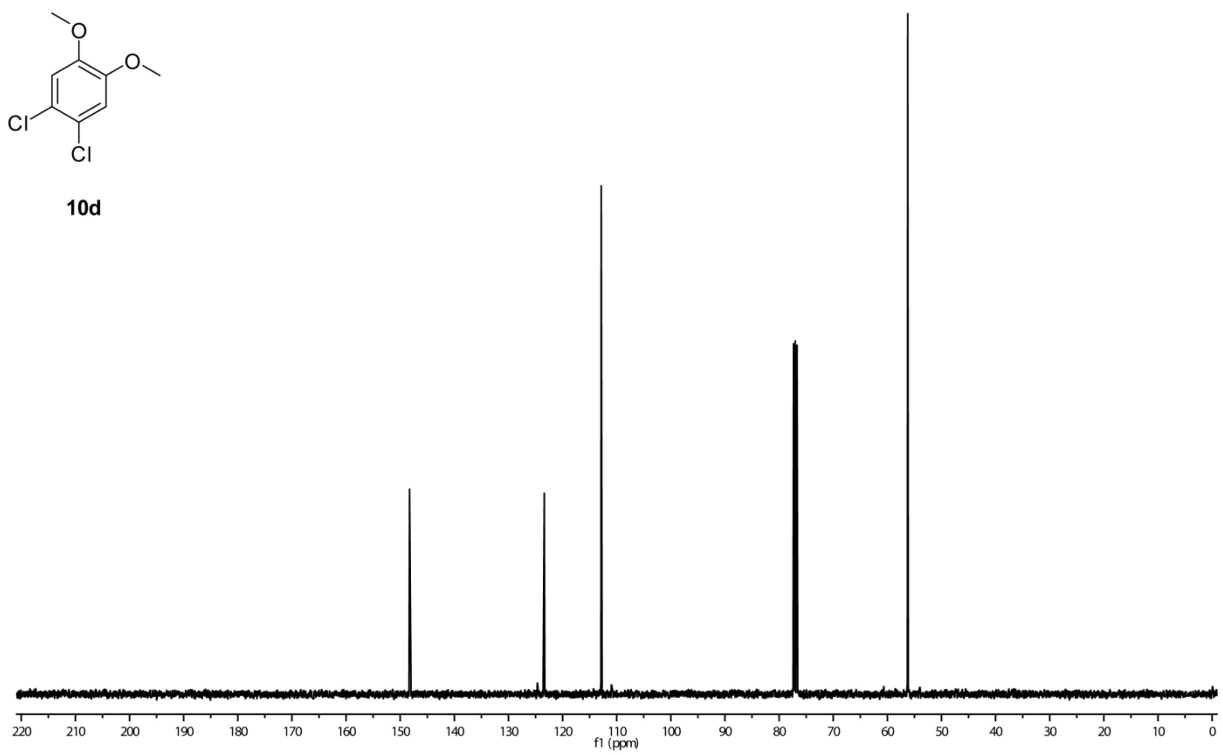

Figure 31: ^{13}C NMR spectrum of compound **10d**.

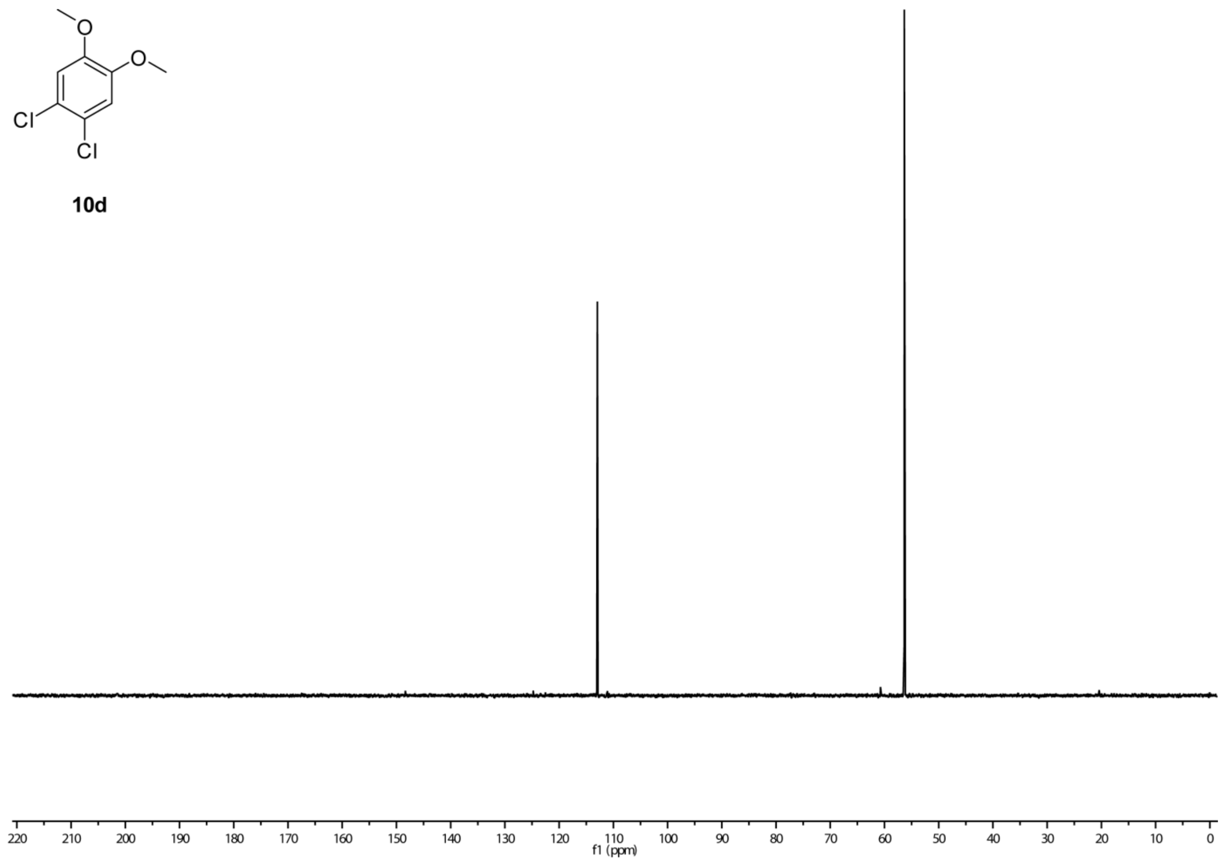

Figure 32: ^{13}C DEPT spectrum of compound **10d**.

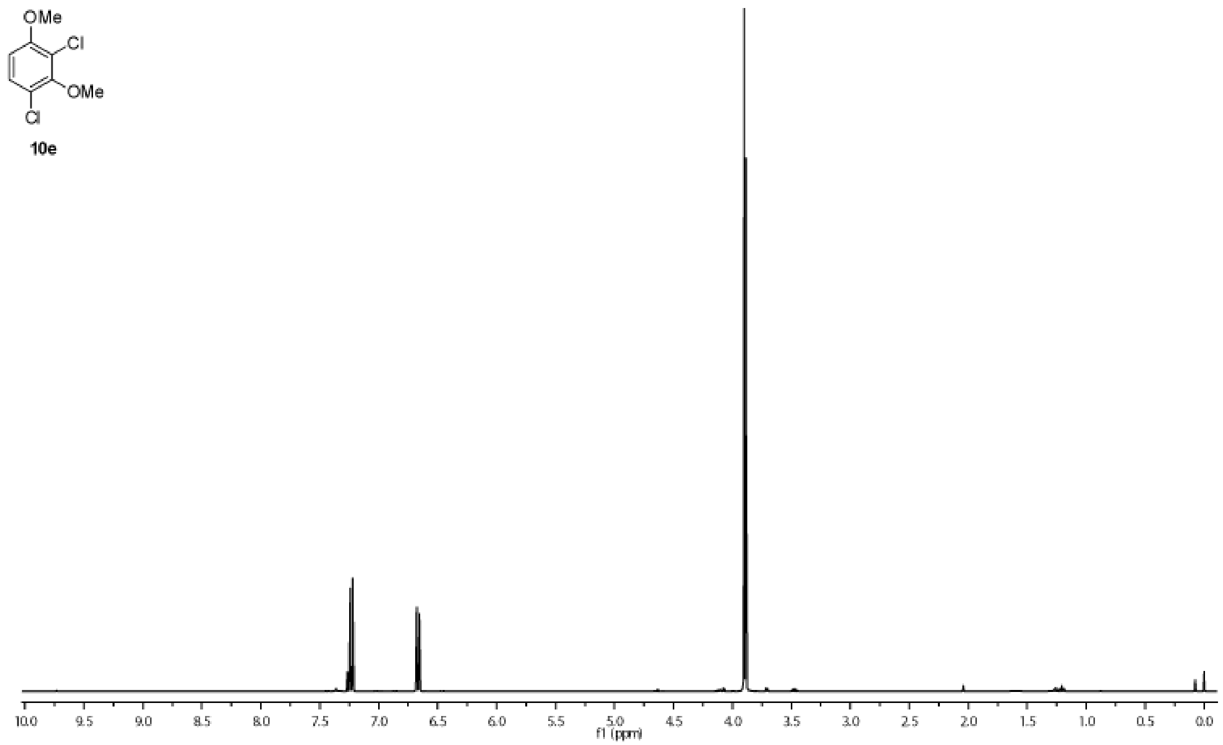

Figure 33: ^{1}H NMR spectrum of compound **10e**.

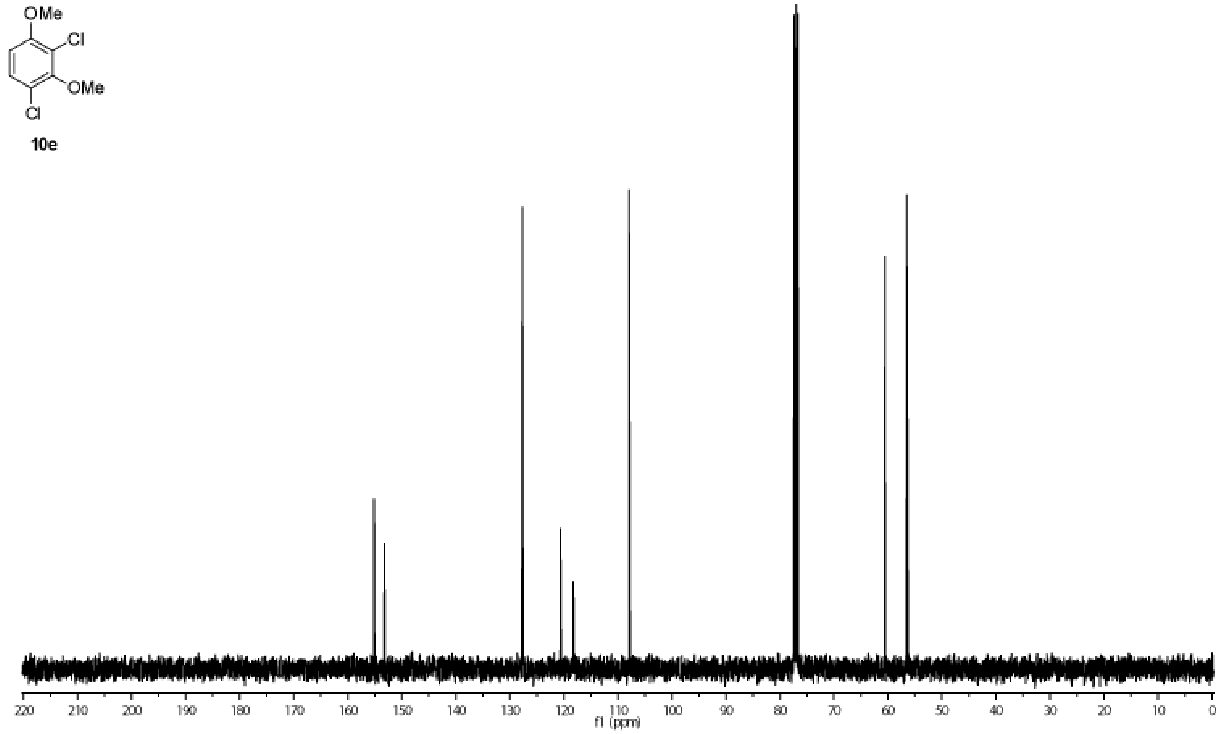

Figure 34: ^{13}C NMR spectrum of compound **10e**.

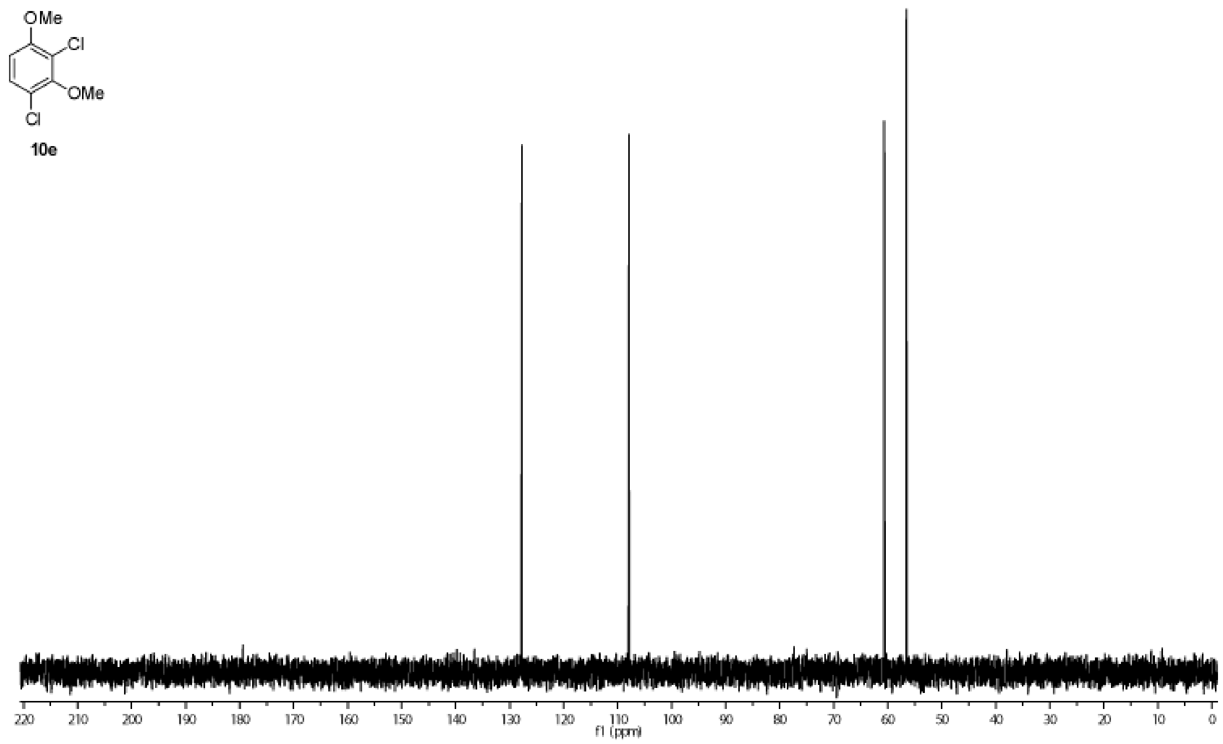

Figure 35: ^{13}C DEPT spectrum of compound **10e**.

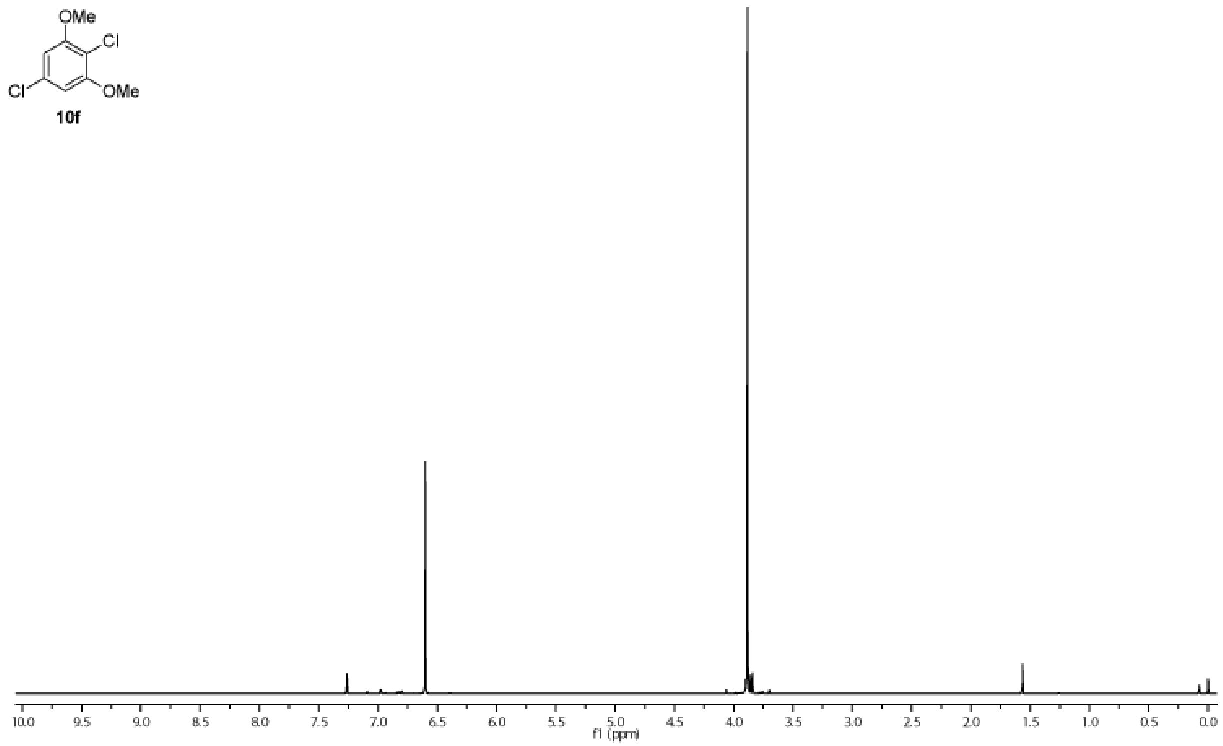

Figure 36: ^{1}H NMR spectrum of compound **10f**.

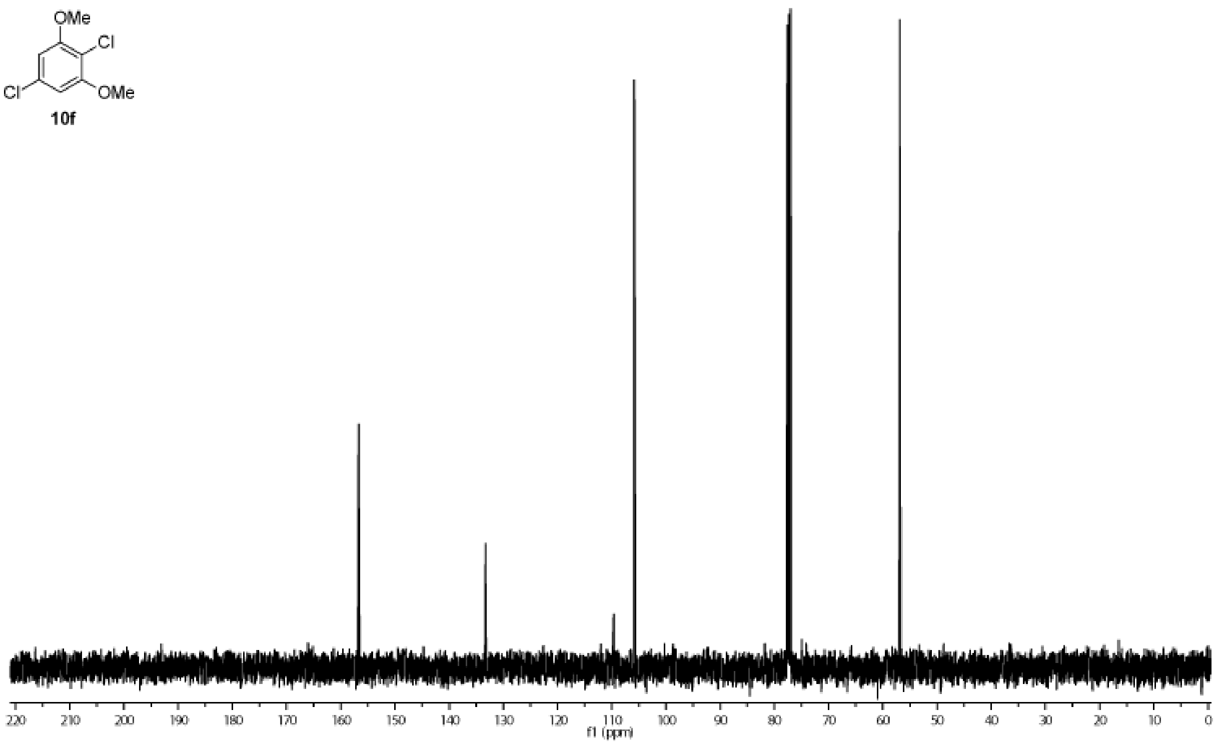

Figure 37: ^{13}C NMR spectrum of compound **10f**.

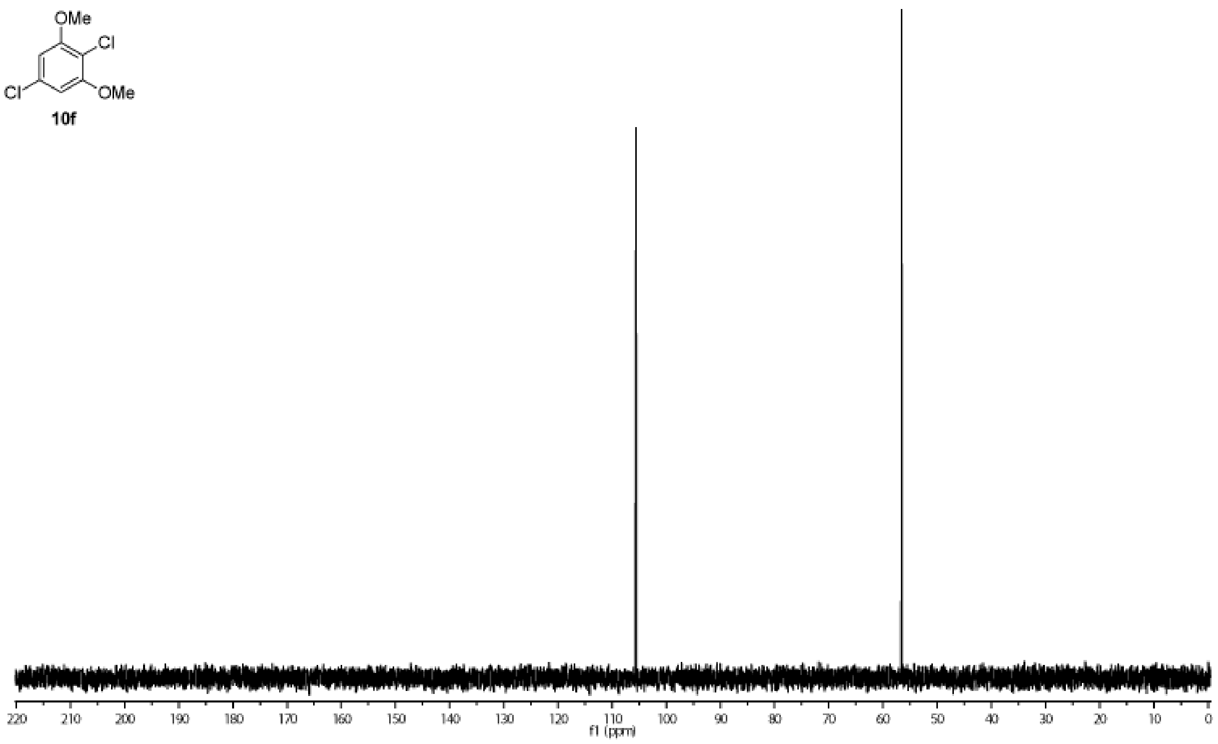

Figure 38: ^{13}C DEPT spectrum of compound **10f**.

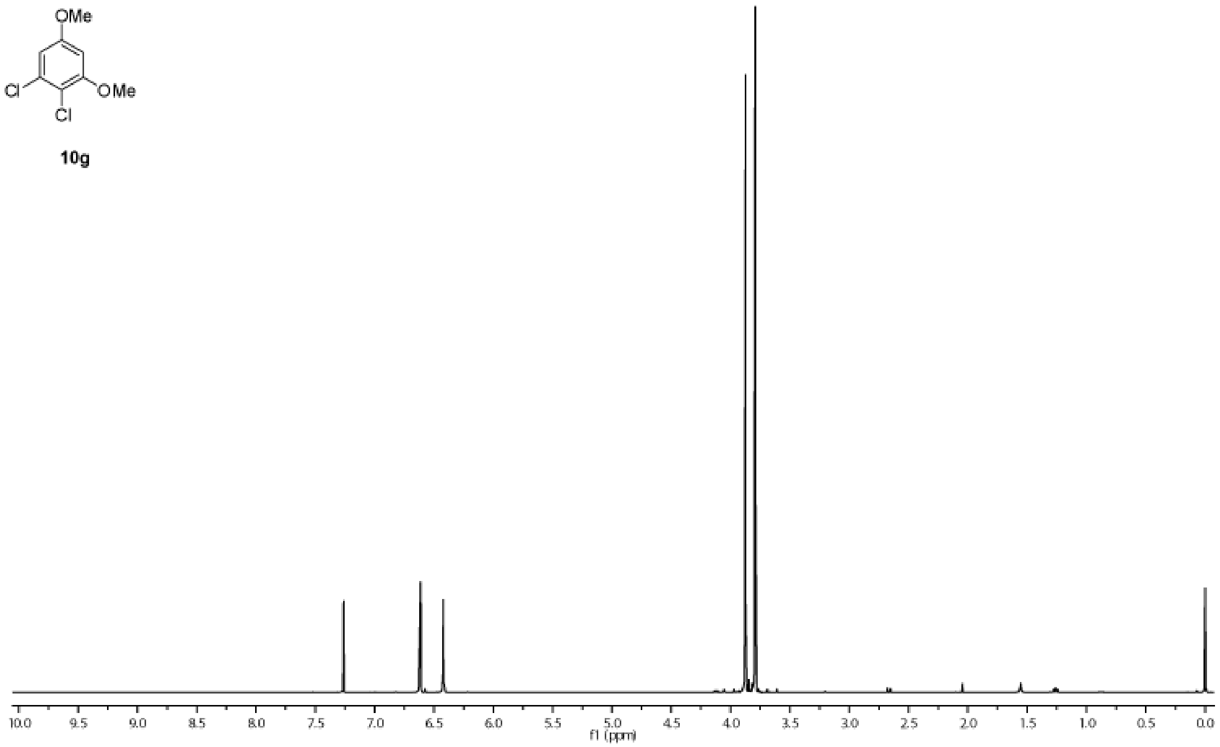

Figure 39: ^{1}H NMR spectrum of compound **10g**.

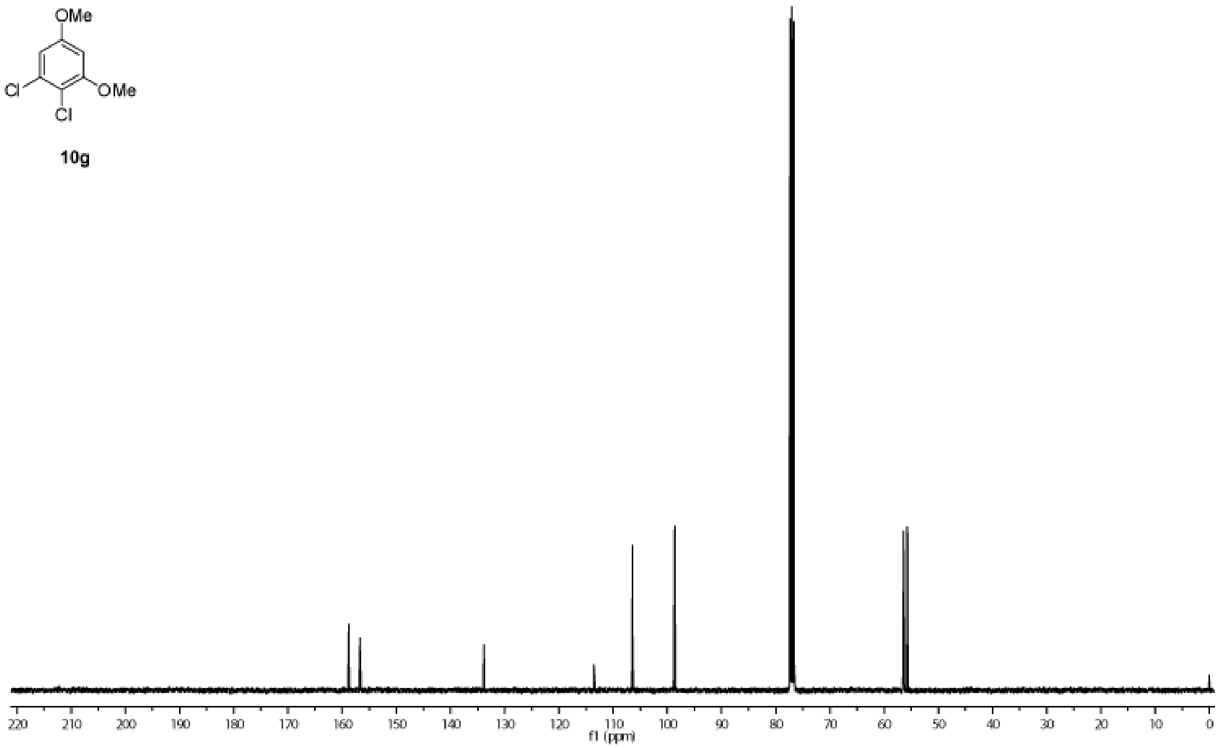

Figure 40: ^{13}C NMR spectrum of compound **10g**.

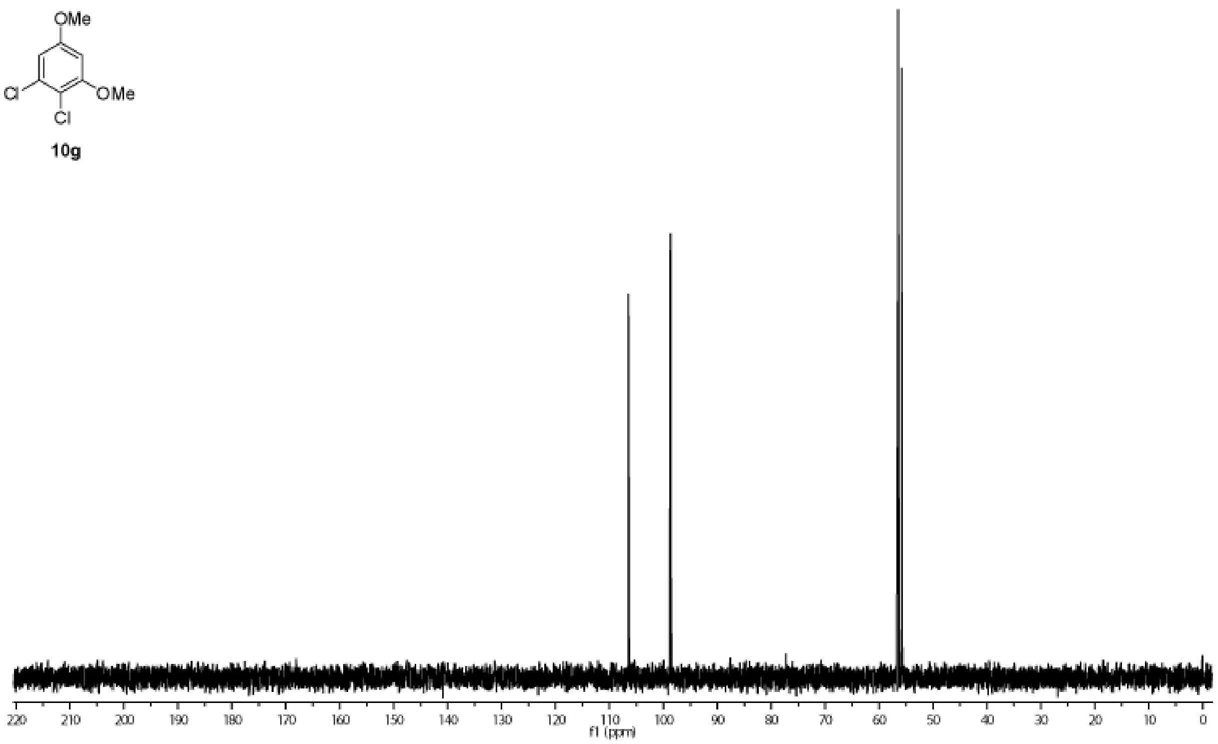

Figure 41: ^{13}C DEPT spectrum of compound **10g**.

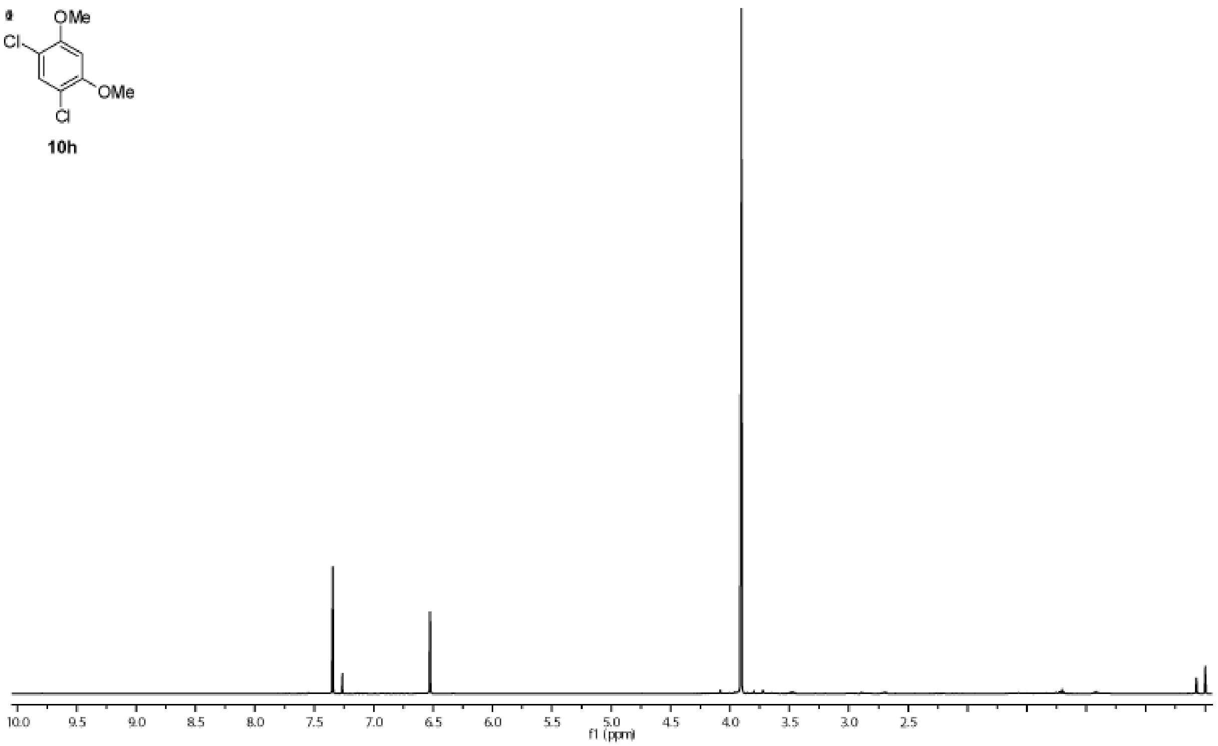

Figure 42: ^{1}H NMR spectrum of compound **10h**.

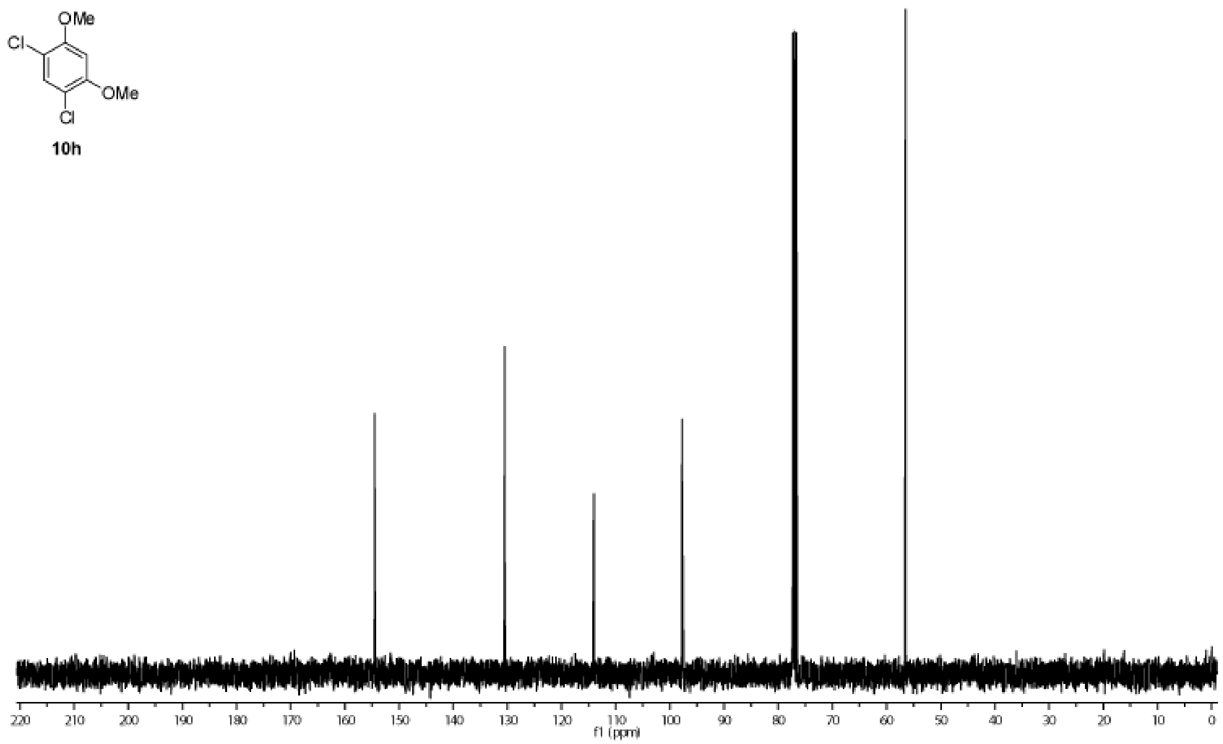

Figure 43: ^{13}C NMR spectrum of compound **10h**.

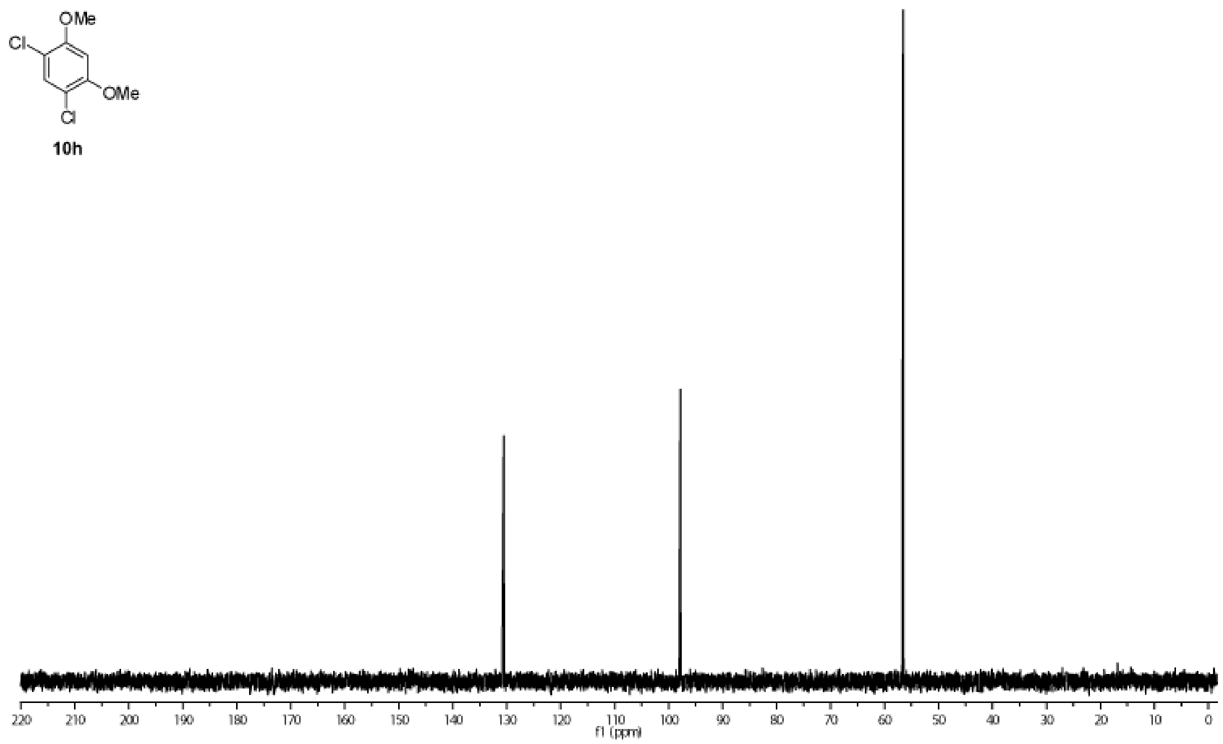

Figure 44: ^{13}C DEPT spectrum of compound **10h**.

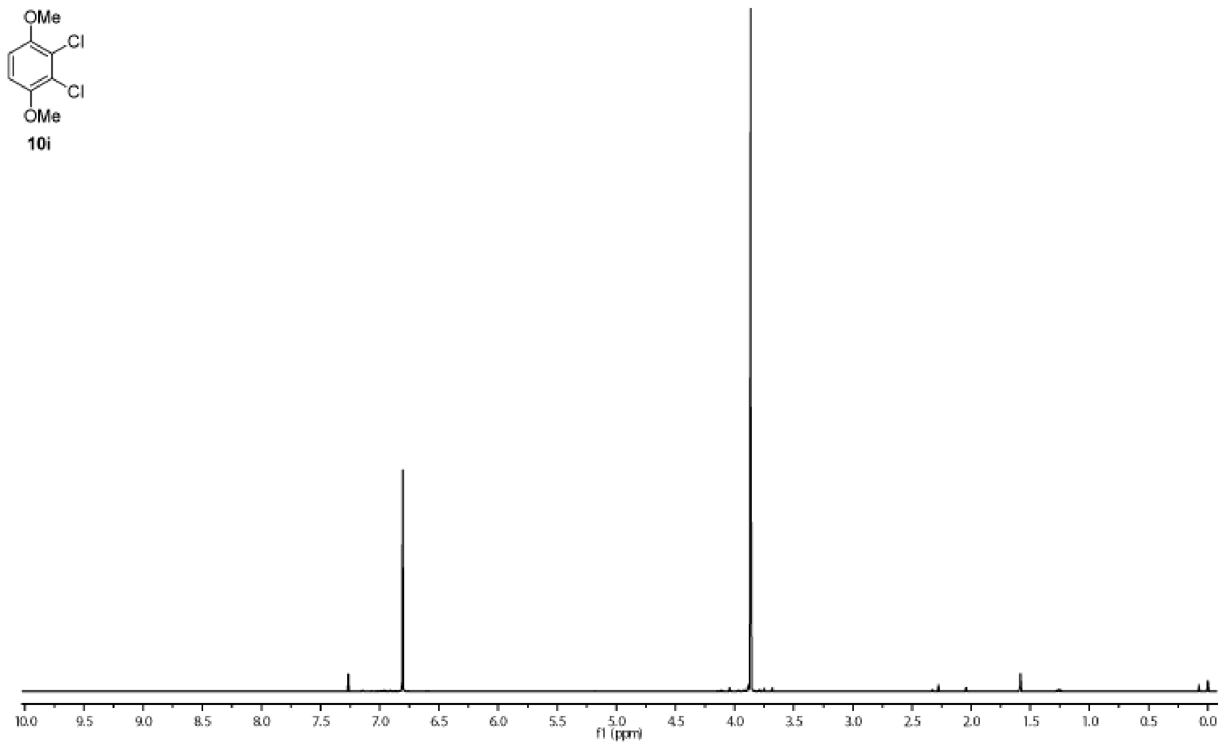

Figure 45: ^{1}H NMR spectrum of compound **10i**.

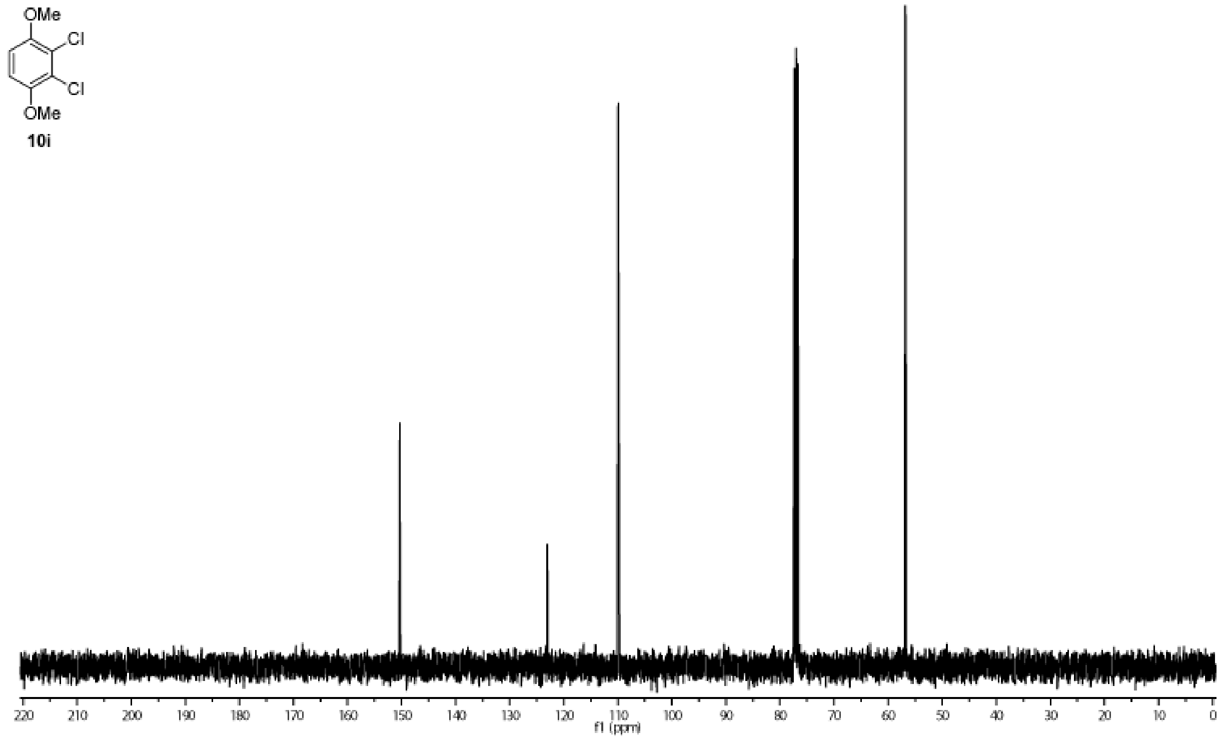

Figure 46: ^{13}C NMR spectrum of compound **10i**.

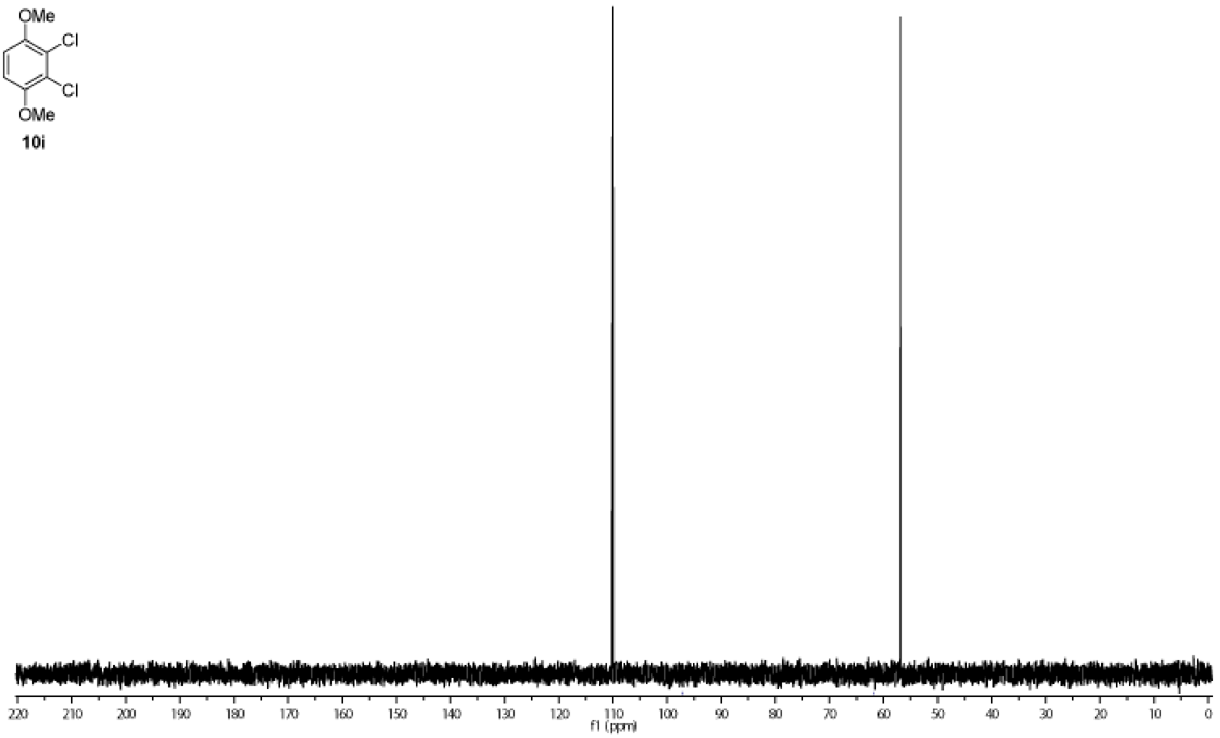

Figure 47: ^{13}C DEPT spectrum of compound **10i**.

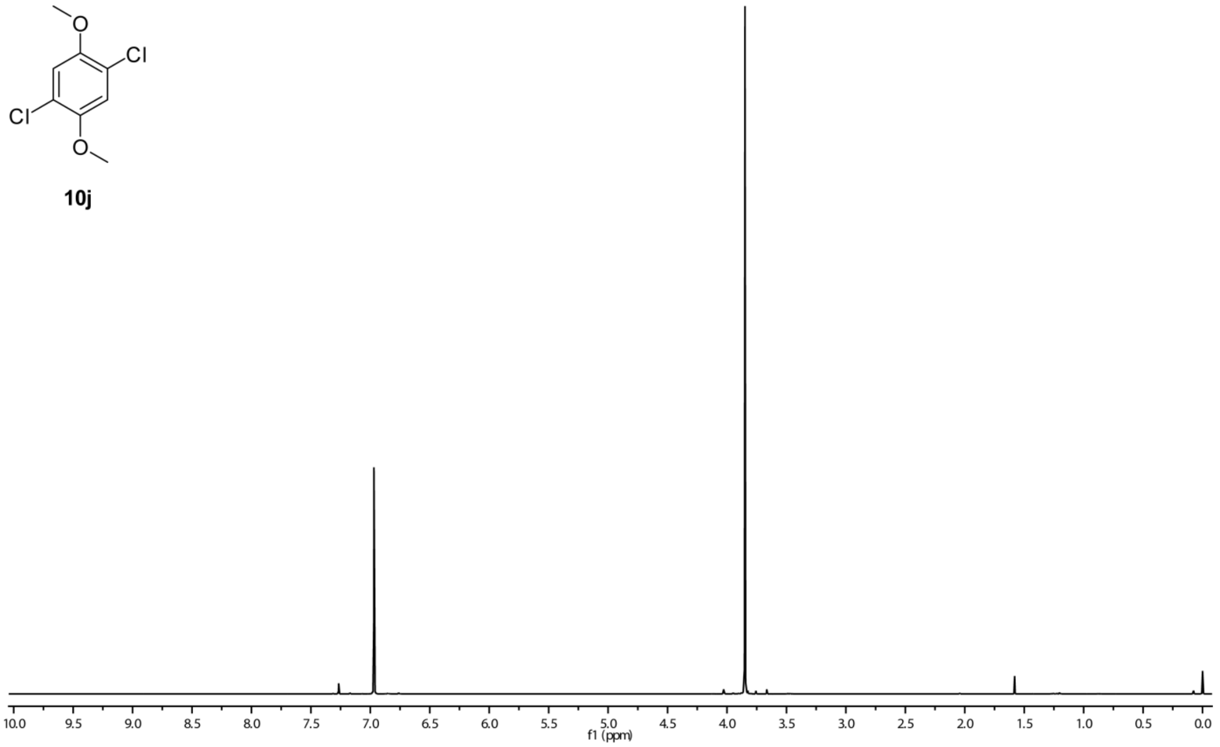

Figure 48: ^{1}H NMR spectrum of compound **10j**.

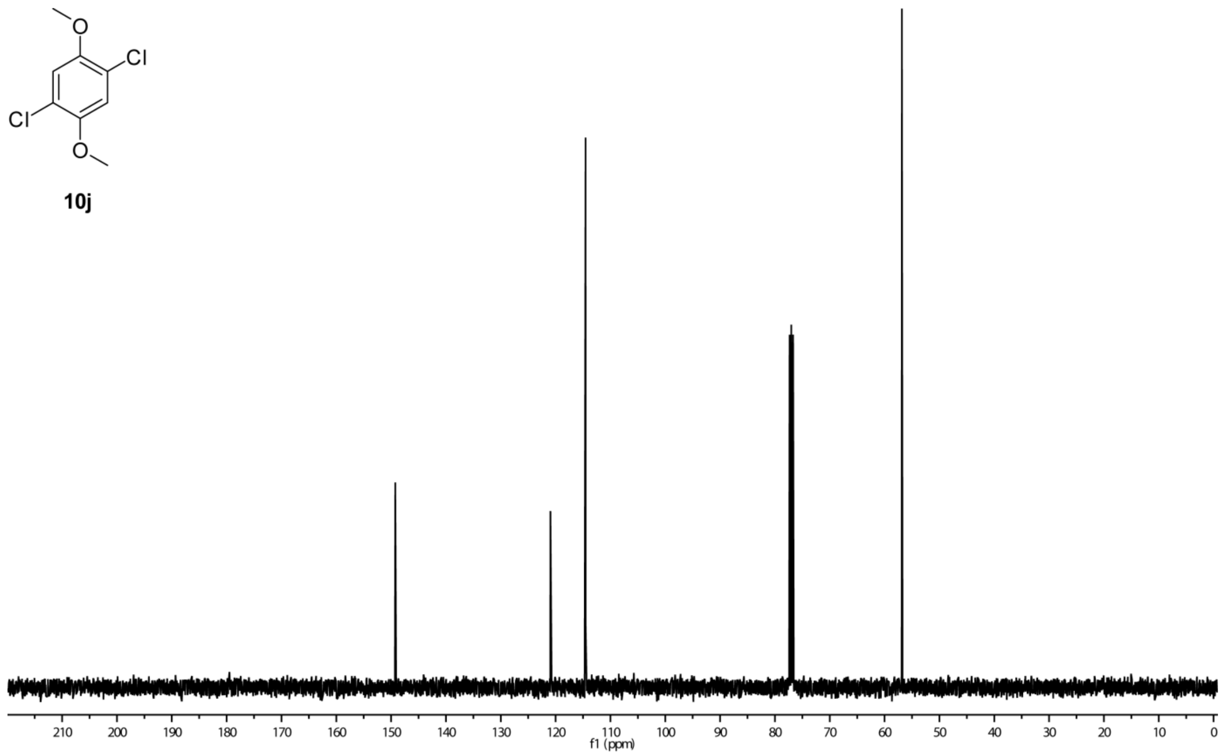

Figure 49: ^{13}C NMR spectrum of compound **10j**.

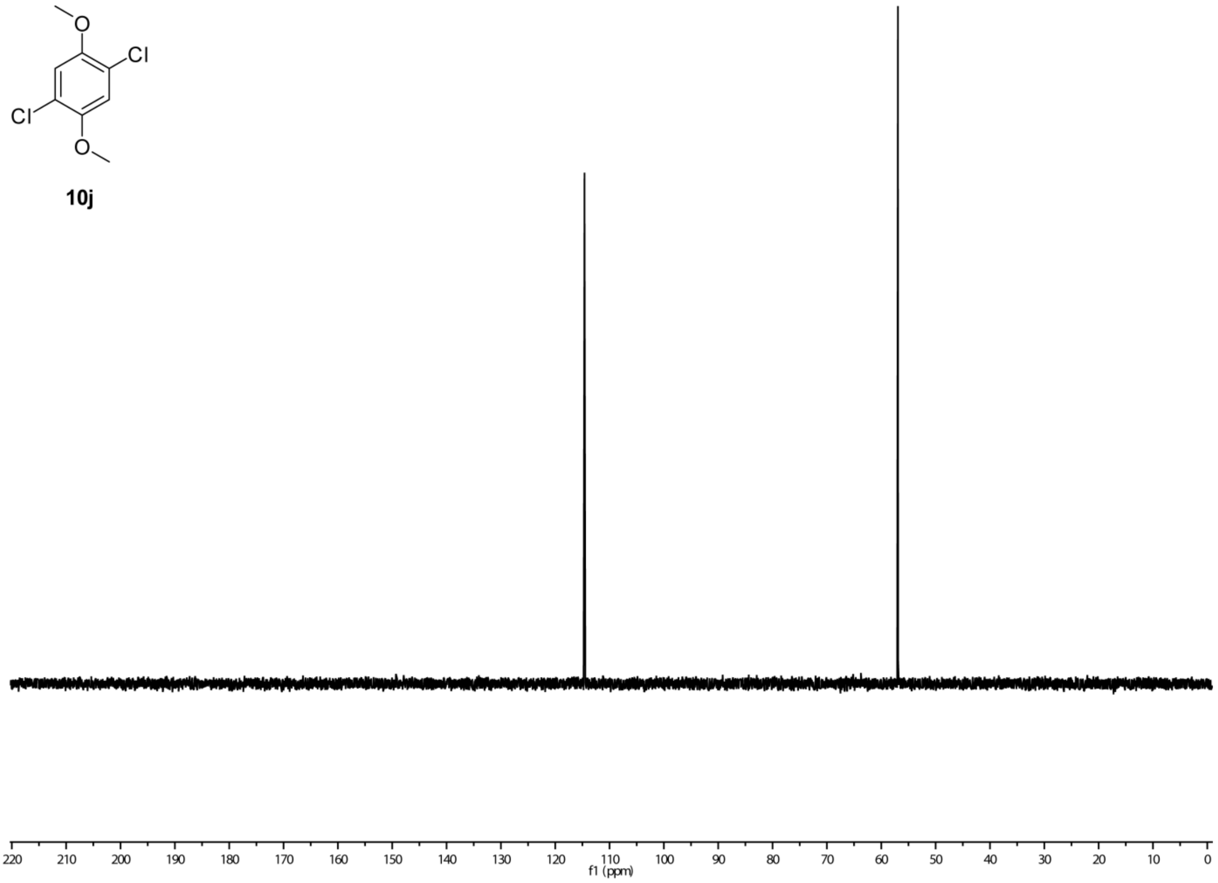

Figure 50: ^{13}C DEPT spectrum of compound **10j**.

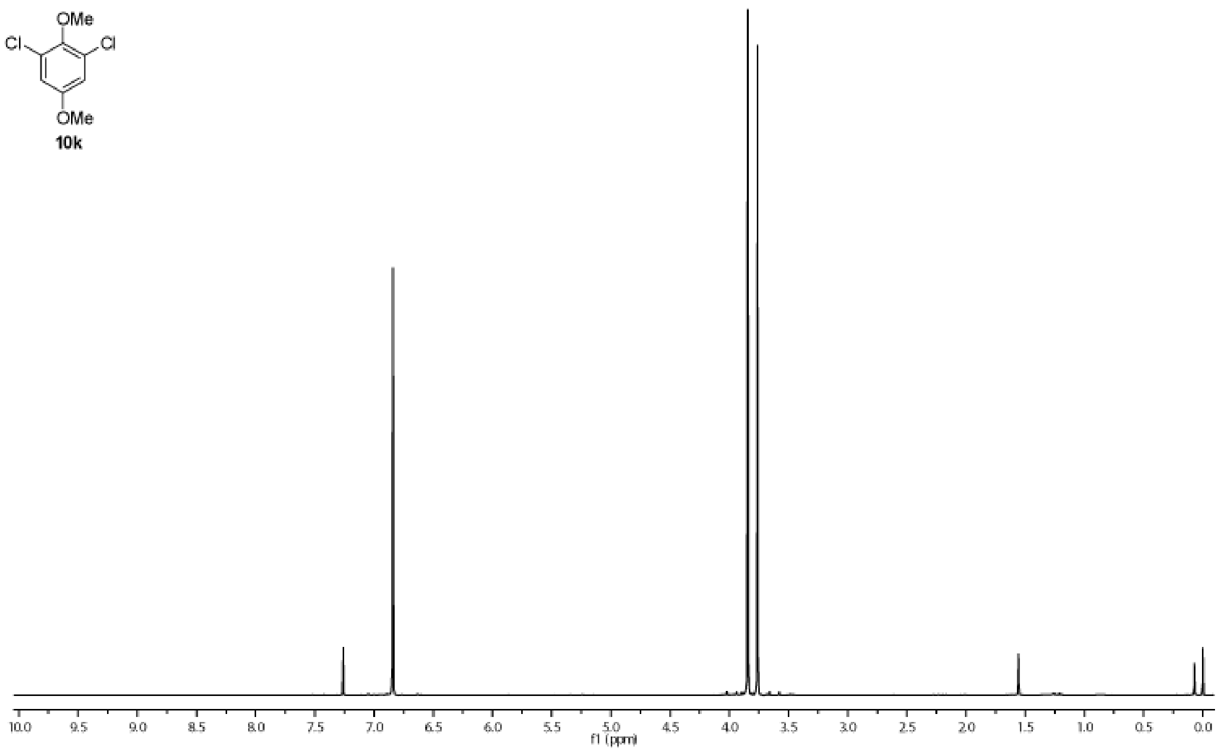

Figure 51: ^{1}H NMR spectrum of compound **10k**.

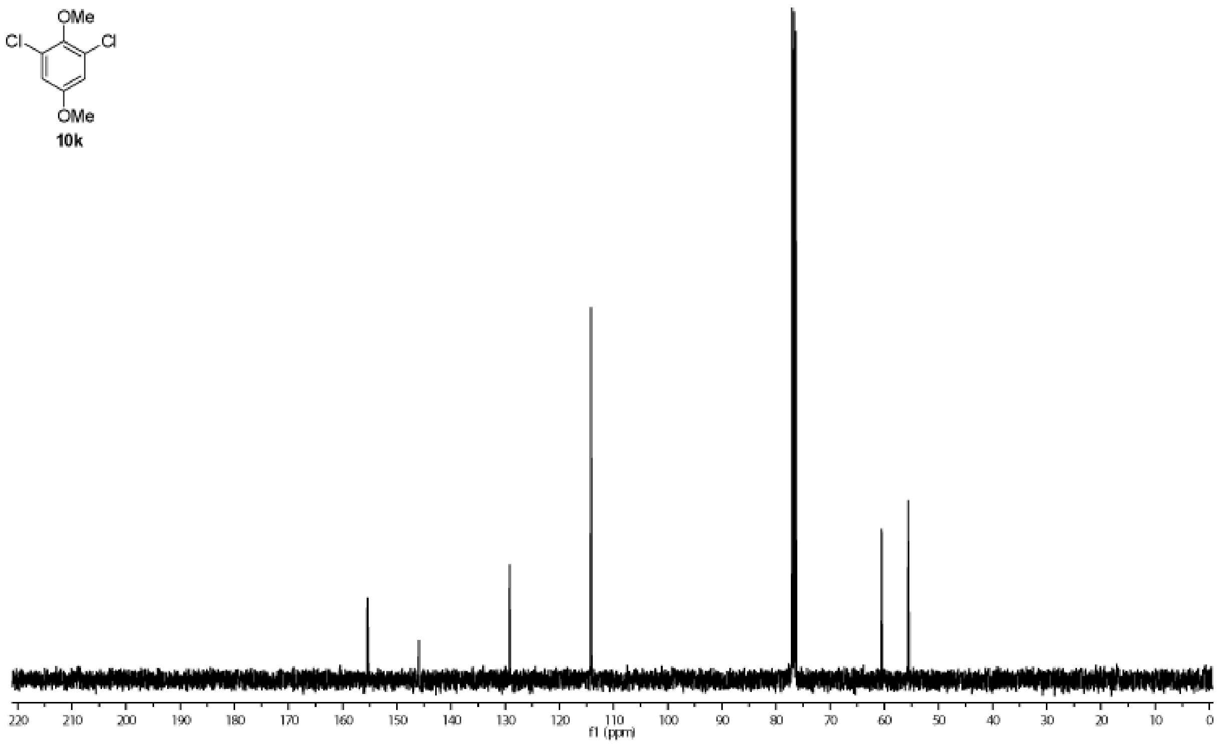

Figure 52: ^{13}C NMR spectrum of compound **10a**.

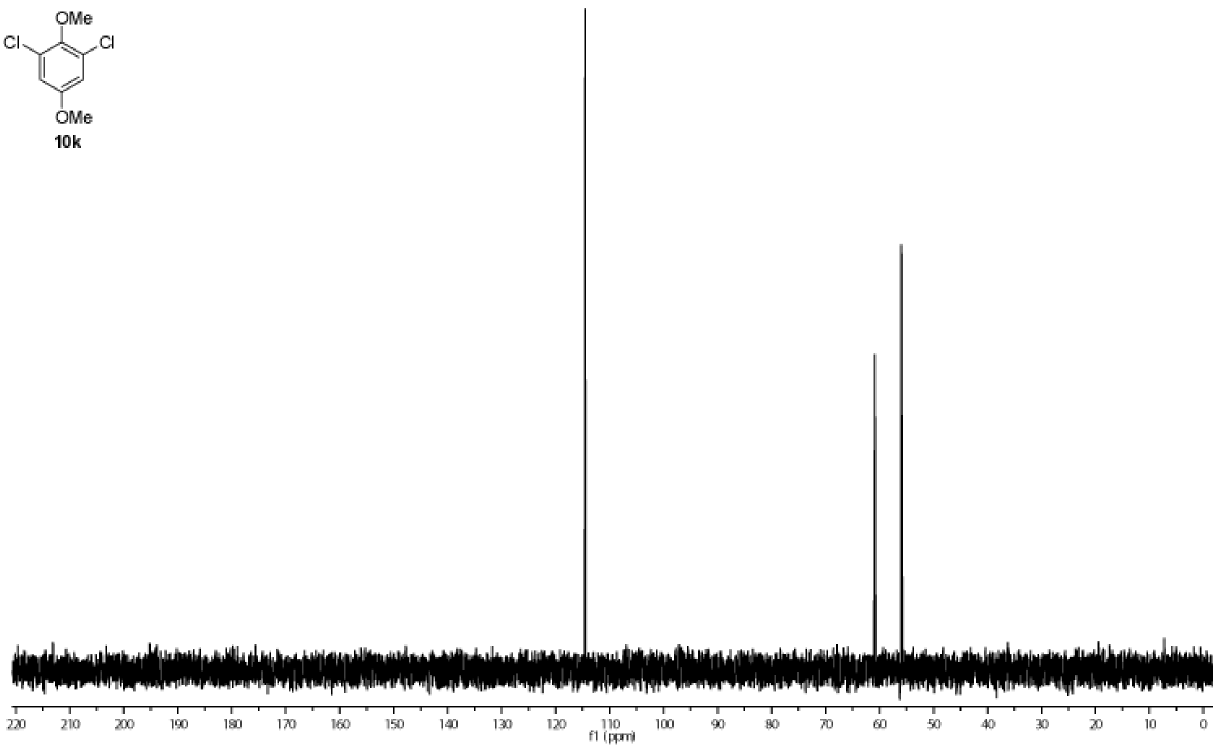

Figure 53: ^{13}C DEPT spectrum of compound **10k**.

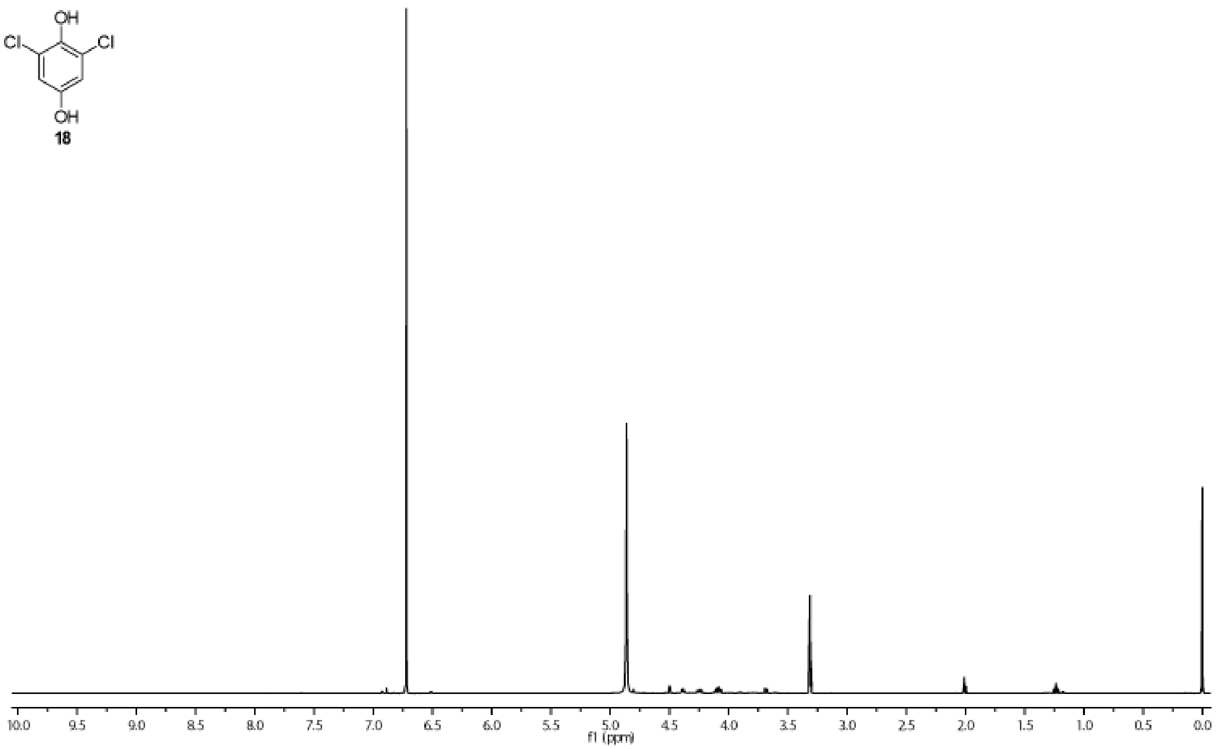

Figure 54: ^{1}H NMR spectrum of compound **18**.

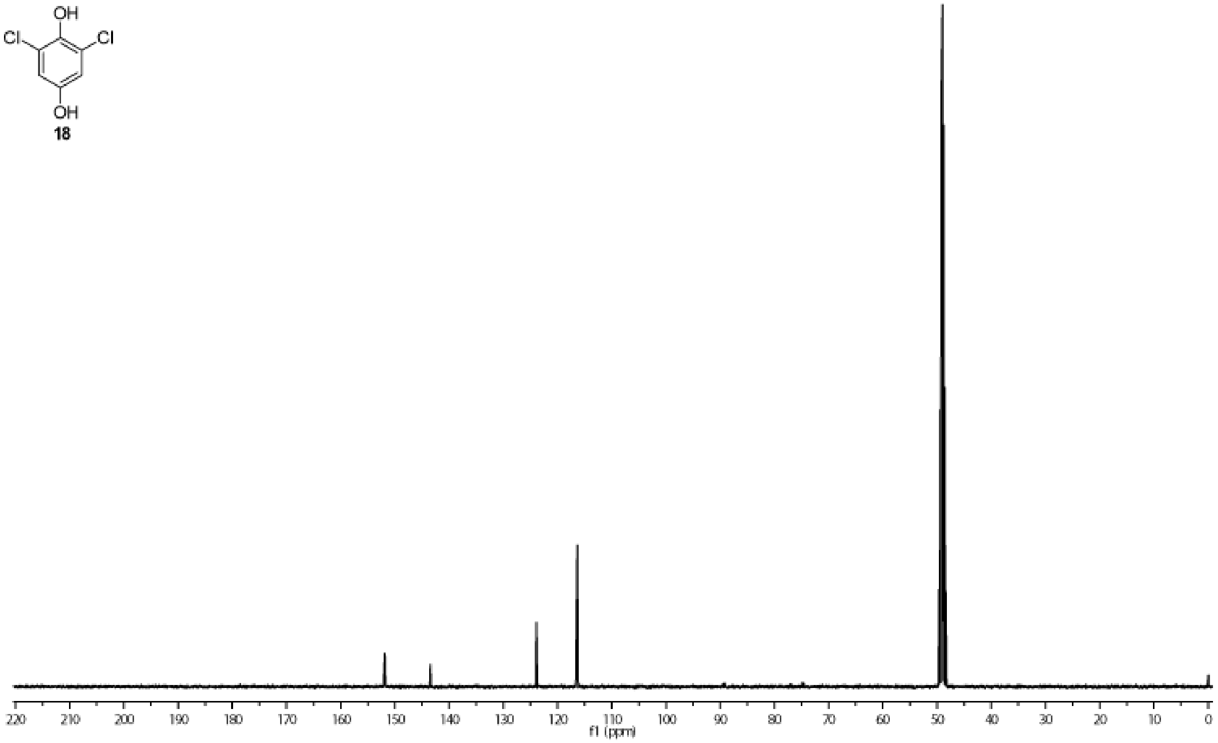

Figure 55: ^{13}C NMR spectrum of compound **18**.

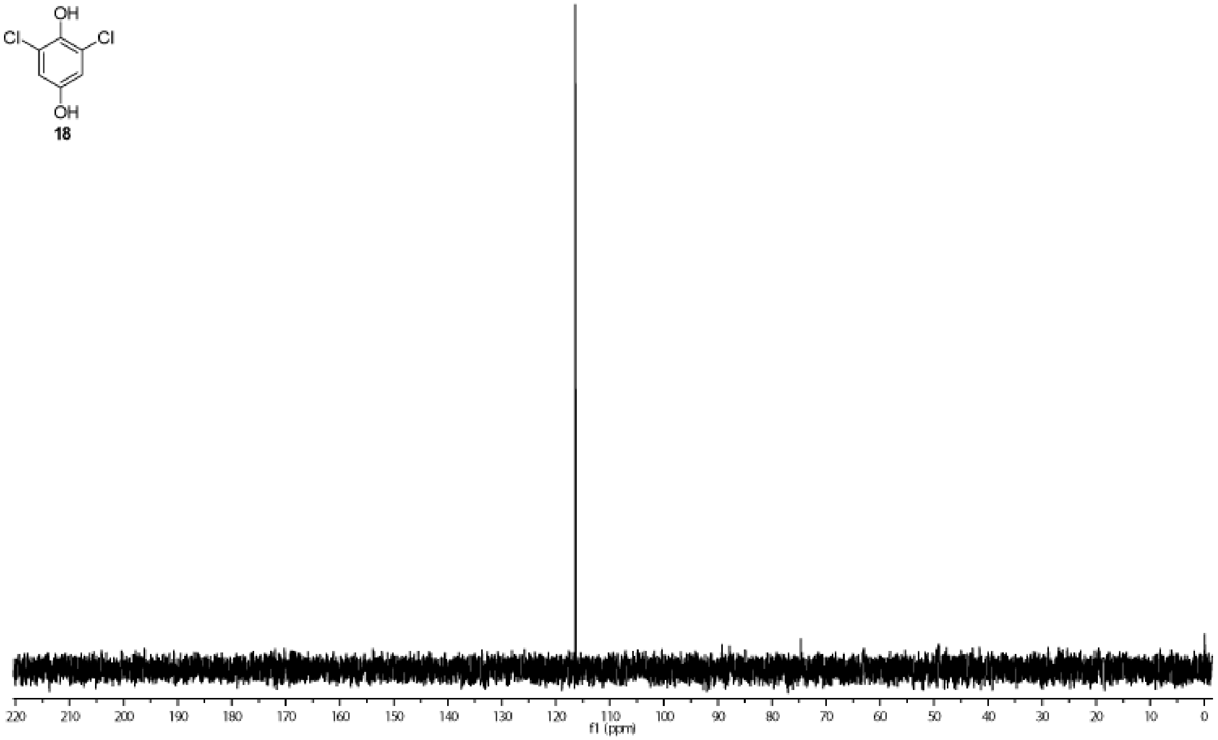

Figure 56: ^{13}C DEPT spectrum of compound **18**.
